# Safety and efficacy of thalidomide in patients with transfusion-dependent β-thalassemia: a randomized clinical trial

**DOI:** 10.1038/s41392-021-00811-0

**Published:** 2021-11-18

**Authors:** Jiang-Ming Chen, Wei-Jian Zhu, Jie Liu, Gui-Zhen Wang, Xiao-Qin Chen, Yun Tan, Wei-Wei Xu, Li-Wei Qu, Jin-Yan Li, Huan-Ju Yang, Lan Huang, Ning Cai, Wei-Da Wang, Ken Huang, Jian-Quan Xu, Guo-Hui Li, Sheng He, Tian-Ying Luo, Yi Huang, Song-Hua Liu, Wen-Qiang Wu, Qi-Yang Lu, Mei-Guang Zhou, Shu-Ying Chen, Rong-Lan Li, Mei-Ling Hu, Ying Huang, Jin-Hua Wei, Jun-Min Li, Sai-Juan Chen, Guang-Biao Zhou

**Affiliations:** 1 Department of Hematology, Wuzhou Gongren Hospital, Wuzhou, 543001 Guangxi China; 2 Department of Hematology, Zhuhai People’s Hospital (Zhuhai Hospital Affiliated with Jinan University), Zhuhai, 541000 Guangdong China; 3 State Key Laboratory of Molecular Oncology, National Cancer Center/National Clinical Research Center for Cancer/Cancer Hospital, Chinese Academy of Medical Sciences and Peking Union Medical College, Beijing, 100021 China; 4 Department of Reproductive Medicine, The First Affiliated Hospital of Hebei North University, Zhangjiakou, 075000 Hebei China; 5 State Key Laboratory of Oncology in South China, Collaborative Innovation Center for Cancer Medicine; Medical Oncology Department, Sun Yat-Sen University Cancer Center, Guangzhou, 510060 Guangdong China; 6 State Key Laboratory of Medical Genomics, Shanghai Institute of Hematology, National Research Center for Translational Medicine, Ruijin Hospital Affiliated with Shanghai Jiao Tong University School of Medicine, Shanghai, 200025 China; 7 State Key Laboratory of Membrane Biology, Institute of Zoology, Chinese Academy of Sciences & University of Chinese Academy of Sciences, Beijing, 100101 China; 8 Department of Pediatrics, Affiliated Hospital of Youjiang Medical University for Nationalities, Baise City, 533000 Guangxi China; 9 Department of Hematology, Yulin Guinan Hospital, Yulin, 537005 Guangxi China; 10 Department of neurology, Wuzhou Gongren Hospital, Wuzhou, 543001 Guangxi China; 11 Guangxi Key Laboratory of Basic Research on Birth Defects Prevention and Treatment, Guangxi Zhuang Autonomous Region Women and Children Health Care Hospital, Nanning, 530000 Guangxi China; 12 Department of Hematology, Guigang People’s Hospital, Guigang, 537100 Guangxi China; 13 Department of Hematology, Hospital of Traditional Chinese Medicine of Wuzhou City, Wuzhou, 543002 Guangxi China; 14 Department of Hematology, Wuzhou Red Cross Hospital, Wuzhou, 543002 Guangxi China; 15 Department of Hematology, Hechi People’s Hospital, Hechi City, 547000 Guangxi China

**Keywords:** Haematological cancer, Translational research

## Abstract

Thalidomide induces γ-globin expression in erythroid progenitor cells, but its efficacy on patients with transfusion-dependent β-thalassemia (TDT) remains unclear. In this phase 2, multi-center, randomized, double-blind clinical trial, we aimed to determine the safety and efficacy of thalidomide in TDT patients. A hundred patients of 14 years or older were randomly assigned to receive placebo or thalidomide for 12 weeks, followed by an extension phase of at least 36 weeks. The primary endpoint was the change of hemoglobin (Hb) level in the patients. The secondary endpoints included the red blood cell (RBC) units transfused and adverse effects. In the placebo-controlled period, Hb concentrations in patients treated with thalidomide achieved a median elevation of 14.0 (range, 2.5 to 37.5) g/L, whereas Hb in patients treated with placebo did not significantly change. Within the 12 weeks, the mean RBC transfusion volume for patients treated with thalidomide and placebo was 5.4 ± 5.0 U and 10.3 ± 6.4 U, respectively (*P* < 0.001). Adverse events of drowsiness, dizziness, fatigue, pyrexia, sore throat, and rash were more common with thalidomide than placebo. In the extension phase, treatment with thalidomide for 24 weeks resulted in a sustainable increase in Hb concentrations which reached 104.9 ± 19.0 g/L, without blood transfusion. Significant increase in Hb concentration and reduction in RBC transfusions were associated with non *β0*/*β0* and *HBS1L*-*MYB* (rs9399137 C/T, C/C; rs4895441 A/G, G/G) genotypes. These results demonstrated that thalidomide is effective in patients with TDT.

## Introduction

β-thalassemia (BTM), one of the commonest monogenic hereditary diseases worldwide, is caused by mutations in the gene encoding β-chains of the hemoglobin (Hb) and is characterized by the reduced or absent synthesis of β-globin, ineffective erythropoiesis, and hemolysis of mature red blood cells (RBCs) caused by excess α-chains.^1–3^ BTM was classified as subtypes major, intermediate, and minor, and is also divided into blood transfusion-dependent thalassemia (TDT) and non-transfusion-dependent thalassemia (NTDT). Each year, there will be more than 40,000 BTM babies including 26,000 TDTs born worldwide, mostly in resource-constrained countries.^1,4^ Currently, approximately 100,000 patients receive regular transfusions worldwide. Other treatment regimens for BTM mainly include iron chelation, hematopoietic stem cell transplantation (HSCT),^4^ the emerging gene therapy,^5^ and erythroid maturation agent.^6^ It is estimated that only 12% of children with TDT receive adequate blood transfusion, and < 40% of those transfused receive adequate iron chelation therapy. Though HSCT is curative for those under 14 years of age at transplantation, it is available for less than 30% patients and has a 5–10% treatment related mortality for the recipients.^1,4^ Gene therapy represents a promising therapy, but its long-term efficacy remains to be determined.^5^ Therefore, effective and affordable remedies are urgently needed to save patients with TDT, especially for those who are 14 years of age and older.

Inducing production of compensative fetal Hb (Hb F) is an emerging treatment option for BTM, whose efficacy is associated with single nucleotide polymorphisms (SNPs) in the Gγ globin gene *HBG2*^7^ (also known as Xmn I polymorphism that shows a G > A transition at position −158 of the gene), *BCL11A*^8^ and *HBS1L-MYB* intergenic region.^9^ The old and multidimensional drug thalidomide has been shown to be an Hb F inducer^1^ that can significantly increase Hb (mainly Hb F) concentration in TDT patients in several case reports.^10–12^ Chen et al.^13^ reported that in 9 patients treated with thalidomide, Hb concentration and Hb F ratio increased from 51.3 ± 21.5 g/L and 35.7 ± 26.8% before treatment to 103.8 ± 11.9 g/L and 75.7 ± 14.6% after treatment, respectively. In these patients, Hb increment was usually seen in 1 month upon thalidomide treatment.^13,14^ However, these clinical data were case reports or small-scale single-center studies, while the efficacy of other Hb F inducers (including hydroxyurea^15,16^ and butyrate^17^) was not satisfactory. Here, we described a phase II, multicenter, randomized, double-blinded, placebo-controlled clinical trial, followed by an extension phase, to evaluate the efficacy and safety of thalidomide in the treatment of patients with TDT.

## Results

### Patient characteristics

**Fig. 1:**
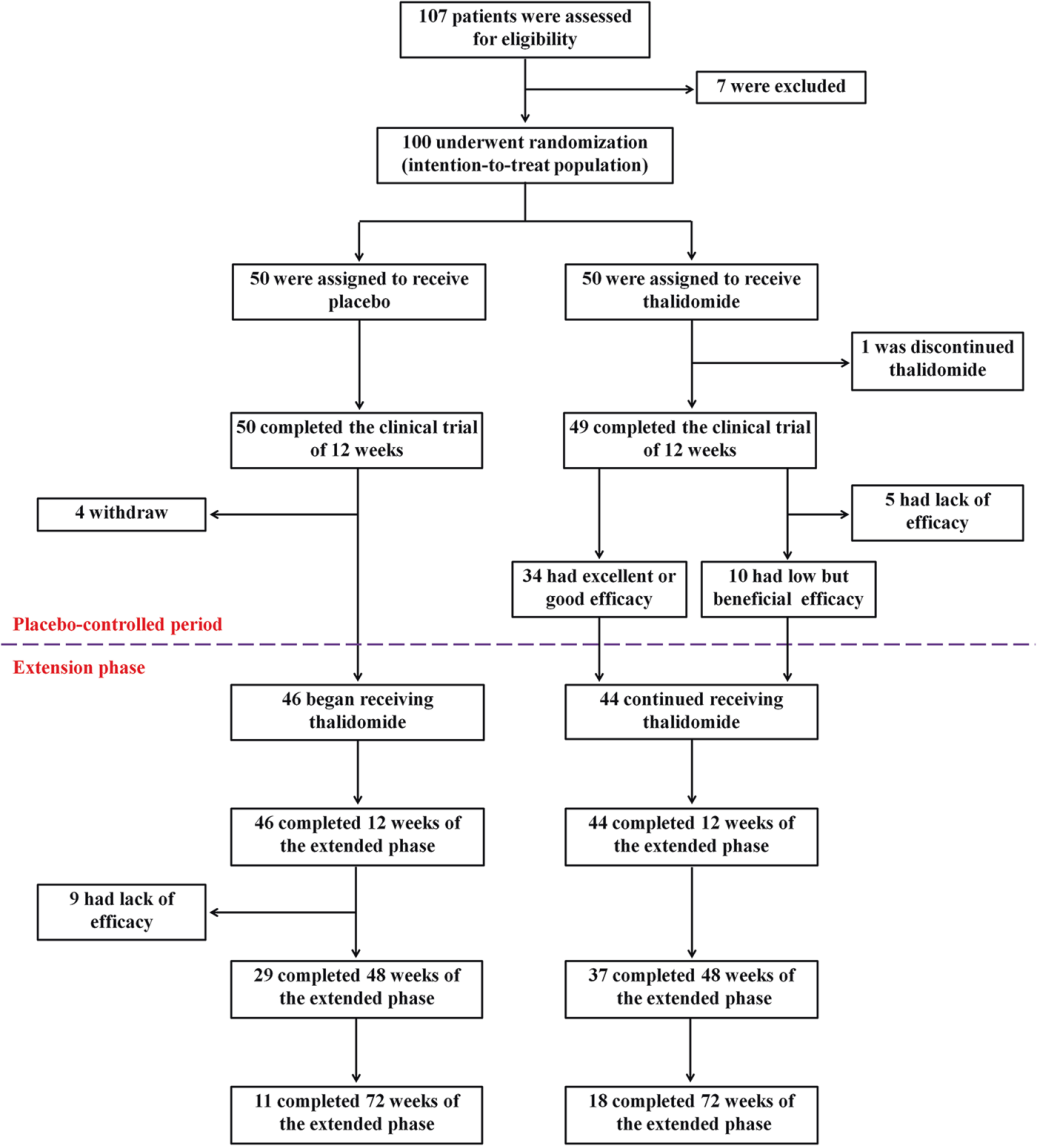
Study design

TDT patients who were 14 years of age or older were enrolled in this study between May 2^nd^ 2018 to October 24^th^ 2019. A total of 107 participants were assessed for eligibility at 6 centers in Southern China, and 100 of them were randomized to receive placebo (*n* = 50) or thalidomide (*n* = 50) treatment (Fig. 1). The average age was 18.4 years, 63% of them were male and 37% were female. For genotyping, 28% had a β0/β0, 75% had an Xmn I G/G, 61% had a *HBS1L-MYB* T/T (rs9399137), and 95% had a C/C *BCL11A* (rs11886868) BTM genotypes (Table 1, Supplementary Tables 1 and 2). Thirty-nine patients received splenectomy (Table 1), while rare cases obtained regular iron chelation therapy. One patient (29 years old, male) in thalidomide group had a stroke on day 17, 15 h after taking the 16^th^ dose of thalidomide. He had a slightly decreased fibrinogen (1.44 g/L; reference range: 1.8–3.5 g/L) and a slightly prolonged activated partial thromboplastin time (APTT; 39.7 s; reference range: 21.1–36.5 s). Computed tomography analysis revealed a chronic state of vascular encephalopathy for a right insular atrophy and an atrophic dilation of the III ventricle, and computed tomography angiography of brain vessels showed abnormally development of the right anterior and middle cerebral arteries and stenosis of the left and right cerebral arteries (Supplementary Fig. 1). He was diagnosed as ischemic stroke and was withdrawn and received symptomatic treatment and recovered in three days. The other 99 patients completed the trial and 90 (90.9%) of them were included in the extension phase (Fig. 1).

**Table 1:** Baseline demographic characteristics of the patients

	Total (*n* = 100)	Placebo (*n* = 50)	Thalidomide (*n* = 50)
Age, y	18.4 ± 5.6	18.4 ± 5.8	18.4 ± 5.5
Sex, *n* (%)			
Male	63 (63)	33 (66)	30 (60)
Female	37 (37)	17 (34)	20 (40)
Genotype, *n* (%)			
β0/β0	28 (28)	19 (38)	9 (18)
β0/non-β0	59 (59)	25 (50)	34 (68)
non-β0/non-β0	13 (13)	6 (12)	7 (14)
Xmn I genotype, *n* (%)			
G/G	75 (75)	38 (76)	37 (74)
A/G	25 (25)	12 (24)	13 (26)
Simultaneous α globin mutation	4	3	1
Splenectomy, *n* (%)			
Yes	39 (39)	26 (52)	13 (26)
No	61 (61)	24 (48)	37 (74)
Hb, g/L	73.6 ± 16.3	75.7 ± 14.9	71.4 ± 17.5
Hb F, %	12.9 (4.9, 34.7)	14.0 (4.7, 39.0)	10.8 (5.1, 28.9)
Red blood cell count, 10^12^/L	3.0 ± 0.6	3.0 ± 0.5	3.0 ± 0.7
Reticulocytes (%)	3.1 (1.1, 10.8)	2.8 (1.3, 13.1)	3.2 (0.6, 8.0)
Erythrocyte life span, d	13.4 ± 7.7	13.6 ± 7.8	13.2 ± 7.6
EPO level	288.6 ± 264.9	266.1 ± 254.3	310.1 ± 275.6
Total bilirubin, µM	46.2 (32.6, 59.3)	42.6 (32.0, 57.9)	48.4 (33.2, 63.0)
Indirect bilirubin, µM	35.4 (24.5, 45.0)	33.2 (22.8, 44.4)	35.4 (25.4, 48.2)
Age at first transfusion, years	4.2 ± 6.1	3.9 ± 6.1	4.4 ± 6.2
Red cells transfused in previous 1 year, U	42.2 ± 24.5	41.7 ± 24.8	42.8 ± 24.4
Mean Hb in previous 1 year, g/L	69.2 ± 16.6	68.7 ± 16.7	69.6 ± 16.6

*Hb F* fetal hemoglobin, *EPO* erythropoietin

### Hematological response

**Fig. 2:**
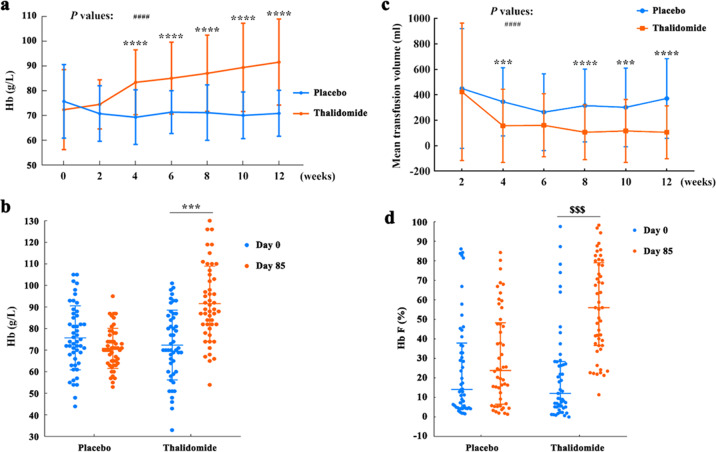
Changes in Hb, transfusion volume, and Hb F in patients upon placebo or thalidomide treatment. The levels of Hb (**a**, **b**) and transfusion volume (**c**) were expressed as mean ± standard deviation (SD), and *P* values determined by Student’s *t* test (*) and mixed-effects regression analyses (#) were provided. ***P < 0.001, ****P < 0.0001; ####*P* < 0.0001. The percentages of Hb F (**d**) were expressed as median (interquartile range), and *P* value was determined by Mann–Whitney *U* test. $$$*P* < 0.001

The Hb levels of the patients upon thalidomide rose from 72.4 ± 16.2 to 91.6 ± 17.4 g/L, with a median elevation of 14.0 (range, 2.5 to 37.5) g/L in 12 weeks, whereas the Hb levels of patients treated with placebo did not increase (Fig. 2a, b). Among the 49 thalidomide-treated patients, 20 (40.8%) achieved excellent response (defined as an elevation in Hb level of ≥ 20 g/L was achieved and the patients were free of transfusion for at least 6 weeks), 14 (28.6%) achieved good response (an increase in Hb concentration reached 10 to 20 g/L, or Hb was not substantially increased but the patients had Hb > 70 g/L and were free of transfusion for at least 6 weeks), and 15 (30.6%) exhibited no response (an elevation in Hb level < 10 g/L and the patients were still transfusion-dependent) (Supplementary Table 3). Upon thalidomide, the median increase in Hb were 5.0 (−2.5, 14.5), 20.0 (3.8, 40.3), and 18.0 (−6.8, 41.0) g/L in patients with β0/β0, β0/non-β0, and non-β0/non-β0 BTM, respectively (Table 2). We reported that 17 (50%) and 9 (26.5%) of the 34 β0/non-β0 BTMs achieved excellent and good responses, respectively, which were higher than those in β0/β0 BTMs (Supplementary Table 4). *HBG2*, *HBS1L-MYB*, and *BCL11A* genotypes did not show impact on the patients’ response to thalidomide, possibly due to the small number of patients in each subtype (Supplementary Table 4). Thalidomide was effective in patients who received splenectomy as well as those did not receive splenectomy (Supplementary Table 5). Furthermore, mixed-effects regression analyses indicated that thalidomide increased Hb levels to a greater extent than placebo over 12 weeks (SE = 0.001, *T*-value=13.441, *P* = 3.92E-36).

**Table 2:** Genotype and change in Hb. Data are expressed as median (range)

Genotype	Placebo		Thalidomide		*P*
	*n*	Hb_d85_-Hb_d0_ (g/L)	*n*	Hb_d85_–Hb_d0_ (g/L)	
All patients	50	−5.5 (−10.5, 2)	49	14 (2.5, 37.5)	0.000
β0/β0	19	−7 (−10, 2)	9	5 (−2.5, 14.5)	0.047
β0/non-β0	25	−5 (−13.5, 0.5)	34	20 (3.8, 40.3)	0.000
non-β0/non-β0	6	−2.5 (−9.8, 13)	6	18 (−6.8, 41)	0.187

*Hb*_*d85*_ Hb level of day 85 (after treatment with thalidomide for 12 weeks), *Hb*_*d0*_ Hb level of day 0 (baseline level). *P* values were determined by Mann–Whitney *U* test

### Hb components and blood cells

Thalidomide significantly increased the Hb F ratio of the patients, which rose from 10.8% to 55.2% in 12 weeks (Table 1, Fig. 2d and Supplementary Table 6). Elevation of Hb F was more frequently seen in non-β0/non-β0 and β0/non-β0 BTMs (Supplementary Table 7). Thalidomide prolonged erythrocyte life span (ELS) (Supplementary Table 6), but did not significantly perturb white blood cells and platelets in the patients (Supplementary Table 8).

### Reduction in transfusion requirement

Thalidomide significantly reduced the amount of blood transfusion required for the patients (Fig. 2c). Within the 12 weeks, the mean transfusion volume for patients treated with placebo and thalidomide was 10.3 ± 6.4 U (2 060 ± 1 280 ml) and 5.4 ± 5.0 U (1 080 ± 1 000 ml), respectively (*P* < 0.001; Supplementary Table 6). Mixed-effects regression analyses confirmed that thalidomide significantly reduced transfusion volume to a greater extent than placebo over 12 weeks (*P* = 3.54E-9). The proportion of patients who needed blood transfusion to maintain Hb ≥70 g/L gradually decreased in thalidomide- but not placebo-treatment group (*P* < 0.05; Supplementary Fig. 2). After 12 weeks of treatment, 34 (69.4%) of patients upon thalidomide had Hb levels >70 g/L and were free of transfusion (Supplementary Table 6), including 22 (44.9%) and 10 (20.4%) patients had Hb levels ≥90 g/L and ≥110 g/L, respectively, and were completely free of transfusion, but no patients upon placebo achieved these efficacies (Fig. 2b).

### Laboratory assessments

In patients treated with thalidomide for 12 weeks, serum concentrations of lactate dehydrogenase (LDH) and α-hydroxybutyric dehydrogenase (α-HBDH), urea, uric acid (UA), glutamyl transpeptidase (GGT), and adenosine deaminase (ADA), globulin, total bilirubin (TBIL), indirect bilirubin (IBIL), and serum ferritin (SF) were decreased and albumin/globulin ratio was increased (Supplementary Table 9). In patients treated with placebo for 12 weeks, the serum concentrations of LDH, α-HBDH, TBIL, and aspartate aminotransferase (AST) were increased (Supplementary Table 9). Pharmacokinetic studies demonstrated that serum concentration of thalidomide peaked 2 h after administration and was eliminated in 24 h (Supplementary Fig. 3). The changes in serum ferritin was not significant within 12 weeks (Supplementary Table 9).

### Thalidomide induces sustainable increase of Hb concentrations in TDTs

Among the 50 patients who were randomly assigned to receive placebo, 46 ones received thalidomide in the extension phase (Fig. 1). Similar to that seen in placebo-control period, treatment with thalidomide for 12 weeks increased Hb by 10 g/L or more, or maintained Hb >70 g/L without transfusion for at least 6 weeks, in 37 (80.4%) of the 46 BTMs. On the other hand, 10 of the 15 patients who did not respond well to thalidomide in the first 12 weeks were continuously administered with the drug, and 5 of them achieved excellent or good response within additional 4 weeks. Upon thalidomide treatment for 24, 36, 48, and 60 weeks, the Hb concentrations of the patients were 104.9 ± 19.0, 108.5 ± 16.1, 106.6 ± 18.2 g/L, and 107.0 ± 19.0 g/L, respectively (Table 3 and Supplementary Fig. 4). In patients treated with thalidomide for 72, 84, and 96 weeks, the Hb concentrations were maintained at approximately 110 g/L (Table 3), without blood transfusion. Among the 90 patients treated with thalidomide for 12 weeks, excellent/good response was more frequently seen in TDTs of β0/non-β0 and non-β0/non-β0, *HBS1L-MYB* rs9399137 C/T and C/C, and *HBS1L-MYB* rs4895441 A/G and G/G genotypes (Supplementary Table 10). Thalidomide treatment prolonged the ELS from 12.8 ± 6.0 to 23.1 ± 8.5 d in 48 weeks (Supplementary Table 11), and reduced the serum ferritin from 3706.2 (1993.7, 6116.9) to 2013.5 (930.3, 4428.4) g/L in 72 weeks (Supplementary Table 12).

**Table 3:** Hb levels of the patients treated with thalidomide at indicated time points

Time	Cases, *n*	Hb (g/L), Mean ± SD
Pre-treatment	90	70.8 ± 12.8
Week 12	90	94.6 ± 17.8
Week 24	79	104.9 ± 19.0
Week 36	72	108.5 ± 16.1
Week 48	66	106.6 ± 18.2
Week 60	43	107.0 ± 19.0
Week 72	29	109.8 ± 18.4
Week 84	18	110.5 ± 17.8
Week 96	11	110.0 ± 23.7

### Quality of life and adverse effects

Thalidomide improved the Eastern Cooperative Oncology Group (ECOG) scores, in that after 12 weeks of treatment, patients upon thalidomide had lower ECOG scores (0.8 ± 0.7) than patients upon placebo (1.4 ± 0.5; *P* < 0.001; Supplementary Table 13), where higher scores indicate greater disability (ECOG score has a scale from 0 to 5; a score of 0 indicates no symptoms, and 1 indicates mild symptoms). Physical health (*P* = 0.033), insomnia (*P* = 0.033), and dyspnea (*P* = 0.054) were improved in patients taking thalidomide as reflected by the Transfusion-dependent Quality of Life (TranQoL) questionnaire and the European Organisation for Research and Treatment of Cancer (EORTC) Quality of Life Questionnaire (QLQ) scores (Supplementary Table 13).

Some mild adverse events, including drowsiness, dizziness, fatigue, pyrexia, sore throat, rash, abdominal pain, nausea, anorexia, flustered, constipation, limbs edema, and headache, were reported in patients of both groups, where grade I–II dizziness (*P* = 0.016), drowsiness (*P* = 0.057), nausea (*P* = 0.051), and rash (*P* = 0.071) were more frequently reported in patients treated with thalidomide (Supplementary Table 14). In patients treated with thalidomide for 48 weeks, no grade III–IV adverse events were reported (Supplementary Table 15).

## Discussion

BTM, an autosomal recessive disorder of Hb production, is widely distributed in Mediterranean, Southeast Asia, Africa, and countries/territories with immigrants from the above countries. BTM places a significant burden on healthcare resources and blood supplies in these countries, and tremendous efforts remain to be made to prevent unnecessary morbidity and mortality and to improve life quality of the patients. Effective and affordable therapeutics are therefore critical needs for the patients and their families as well as their communities. In this study, we showed that thalidomide was able to increase Hb levels by an average of 19.2 g/L in TDTs, with 20 (40.8%) and 14 (28.6%) of the patients achieved excellent and good response in 12 weeks of the placebo-control phase, respectively. Of note, long-term use of this drug (48–96 weeks) maintained Hb at a level of >105 g/L without transfusion. The adverse effects of thalidomide were mainly mild and tolerable, demonstrating that thalidomide could be a promising drug for TDTs.

Previous studies showed that thalidomide induces γ-globin gene expression through increased reactive oxygen species–mediated p38 mitogen-activated protein kinase (MAPK) signaling and histone H4 acetylation, and enhanced the expression of GATA binding protein 1 (GATA-1) and erythroid Krupple-like factor (EKLF) in adult erythropoiesis.^18,19^ Thalidomide promotes erythropoiesis by induction of STAT5 and GATA-1 transcription factor.^20^ Thalidomide derivatives pomalidomide and lenalidomide augment erythropoiesis, preserve bone marrow function, and reverse γ-globin silencing through transcriptional reprogramming.^21–23^ In CD34^+^ hematopoietic stem/progenitor cells from healthy individuals and patients with sickle cell anemia, lenalidomide, and pomalidomide slow down the differentiation and maturation of erythroid cells, promote the proliferation of immature red cells, regulate the transcription of Hb, and induce Hb F.^21^ Here we confirmed that thalidomide induced Hb F in β0/non-β0, non-β0/non-β0 as well as β0/β0 TDTs. Thalidomide also prolonged life span of RBCs and alleviated hemolysis reflected by decrease in TBIL, IBIL, and LDH. These results indicate that thalidomide exerts beneficial effects on BTM by regulating erythropoiesis at multiple stages, including hematopoietic stem/progenitor cells, cell differentiation, globin synthesis, and life span of RBCs. Therefore, the mechanism of action of thalidomide in treating BTM should be comprehensive, and systematic investigation should be carried out to identify the key targets/pathways that mediate the therapeutic efficacy of thalidomide in TDT.

Thalidomide was originally used as a sedative and found to be teratogenic by binding to ubiquitin E3 ligase cereblon (CRBN)^24^ and degradation of transcription factor SALL4.^25^ Thalidomide as well as derivative compounds has been used to treat other diseases, e.g., multiple myeloma^26^ by inducing degradation of C2H2 zinc finger–containing transcription factors Ikaros and Aiolos.^27–29^ Adverse events, including neurotoxicity, cardiovascular toxicology, and hepatotoxicity, have been reported in patients receiving thalidomide treatment. In this study, liver or kidney toxicity or grade III–IV events were not seen in TDT patients treated with thalidomide for up to 96 weeks. Cerebral infarction was reported in an adult patient who was shown to have developmentally abnormal right anterior and middle cerebral arteries. In 66 patients receiving thalidomide for 48 weeks, stroke was not reported. A hypercoagulable state has been documented in patients with BTM, which could result in thromboembolic events in several organs including brain.^30^ Therefore, the cause of stroke in patients upon thalidomide should be carefully scrutinized, which might not be contraindicated in patients with TDT. Since this study has several limitations, i.e., the observation period remained relatively short, the number of patients recruited was relatively small, and the dose used was relatively high, a long-term study with more enrolled patients should be performed to further determine the safety and to optimize the dose and protocol of thalidomide in BTM.

In summary, our results showed that thalidomide significantly increased Hb concentrations and reduced RBC units transfused in the 49 patients in the placebo-control period. In the extension phase of this trial, thalidomide maintained the Hb levels of the 66 patients at 106.6 ± 18.2 g/L for 48 weeks, without RBC transfusion nor grades III–IV adverse events. A phase III clinical trial is desired to further determine the safety and efficacy of thalidomide in TDT.

## Materials and methods

### Study design

This study was a multicenter, randomized, phase-II clinical trial evaluating safety and efficacy of thalidomide in patients with transfusion-dependent β-thalassemia (Chinese Clinical Trial Registry, registration number: ChiCTR1800015702). This study was approved by the Local Research Ethics Committees of the 6 participating hospital in Guangxi and Guangdong Provinces of China, and was conducted in accordance with the tenets of the Declaration of Helsinki. This study contained two phases. In the randomized, double-blind, placebo-controlled trial period, the patients were randomized 1:1 to receive placebo or thalidomide. After 12 weeks of treatment, the efficacy was evaluated. If significant efficacy was achieved in the patients, thalidomide would be continuously administered for further evaluation in the extension phase. At request of patients of control group, thalidomide was given in the extension phase after the placebo-control period (Fig. 1). The full trial protocol is available in Supplementary material.

### Patients and eligibility criteria

The diagnosis was based on clinical findings, laboratory measurements, and genotyping. The patients were residents of the resource-constrained regions of Southern China, and transfusion dependence referred to the receipt of at least eight transfusions or 100 ml per kilogram of body weight of leucoreduced packed RBCs per year, or frequent transfusion is required to maintain Hb >70 g/L in the 2 years before enrollment. Considering the available resource in the participating hospitals, the patients were transfused with packed RBCs at 0.5 U/10 kg when Hb < 70 g/L.^31^ The inclusion criteria were: TDT patients of 14 years of age or older; ECOG performance status 0–3; estimated life expectancy of at least 3 months; no bleeding tendency for 4 weeks or more; and no abnormal hemolytic factors were found. Exclusion criteria were: the use of drugs that might affect Hb levels 3 months before enrollment; deficiency in vitamin B12 and folate; having cardiopulmonary, cerebrovascular, liver, kidney or other severe diseases; breastfeeding or in childbearing age unwilling to take contraceptive measures; allergy to the drug; or currently participating in any other clinical trial.

### Randomization and treatment

Before patient recruitment began, our survey found that more than 50% of the potential participants would be willing to be enrolled in the Wuzhou Gongren Hospital. Randomization was centrally conducted by an independent statistician using RANDBETWEEN function of Microsoft Excel 2016, and sequentially numbered, opaque sealed envelopes was used to maintain allocation concealment. Eligible patients were randomly assigned to receive thalidomide or identically appearing placebo, which were orally administered per night with initial dose of 100 mg/day and escalated to 150 mg/day in 3 days if no adverse effects were reported, based on our previous studies.^13^ After 12 weeks of treatment, the efficacy was evaluated. If significant efficacy was achieved in the patients, thalidomide would be continuously administered for further evaluation in the extension phase. At request of patients of control group, thalidomide was given in the extension phase after the placebo-control period (Fig. 1).

### End points

The primary endpoint was the change of Hb level in the patients treated with thalidomide and placebo for 12 weeks. The secondary end points included the number of RBC units transfused, Hb F levels, ELS, hemolytic markers, and adverse effects. The patients were followed up every 14 days for the first 12 weeks and every 3 months thereafter. To evaluate the efficacy of the drug, response criteria were defined as follows: excellent response, an elevation in total Hb level of ≥20 g/L and the patients were free of blood transfusion for at least 6 weeks; good response, an elevation in total Hb level of 10 to 20 g/L was achieved, or Hb not substantially increased (<10 g/L) but the patients had Hb >70 g/L and were free of blood transfusion for at least 6 weeks; no response, an elevation in total Hb level of <10 g/L and the patients were still transfusion-dependent.

### Assessments

Hb level was measured every 2 weeks in the placebo-control period and every 12 weeks in the extension period. ELS was measured with an ELS assessment instrument (Seekya Biological technology Co., LTD, Shenzhen, China), and Hb F was quantified with a BioRad Variant II high-pressure liquid chromatograph (BioRad, Hercules, CA, USA), according to manufacturer’s instruction. For single-dose pharmacokinetic assessment, blood samples were collected at 0 (predose), 0.25, 0.5, 1, 2, 6, and 24 hours postdose on the day 1 and subjected to high performance liquid chromatography–mass spectrometry (Agilent Technologies, Santa Clara, CA, USA). All participants were given the self-administrated TranQol Questionnaire for Adults Version 1.0 and EORTC QLQ-C30 (V3.0) to assess the health related quality of life (HRQoL), and ECOG performance status score were collected at the beginning and the end of the trial.

### Genotyping

DNA was extracted from patients’ peripheral blood leukocytes and mutations of the β-globin gene (*HBB*) were analyzed by polymerase chain reaction (PCR)-reverse dot blot (Supplementary Table 1). Seven SNPs of *HBG2* (rs7482144),^7^ *HBS1L-MYB* (rs9399137 and rs4895441),^9^ and *BCL11A* (rs4671393, rs10189857, rs1427407 and rs11886868)^8^ were tested by Sanger sequencing of PCR products generated from DNA samples of the patients and appropriate primers (Supplementary Table 2).

### Statistical analyses

Our previous study^13^ suggested that the average elevation of Hb in thalidomide group would be at least 12 g/L higher than that in the control group after 12 weeks of treatment, and the standard deviation (SD) was presumed to be 20 g/L. Setting *α* = 0.05, two-sided test, *P* < 0.05 considered statistically significant, and supposing that 10% of the patients would withdraw or drop out, we estimated that with 80% power (1 − *β* = 0.8) approximately 100 participants were required to be enrolled in this study. Data were assessed according to intention-to-treat analysis. The primary and secondary outcomes were compared between the experimental and control groups using the Student’s *t* test at a 2-sided α level of 5%, without correction for multiple comparisons. To further compare the dynamic Hb levels and transfusion volume between the two randomized arms, we constructed a linear mixed-effects regression model in which the repeated measures were the dependent variables and intervention group was the independent variables. Data analyses were performed using IBM SPSS Statistics 26.0 (Chicago, IL, USA). All statistical tests were two-sided and *P* values less than 0.05 were considered statistically significant.

### Patient and public involvement

No patients were involved in setting the research question, or in developing plans for recruitment, design, implementation, and dissemination of the results of this study.

## Supplementary information

Supplementary materials

Protocol

## Data Availability

All data supporting this paper are present within the paper and/or the Supplementary Materials. The original data sets are also available from the corresponding author upon reasonable request.
