# Supplementary material for: Safety and efficacy of thalidomide in patients with transfusion-dependent β-thalassemia: a randomized clinical trial
Source: Signal Transduct Target Ther. 2021 Nov 18;6:405. doi: 10.1038/s41392-021-00811-0 (PMC8602273; doi:10.1038/s41392-021-00811-0)
Supplement: Supplementary file 1 — Supplementary materials [file 41392_2021_811_MOESM1_ESM.docx]

**Supplementary Materials for**

Safety and efficacy of thalidomide in patients with transfusion-dependent β-thalassemia: a randomized clinical trial

Jiang-Ming Chen^1*^, Wei-Jian Zhu^2*^, Jie Liu^3,15*^, Gui-Zhen Wang^3*^, Xiao-Qin Chen^4*^, Yun Tan^5*^, Wei-Wei Xu^1^, Li-Wei Qu^3,6^, Jin-Yan Li^1^, Huan-Ju Yang^1^, Lan Huang^1^, Ning Cai^1^, Wei-Da Wang^4^, Ken Huang^7^, Jian-Quan Xu^8^, Guo-Hui Li^9^, Sheng He^10^, Tian-Ying Luo^1^, Yi Huang^11^, Song-Hua Liu^12^, Wen-Qiang Wu^13^, Qi-Yang Lu^1^, Mei-Guang Zhou^1^, Shu-Ying Chen^1^, Rong-Lan Li^1^, Mei-Ling Hu^1^, Ying Huang^1^, Jin-Hua Wei^14^, Jun-Min Li^5^, Sai-Juan Chen^5^, Guang-Biao Zhou^3^

Correspondence to: jiangming.chen@126.com, sjchen@stn.sh.cn, gbzhou@ioz.ac.cn.

**This PDF file includes:**

**Figures S1 to S4**

**Tables S1 to S15**

**
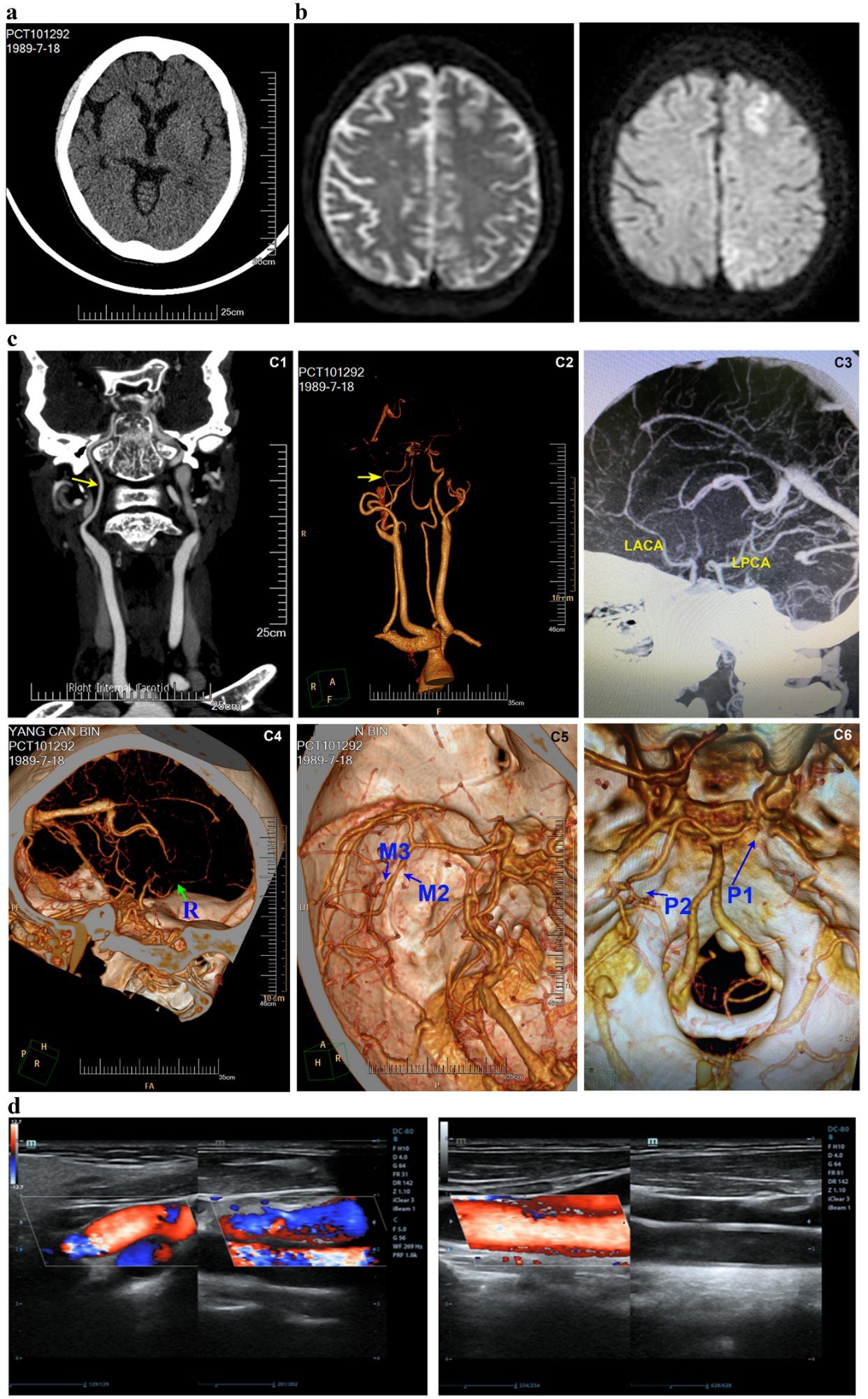
 Fig. S1. The computed tomography, computed tomography angiography, and ultrasonography assays of the patient.** (a) Computed tomography analysis of the patient. The images reveal a chronic state of vascular encephalopathy for a right insular atrophy and an atrophic dilation of the III ventricle. (b) Magnetic resonance imaging of the patient. (c) Computed tomography angiography of brain vessels. The internal carotid artery on the right side is relatively small (C1, C2; arrowed). The left anterior cerebral artery (LACA) and left posterior cerebral artery (LPCA) are detected (C3). However, neither the right anterior cerebral artery (RACA) nor the right middle cerebral artery is found (C3; C4, arrowed), suggesting that both hemispheres of the brain may use the LACA and the left middle cerebral artery for blood supply. The left middle cerebral artery exhibits mild to moderate stenosis at M2 and M3 segments, whereas the distal end is well filled (C5). The P1 segment of the right posterior cerebral artery and the P2 segment of the left posterior cerebral artery have mild to moderate stenosis, and their distal blood vessels are not well filled (C6). (d) Ultrasonography shows normal lumen, wall thickness, and blood flow velocity of internal carotid and vertebral arteries.


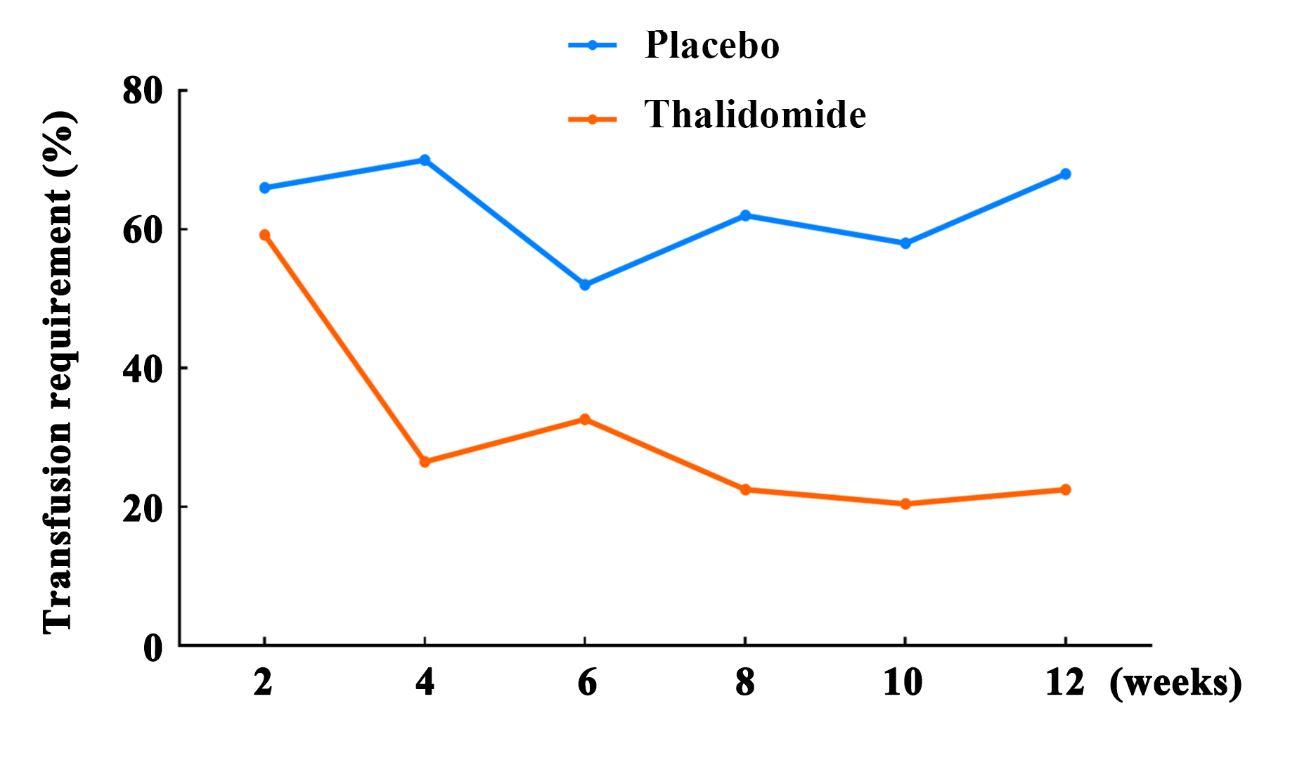


**Fig. S2. Changes in percent of blood transfusion requirement in patients upon placebo or thalidomide treatment.**

**

**

**Fig. S3. Mean plasma concentration after single dose of thalidomide.**

1. **C**

**
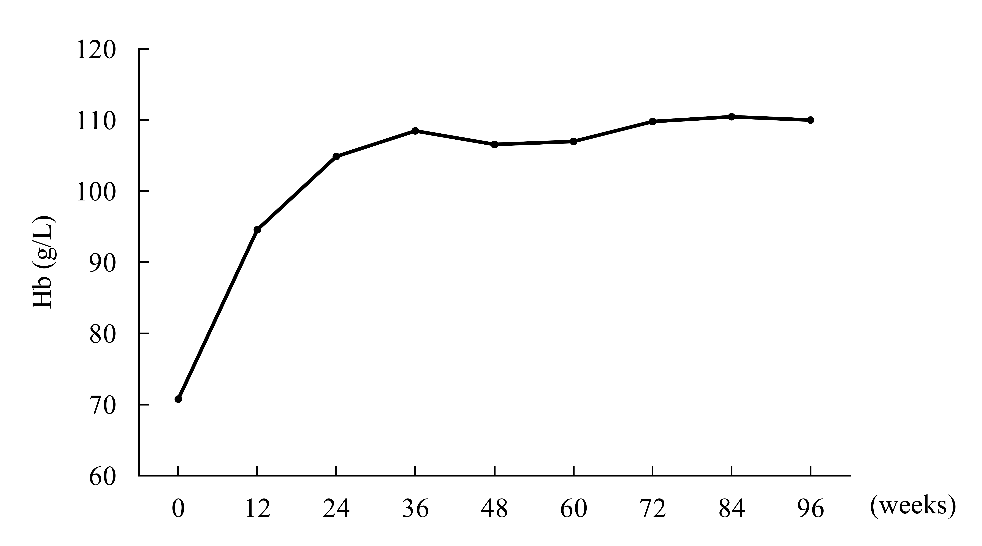
**

**Fig. S4. Hb levels of patients upon thalidomide treatment at indicated time points.**

**Table S1. Mutations of *β*-globin gene *HBB* of the patients.**

| Genotype | Types of mutation | Cases, n (%) |
| --- | --- | --- |
| β0/β0 | CD41-42(-TCTT)/CD41-42(-TCTT) | 13 |
|  | CD41-42(-TCTT)/IVS-I-1(G>T) | 2 |
|  | CD41-42(-TCTT)/CD17(A>T) | 10 |
|  | CD17(A>T)/IVS-I-1(G>T) | 1 |
|  | CD17(A>T)/CD17(A>T) | 2 |
|  | Total | 28 (28) |
| β0/non-β0 | IVS-I-1(G>T)/IVS-II-654(C>T) | 1 |
|  | CD41-42 (-TCTT)/IVS-II-654(C>T) | 5 |
|  | CD41-42 (-TCTT)/βE(G>A) | 9 |
|  | CD41-42 (-TCTT)/-28(A>G) | 23 |
|  | CD41-42 (-TCTT)/-29(A>G) | 1 |
|  | CD71-72(+A)/IVS-II-654(C>T) | 1 |
|  | CD71-72(+A)/CD26(G>A) | 1 |
|  | CD71-72(+A)/βE(G>A) | 2 |
|  | CD71-72(+A)/-28(A>G) | 2 |
|  | CD17(A>T)/IVS-II-654(C>T) | 2 |
|  | CD17(A>T)/βE(G>A) | 3 |
|  | CD17(A>T)/-28(A>G) | 7 |
|  | CD17(A>T)/-29(A>G) | 1 |
|  | CD43(G>T)/-28(A>G) | 1 |
|  | Total | 59 (59) |
| non-β0/non-β0 | IVS-II-654(C>T)/IVS-II-654(C>T) | 1 |
|  | IVS-II-654(C>T)/βE(G>A) | 1 |
|  | IVS-II-654(C>T)/-28(A>G) | 7 |
|  | IVS-II-654(C>T)/-29(A>G) | 1 |
|  | -28(A>G)/-28(A>G) | 3 |
|  | Total | 13 (13) |

**Table S2. The 7 SNPs of the patients.**

| A. The primers for detection of the SNPs. | | | | |
| --- | --- | --- | --- | --- |
| Gene | SNP | Position (bp) | Allele | Primer (5’-3’) |
| *HBG2* | rs7482144 | 5232745 | G->A | F：CCTGCACTGAAACTGTTG  R：AACTGCTGAAGGGTGCTTCC |
| *HBS1L-MYB* | rs9399137 | 135460711 | T->C | F：TGGGGTGGGAGAAGAAATAA  R：AGAAGCACTTTGGCAAGCAT |
|  | rs4895441 | 135426573 | A->G | F：GCTGGGGGAGACAAATGATA  R：GCCCTACAGGATCTCACTGC |
| *BCL11A* | rs4671393 | 60574455 | A->G | F：TAAGAGCCCCCACTAGCTCA  R：TTTAATCAGCTTCCGCCACT |
|  | rs10189857 | 60713235 | A->G | F：CCAACCTTCTGCTCTGGTTC  R：AAGTTGCCAAATTGGAGGTG |
|  | rs1427407 | 60571547 | T->G | F：ACCACGTAGTTGGGCTTCAC  R：TAGTTCCCCGTACCCATCAA |
|  | rs11886868 | 60573750 | C->T | F：CCCTTCCCTAACCCTCTGAC  R：TTGAACTCTCCAGGGAATGG |

| B. The SNPs of the patients. | | | | |
| --- | --- | --- | --- | --- |
| SNP | Total (n=100) | Placebo (n=50) | Thalidomide (n=50) | *P* |
| *HBG2* rs7482144 (G>A), n (%) | | | | |
| G/G | 75 (75) | 38 (76) | 37 (74) | 0.999 |
| A/G | 25 (25) | 12 (24) | 13 (26) |  |
| *HBS1L-MYB* rs9399137 (T>C), n (%) | | | | |
| T/T | 61 (61) | 32 (64) | 29 (58) | 0.396 |
| C/T | 35 (35) | 15 (30) | 20 (40) |  |
| C/C | 4 (4) | 3 (6) | 1 (2) |  |
| *HBS1L-MYB* rs4895441 (A>G), n (%) | | | | |
| A/A | 58 (58) | 30 (60) | 28 (56) | 0.758 |
| A/G | 39 (39) | 18 (36) | 21 (42) |  |
| G/G | 3 (3) | 2 (4) | 1 (2) |  |
| *BCL11A* rs4671393 (A>G), n (%) | | | | |
| A/A | 4 (4) | 3 (6) | 1 (2) | 0.123 |
| A/G | 46 (46) | 27 (54) | 19 (38) |  |
| G/G | 50 (50) | 20 (40) | 30 (60) |  |
| *BCL11A* rs10189857 (A>G), n (%) | | | | |
| A/A | 5 (5) | 2 (4) | 3 (6) | 0.125 |
| A/G | 40 (40) | 25 (50) | 15 (30) |  |
| G/G | 55 (55) | 23 (46) | 32 (64) |  |
| *BCL11A* rs1427407 (T>G), n (%) | | | | |
| T/T | 4 (4) | 2 (4) | 2 (4) | 0.229 |
| T/G | 41 (41) | 25 (50) | 16 (32) |  |
| G/G | 55 (55) | 23 (46) | 32 (64) |  |
| *BCL11A* rs11886868 (C>T), n (%) | | | | |
| C/C | 95 (95) | 46 (92) | 49 (98) | 0.362 |
| C/T | 5 (5) | 4 (8) | 1 (2) |  |

*P* value was determined by Fisher’s exact test.

**Table S3. Responses of the patients to the agents. The data are expressed as n (%).**

| Responses | Placebo | Thalidomide | *P* |
| --- | --- | --- | --- |
| Excellent response | 0 (0) | 20 (40.8) | 0.000 |
| Good response | 5 (10) | 14 (28.6) |  |
| No response | 45 (90) | 15 (30.6) |  |

*P* value was determined by Fisher’s exact test.

**Table S4. Genotypes and responses of the patients to thalidomide at the placebo-controlled period (n=49).**

| Genotype/SNPs | | Excellent response | Good response | No response | *P* |
| --- | --- | --- | --- | --- | --- |
| *HBB* | β0/β0, n (%) | 0 (0) | 3 (33.3) | 6 (66.7) | 0.024 |
|  | β0/non-β0, n (%) | 17 (50) | 9 (26.5) | 8 (23.5) |  |
|  | non-β0/non-β0, n (%) | 3 (50) | 2 (33.3) | 1 (16.7) |  |
| *HBG2* rs7482144 | G/G, n (%) | 14 (38.9) | 10 (27.8) | 12 (33.3) | 0.846 |
|  | A/G, n (%) | 6 (46.2) | 4 (30.8) | 3 (23.1) |  |
| *HBS1L-MYB* rs9399137 | T/T, n (%) | 9 (31.0) | 8 (27.6) | 12 (41.4) | 0.116 |
|  | C/T, n (%) | 11 (55) | 6 (30) | 3 (15) |  |
| *HBS1L-MYB* rs4895441 | A/A, n (%) | 9 (32.1) | 9 (32.1) | 10 (35.7) | 0.387 |
|  | A/G, n (%) | 11 (52.4) | 5 (23.8) | 5 (23.8) |  |
| *BCL11A* rs4671393 | A/A, n (%) | 1 (100) | 0 (0) | 0 (0) | 0.318 |
|  | A/G, n (%) | 9 (50) | 6 (33.3) | 3 (16.7) |  |
|  | G/G, n (%) | 10 (33.3) | 8 (26.7) | 12 (40) |  |
| *BCL11A* rs10189857 | A/A, n (%) | 2 (66.7) | 0 (0) | 1 (33.3) | 0.652 |
|  | A/G, n (%) | 6 (40) | 6 (40) | 3 (20) |  |
|  | G/G, n (%) | 12 (38.7) | 8 (25.8) | 11 (35.5) |  |
| *BCL11A* rs1427407 | T/T, n (%) | 1 (50) | 0 (0) | 1 (50) | 0.665 |
|  | T/G, n (%) | 7 (43.8) | 6 (37.5) | 3 (18.8) |  |
|  | G/G, n (%) | 12 (38.7) | 8 (25.8) | 11 (35.5) |  |
| *BCL11A* rs11886868 | C/C, n (%) | 19 (39.6) | 14 (29.2) | 15 (31.3) | 0.999 |
|  | C/T, n (%) | 1 (100) | 0 (0) | 0 (0) |  |

*P* values were determined by Fisher’s exact test.

**Table S5. Hb, red blood cells (RBCs) and platelets (PLT) of the patient who received splenectomy or not.**

| A. Blood components of patients received splenectomy or not. | | | | | |
| --- | --- | --- | --- | --- | --- |
| Splenectomy | Indicator | Time | Placebo | Thalidomide | *P* |
| Yes | Hb (g/L) | Week 0 | 79.69 ± 14.71 | 83.31 ± 12.71 | 0.455^*^ |
|  |  | Week 2 | 74.46 ± 10.20 | 83.38 ± 8.37 | 0.010^*^ |
|  |  | Week 4 | 73.08 ± 11.08 | 89.23 ± 12.36 | 0.000^*^ |
|  |  | Week 6 | 72.15 ± 10.05 | 90.77 ± 14.80 | 0.000^*^ |
|  |  | Week 8 | 74.54 ± 9.21 | 89.08 ± 14.19 | 0.000^*^ |
|  |  | Week 10 | 72.12 ± 8.87 | 92.38 ± 19.44 | 0.003^*^ |
|  |  | Week 12 | 73.23 ± 9.75 | 92.92 ± 18.75 | 0.003^*^ |
| Yes | RBC (×10^9^/L) | Week 0 | 3.2 (2.8, 3.5) | 3.4 (3.3, 3.7) | 0.063^#^ |
|  |  | Week 2 | 3.1 (2.7, 3.3) | 3.7 (3.2, 4.0) | 0.001^#^ |
|  |  | Week 4 | 3.1 (2.6, 3.3) | 4.0 (3.8, 4.5) | 0.000^#^ |
|  |  | Week 6 | 3.0 (2.6, 3.3) | 4.3 (4.0, 4.6) | 0.000^#^ |
|  |  | Week 8 | 3.0 (2.7, 3.3) | 4.4 (3.6, 4.8) | 0.000^#^ |
|  |  | Week 10 | 3.1 (2.7, 3.2) | 4.4 (3.9, 4.9) | 0.000^#^ |
|  |  | Week 12 | 3.0 (2.7, 3.3) | 4.4 (3.7, 5.0) | 0.000^#^ |
| Yes | PLT (×10^9^/L) | Week 0 | 726 (583, 891) | 716 (587, 893) | 0.835^#^ |
|  |  | Week 2 | 668 (542, 903) | 731 (534, 892) | 0.812^#^ |
|  |  | Week 4 | 670 (552, 857) | 789 (635, 983) | 0.190^#^ |
|  |  | Week 6 | 741 (585, 864) | 828 (579, 1068) | 0.571^#^ |
|  |  | Week 8 | 688 (563, 938) | 729 (586, 911) | 0.688^#^ |
|  |  | Week 10 | 787 (638, 1056) | 687 (534, 916) | 0.326^#^ |
|  |  | Week 12 | 672 (531, 871) | 676 (531, 917) | 0.988^#^ |
| No | Hb (g/L) | Week 0 | 71.42 ± 14.08 | 68.44 ± 15.57 | 0.455^*^ |
|  |  | Week 2 | 66.79 ± 11.12 | 71.31 ± 8.46 | 0.080^*^ |
|  |  | Week 4 | 65.29 ± 9.65 | 81.31 ± 12.87 | 0.000^*^ |
|  |  | Week 6 | 70.54 ± 6.90 | 83.03 ±14.08 | 0.000^*^ |
|  |  | Week 8 | 67.54 ± 12.18 | 86.33 ± 15.94 | 0.000^*^ |
|  |  | Week 10 | 67.91 ± 9.70 | 88.39 ± 17.38 | 0.000^*^ |
|  |  | Week 12 | 68.33 ± 8.18 | 91.11 ± 17.11 | 0.000^*^ |
| No | RBC (×10^9^/L) | Week 0 | 3.0 (2.5, 3.2) | 2.8 (2.5, 3.2) | 0.803^#^ |
|  |  | Week 2 | 2.7 (2.3, 3.3) | 2.9 (2.5, 3.5) | 0.139^#^ |
|  |  | Week 4 | 2.6 (2.4, 3.0) | 3.5 (2.8, 4.3) | 0.000^#^ |
|  |  | Week 6 | 2.8 (2.6, 3.3) | 3.8 (2.9, 4.4) | 0.000^#^ |
|  |  | Week 8 | 2.7 (2.5, 3.1) | 4.0 (3.1, 4.9) | 0.000^#^ |
|  |  | Week 10 | 2.8 (2.4, 3.1) | 4.3 (3.2, 5.0) | 0.000^#^ |
|  |  | Week 12 | 2.7 (2.5, 3.1) | 4.8 (3.3, 5.0) | 0.000^#^ |
| No | PLT (×10^9^/L) | Week 0 | 172 (105, 251) | 168 (131, 240) | 0.856^#^ |
|  |  | Week 2 | 165 (107, 218) | 195 (139, 256) | 0.079^#^ |
|  |  | Week 4 | 150 (102, 256) | 186 (153, 258) | 0.210^#^ |
|  |  | Week 6 | 172 (122, 261) | 201 (168, 281) | 0.167^#^ |
|  |  | Week 8 | 158 (112, 244) | 216 (179, 256) | 0.019^#^ |
|  |  | Week 10 | 160 (103, 285) | 226 (165, 272) | 0.071^#^ |
|  |  | Week 12 | 162 (112, 235) | 203 (157, 282) | 0.105^#^ |

B. Blood components in patients received placebo or thalidomide.

| Group | Indicator | Time | Splenectomy | Non-splenectomy | *P* |
| --- | --- | --- | --- | --- | --- |
| Placebo | Hb (g/L) | Week 0 | 79.69 ± 14.71 | 71.42 ± 14.08 | 0.048^*^ |
|  |  | Week 2 | 74.46 ± 10.20 | 66.79 ± 11.12 | 0.014^*^ |
|  |  | Week 4 | 73.08 ± 11.08 | 65.29 ± 9.65 | 0.011^*^ |
|  |  | Week 6 | 72.15 ± 10.05 | 70.54 ± 6.90 | 0.516^*^ |
|  |  | Week 8 | 74.54 ± 9.21 | 67.54 ± 12.18 | 0.026^*^ |
|  |  | Week 10 | 72.11 ± 8.87 | 67.91 ± 9.70 | 0.116^*^ |
|  |  | Week 12 | 73.23 ± 9.75 | 68.33 ± 8.18 | 0.061^*^ |
| Placebo | RBC (×10^9^/L) | Week 0 | 3.2 (2.8, 3.5) | 3.0 (2.5, 3.2) | 0.026^#^ |
|  |  | Week 2 | 3.1 (2.7, 3.3) | 2.7 (2.3, 3.3) | 0.072^#^ |
|  |  | Week 4 | 3.1 (2.6, 3.3) | 2.6 (2.4, 3.0) | 0.023^#^ |
|  |  | Week 6 | 3.0 (2.6, 3.3) | 2.8 (2.6, 3.3) | 0.838^#^ |
|  |  | Week 8 | 3.0 (2.7, 3.3) | 2.7 (2.5, 3.1) | 0.052^#^ |
|  |  | Week 10 | 3.1 (2.7, 3.2) | 2.8 (2.4, 3.1) | 0.268^#^ |
|  |  | Week 12 | 3.0 (2,7, 3.3) | 2.7 (2.5, 3.1) | 0.041^#^ |
| Placebo | PLT (×10^9^/L) | Week 0 | 726 (583, 891) | 172 (105, 251) | 0.000^#^ |
|  |  | Week 2 | 668 (542, 903) | 165 (107, 218) | 0.000^#^ |
|  |  | Week 4 | 670 (552, 857) | 150 (102, 256) | 0.000^#^ |
|  |  | Week 6 | 741 (585, 864) | 172 (122, 261) | 0.000^#^ |
|  |  | Week 8 | 688 (563, 938) | 158 (112, 244) | 0.000^#^ |
|  |  | Week 10 | 787 (638, 1056) | 160 (103, 285) | 0.000^#^ |
|  |  | Week 12 | 672 (531, 871) | 162 (112, 235) | 0.000^#^ |
| Thalidomide | Hb (g/L) | Week 0 | 83.31 ± 12.71 | 68.44 ± 15.57 | 0.003^*^ |
|  |  | Week 2 | 83.38 ± 8.37 | 71.31 ± 8.46 | 0.000^*^ |
|  |  | Week 4 | 89.23 ± 12.36 | 81.31 ± 12.87 | 0.061^*^ |
|  |  | Week 6 | 90.77 ± 14.80 | 83.03 ± 14.08 | 0.100^*^ |
|  |  | Week 8 | 89.08 ± 14.19 | 86.33 ± 15.94 | 0.587^*^ |
|  |  | Week 10 | 92.38 ± 19.44 | 88.39 ± 17.38 | 0.494^*^ |
|  |  | Week 12 | 92.92 ± 18.75 | 91.11 ± 17.11 | 0.751^*^ |
| Thalidomide | RBC (×10^9^/L) | Week 0 | 3.4 (3.3, 3.7) | 2.8 (2.5, 3.2) | 0.000^#^ |
|  |  | Week 2 | 3.7 (3.2, 4.0) | 2.9 (2.5, 3.5) | 0.002^#^ |
|  |  | Week 4 | 4.0 (3.8, 4.5) | 3.5 (2.8, 4.3) | 0.067^#^ |
|  |  | Week 6 | 4.3 (4.0, 4.6) | 3.8 (2.9, 4.4) | 0.063^#^ |
|  |  | Week 8 | 4.4 (3.6, 4.8) | 4.0 (3.1, 4.9) | 0.428^#^ |
|  |  | Week 10 | 4.4 (3.9, 4.9) | 4.3 (3.2, 5.0) | 0.435^#^ |
|  |  | Week 12 | 4.4 (3.7, 5.0) | 4.8 (3.3, 5.0) | 0.659^#^ |
| Thalidomide | PLT (×10^9^/L) | Week 0 | 716 (587, 893) | 168 (131, 240) | 0.000^#^ |
|  |  | Week 2 | 731 (534, 891) | 195 (139, 256) | 0.000^#^ |
|  |  | Week 4 | 789 (635, 983) | 186 (153, 258) | 0.000^#^ |
|  |  | Week 6 | 828 (579, 1068) | 201 (168, 281) | 0.000^#^ |
|  |  | Week 8 | 729 (586, 911) | 216 (179, 256) | 0.000^#^ |
|  |  | Week 10 | 687 (534, 916) | 226 (165, 272) | 0.000^#^ |
|  |  | Week 12 | 676 (531, 917) | 203 (157, 282) | 0.000^#^ |

*P* value was determined by Student's *t* test (*) or Mann-Whitney U test (#).

**Table S6. Changes of Hb F, ELS and transfusion requirement in the patients treated with thalidomide or placebo. Data are expressed as median (range) or mean ± SD.**

|  | Placebo | Thalidomide | *P* |
| --- | --- | --- | --- |
| Hb F at week 12, % | 21.9 (6.6, 48.3) | 55.2 (35.7, 79.1) | 0.000^#^ |
| Change in Hb F, % | 2.4 (-0.8, 10.2) | 34.8 (17.8, 51.0) | 0.000^#^ |
| ELS at week 12, d | 12.8 ± 5.0 | 16.4 ± 6.6 | 0.003^*^ |
| Changes in ELS, d | -0.9 ± 5.0 | 3.0 ± 7.5 | 0.004^*^ |
| Mean transfusion volume, U | 10.3 ± 6.4 | 5.4 ± 5.0 | 0.000^*^ |
| Hb > 70 g/L without transfusion, n (%) | 5 (10) | 34 (69.4) | 0.000^$^ |

Hb F, fetal hemoglobin; Change in Hb F, Hb F_d85_-Hb F_d0_; ELS, erythrocyte life span; RBC, red blood cell. *P* values were determined by Student's *t* test (*), Fisher’s exact test ($), or Mann-Whitney U test (#).

**Table S7. Change of Hb components in the patients treated with thalidomide or placebo. The data are expressed as median (range).**

| Genotype | Hb A_d85_-Hb A_d0_ (%) | | | Hb F_d85_-Hb F_d0_ (%) | | | Hb A2_d85_-Hb A2_d0_ (%) | | |
| --- | --- | --- | --- | --- | --- | --- | --- | --- | --- |
|  | Placebo | Thalidomide | *P* | Placebo | Thalidomide | *P* | Placebo | Thalidomide | *P* |
| All patients | -2.8 (-15.6, 2.3) | -37.0 (-53.4, 11.2) | 0.000 | 2.4 (-0.8, 10.2) | 34.8 (17.8, 51.0) | 0.000 | 0.1 (-0.3, 0.5) | -0.8 (-1.2, -0.1) | 0.000 |
| Genotype |  |  |  |  |  |  |  |  |  |
| β0/β0 | -2.6 (-11.3, 0.6) | -25.9 (-57.3, -16.2) | 0.004 | 2.0 (-0.3, 6.6) | 26.0 (18.3, 67.8) | 0.000 | 0.1 (-0.2, 0.3) | -0.3 (-1.0, -0.1) | 0.009 |
| β0/non-β0 | -6.7 (-23.9, 6.5) | -37.0 (-53.4, -6.3) | 0.002 | 4.6 (-1.5, 15.1) | 32.2 (16.3, 50.9) | 0.000 | 0.1 (-0.3, 0.7 ) | -0.8 (-1.3, -0.2) | 0.000 |
| non-β0/non-β0 | 5.8 (-1.6, 14.2) | -40 | 0.157 | -3.4 (-10.5, 2.7) | 39.3 (26.4, 45.6) | 0.004 | -0.2 (-1.0, 0.2) | -0.3 (-1.1, 0.2) | 0.810 |

*P* values were determined by Mann-Whitney U test.

**Table S8. Changes of WBCs and PLT in the patients treated with thalidomide or placebo. The data are expressed as median (range).**

| Genotype | WBC_d85_-WBC_d0_ (×10^9^/L) | | | Plt_d85_-Plt_d0_ (×10^9^/L) | | |
| --- | --- | --- | --- | --- | --- | --- |
|  | Placebo | Thalidomide | *P* | Placebo | Thalidomide | *P* |
| All patients | 0.2 (-0.8, 1.7) | -0.3 (-1.7, 1.0) | 0.136 | 1.0 (-59.0, 46.5) | 22.0 (-45.0, 72.5) | 0.147 |
| Genotype |  |  |  |  |  |  |
| β0/β0 | -0.1 (-0.9, 0.9) | -0.6 (-1.5, 0.8) | 0.539 | 15.0 (-50.0, 61.0) | 33.0 (4.5, 83.0) | 0.446 |
| β0/non-β0 | 0.4 (-0.6, 2.1) | -0.1 (-2.0, 1.2) | 0.075 | -4.0 (-88.5, 22.0) | 25.0 (-56.8, 71.8) | 0.123 |
| non-β0/non-β0 | -0.0 ( -2.5, 0.5) | 0.1 (-2.3, 0.7) | 0.688 | 10.0 (-56.8, 57.8) | 0.0 (-48.0, 102.8) | 0.873 |

*P* values were determined by Mann-Whitney U test.

**Table S9. Biochemical parameters of the patients treated with placebo or thalidomide.**

| Parameters | Placebo | Thalidomide | *P* |
| --- | --- | --- | --- |
| Cardiac function (4 items) | | | |
| Creatine kinase (CK; U/L) | | | |
| Week 0 | 27.9 (20.5, 35.9) | 28.6 (17.0, 39.1) | 0.352 |
| Week 12 | 23.8 (17.4, 34.2) | 21.0 (16.3, 29.0) | 0.200 |
| Change | -3.8 (-7.6, -1.0) | -2.2 (-13.4, 3.0) | 0.491 |
| Creatine kinase isoenzyme MB (CK-MB; U/L) | | | |
| Week 0 | 12.4 (7.4, 17.8) | 10.5 (6.7, 14.0) | 0.104 |
| Week 12 | 12.7 (6.0, 15.6) | 12.0 (9.0, 14.7) | 0.527 |
| Change | 0.0 (-6.8, 2.0) | 1.8 (-3.0, 6.7) | 0.076 |
| Lactate dehydrogenase (LDH; U/L) | | | |
| Week 0 | 187.5 (159.0, 257.5) | 210.50 (140.0, 312.6) | 0.602 |
| Week 12 | 251.0 (199.3, 327.8) | 186.50 (147.3, 304.3) | 0.011 |
| Change | 58.0 (-6.0, 109.9) | 0.6 (-143.9, 58.0) | 0.002 |
| α-Hydroxybutyric dehydrogenase (α-HBDH; U/L) | | | |
| Week 0 | 163.5 (137.8, 258.3) | 189.0 (104.8, 289.3) | 0.796 |
| Week 12 | 213.9 (159.6, 273.1) | 147.0 (97.7, 237.8) | 0.011 |
| Change | 54.2 (-22.9, 116.1) | -16.1 (-135.6, 28.6) | 0.007 |
| Renal function (7 items) | | | |
| Urea (mmol/L) | | | |
| Week 0 | 4.3 (3.8, 5.4) | 4.5 (3.8, 5.3) | 0.867 |
| Week 12 | 4.5 (3.9, 5.6) | 3.6 (3.0, 4.7) | 0.003 |
| Change | 0.0 (-0.6, 0.9) | -0.4 (-1.4, 0.6) | 0.028 |
| Creatinine (Cre; μmol/L) | | | |
| Week 0 | 38.0 (32.0, 47.3) | 38.0 (35.0, 47.2) | 0.488 |
| Week 12 | 38.0 (31.5, 46.0) | 37.1 (30.0, 49.0) | 0.786 |
| Change | 2.0 (-4.5, 6.6) | -2.0 (-7.0, 3.0) | 0.060 |
| Uric acid (UA; μmol/L) | | | |
| Week 0 | 417.0 (347.9, 502.7) | 431.9 (308.3, 511.0) | 0.715 |
| Week 12 | 428.8 (353.2, 551.0) | 331.7 (250.5, 420.5) | 0.000 |
| Change | 19.2 (-50.7, 69.3) | -66.3 (-138.8, -15.5) | 0.000 |
| Cystatin c (CYS-C; mg/L) | | | |
| Week 0 | 0.8 (0.7, 1.0) | 0.8 (0.7, 0.9) | 0.824 |
| Week 12 | 0.8 (0.7, 1.0) | 0.8 (0.6, 0.9) | 0.292 |
| Change | 0.0 (-0.1, 0.1) | -0.0 (-0.1, 0.1) | 0.180 |
| Endogenous creatinine clearance rate (CCr; mL/min/m2) | | | |
| Week 0 | 93.7 (76.0, 110.5) | 94.9 (81.2, 106.7) | 0.750 |
| Week 12 | 92.5 (76.8, 108.9) | 92.5 (76.8, 108.5) | 0.532 |
| Change | -5.3 (-15.5, 6.4) | 1.9 (-18.3, 11.7) | 0.404 |
| β_2_-Microglobulin (β_2_-MG; mg/L) | | | |
| Week 0 | 2.5 (2.2, 3.2) | 2.5 (2.0, 3.2) | 0.708 |
| Week 12 | 2.6 (2.2, 3.1) | 2.6 (2.1, 3.1) | 0.800 |
| Change | 0.1 (-0.4, 0.4) | 0.1 (-0.3, 0.4) | 0.625 |
| Carbon dioxide combining power (CO2-cp; mmol/L) | | | |
| Week 0 | 23.7 (21.9, 26.1) | 24.1 (22.4, 26.2) | 0.679 |
| Week 12 | 24.5 (23.1, 26.5) | 24.3 (22.6, 26.6) | 0.970 |
| Change | 1.1 (-1.7, 2.9) | 0.5 (-1.9, 2.5) | 0.529 |
| Hepatic function (17 items) | | | |
| Total protein (TP; g/L) | | | |
| Week 0 | 76.0 (68.1, 80.8) | 74.4 (67.5, 81.0) | 0.733 |
| Week 12 | 75.0 (70.4, 81.0) | 71.5 (64.6, 77.0) | 0.015 |
| Change | 0.4 (-3.9, 6.6) | -3.3 (-6.4, -0.4) | 0.001 |
| Albumin (ALB; g/L) | | | |
| Week 0 | 42.7 (41.1, 46.1) | 44.0 (40.7, 46.0) | 0.596 |
| Week 12 | 43.1 (41.0, 45.8) | 43.4 (39.9, 46.6) | 0.963 |
| Change | 0.8 (-1.8, 2.2) | 0.0 (-2.3, 2.5) | 0.410 |
| Globulin (GLB; g/L) | | | |
| Week 0 | 32.3 (25.6, 37.8) | 29.8 (23.5, 38.8) | 0.509 |
| Week 12 | 31.8 (26.4, 37.3) | 27.0 (21.1, 35.6) | 0.029 |
| Change | 0.0 (-2.9, 4.3) | -2.9 (-6.4, -0.5) | 0.003 |
| Albumin/globulin ration (A/G) | | | |
| Week 0 | 1.3 (1.1, 1.8) | 1.6 (1.1, 1.8) | 0.394 |
| Week 12 | 1.4 (1.1, 1.7) | 1.7 (1.1, 2.1) | 0.059 |
| Change | 0.0 (-0.2, 0.2) | 0.1 (-0.0, 0.3) | 0.022 |
| Direct bilirubin (DBIL; μmol/L) | | | |
| Week 0 | 10.2 (7.5, 12.2) | 10.3 (7.2, 13.4) | 0.826 |
| Week 12 | 10.3 (8.3, 13.3) | 11.0 (8.4, 16.6) | 0.361 |
| Change | 0.8 (-1.5, 2.6) | 1.2 (-1.9, 4.3) | 0.324 |
| Indirect bilirubin (IBIL; μmol/L) | | | |
| Week 0 | 33.2 (22.8, 45.3) | 35.6 (25.3, 48.7) | 0.612 |
| Week 12 | 36.7 (27.3, 52.6) | 33.4 (22.9, 47.5) | 0.264 |
| Change | 5.0 (-1.0, 10.7) | -1.2 (-9.9, 6.0) | 0.005 |
| Total bilirubin (TBIL; μmol/L) | | | |
| Week 0 | 42.6 (32.0, 57.9) | 48.4 (32.9, 63.2) | 0.592 |
| Week 12 | 48.4 (36.9, 67.5) | 47.5 (32.2, 61.5) | 0.365 |
| Change | 6.1 (-1.0, 13.0) | -0.8 (-11.7, 8.0) | 0.013 |
| Aspartate aminotransferase (AST; U/L) | | | |
| Week 0 | 39.3 (26.2, 63.5) | 30.7 (22.5, 45.7) | 0.105 |
| Week 12 | 43.8 (26.0, 67.8) | 28.8 (20.9, 47.1) | 0.008 |
| Change | 4.0 (-4.7, 7.8) | 0.0 (-10.8, 6.5) | 0.107 |
| Alanine transaminase (ALT; U/L) | | | |
| Week 0 | 33.0 (23.1, 65.8) | 30.9 (17.3, 58.7) | 0.310 |
| Week 12 | 36.7 (21.8, 62.0) | 35.6 (20.3, 70.0) | 0.958 |
| Change | -0.2 (-8.5, 7.0) | 4.9 (-7.7, 15.3) | 0.153 |
| AST/ALT | | | |
| Week 0 | 1.1 (0.9, 1.5) | 1.1 (0.8, 1.5) | 0.637 |
| Week 12 | 1.2 (0.9, 1.5) | 0.9 (0.7, 1.2) | 0.001 |
| Change | 0.1 (-0.1, 0.2) | -0.2 (-0.4, -0.0) | 0.000 |
| Glutamyl transpeptidase (GGT; U/L) | | | |
| Week 0 | 22.6 (16.0, 43.9) | 20.6 (13.9, 25.9) | 0.058 |
| Week 12 | 24.5 (18.0, 48.3) | 8.6 (6.6, 13.4) | 0.000 |
| Change | 1.0 (-5.4, 4.6) | -10.8 (-15.6, -6.0) | 0.000 |
| Alkaline phosphatase (ALP; U/L) | | | |
| Week 0 | 178.6 (130.9, 253.2) | 146.3 (105.4, 186.8) | 0.014 |
| Week 12 | 173.5 (114.8, 208.8) | 124.0 (85.5, 165.9) | 0.002 |
| Change | -20.1 (-49.9, 4.2) | -24.3 (-50.8, 7.7) | 0.969 |
| Total bile acid (TBA; μmol/L) | | | |
| Week 0 | 7.4 (4.3, 12.2) | 8.9 (5.4, 13.5) | 0.274 |
| Week 12 | 7.1 (4.0, 11.5) | 6.3 (3.6, 12.0) | 0.569 |
| Change | 0.3 (-3.1, 3.8) | -2.2 (-5.1, 0.8) | 0.040 |
| Cholinesterase (CHE; U/L) | | | |
| Week 0 | 6185.5 (5248.3, 7091.8) | 5708.0 (4851.5, 7031.3) | 0.541 |
| Week 12 | 6042.0 (5051.8, 6584.8) | 6086.0 (4910.8, 6850.8) | 0.906 |
| Change | -130.0 (-632.0, 321.3) | 203.5 (-559.3, 809.0) | 0.184 |
| 5’-NT nuclease (5’-NT; U/L) | | | |
| Week 0 | 3.8 (2.5, 5.6) | 3.1 (2.4, 4.3) | 0.176 |
| Week 12 | 3.5 (2.5, 5.1) | 2.7 (1.9, 3.9) | 0.009 |
| Change | -0.0 (-1.2, 0.8) | -0.5 (-1.0, 0.3) | 0.186 |
| Adenosine deaminase (ADA; U/L) | | | |
| Week 0 | 26.8 (20.9, 32.6) | 20.5 (16.3, 28.9) | 0.010 |
| Week 12 | 26.9 (20.0, 34.4) | 17.2 (14.3, 22.1) | 0.000 |
| Change | -1.1 (-4.1, 2.9) | -3.1 (-8.3, 0.4 ) | 0.023 |
| Prealbumin (PA; mg/dL) | | | |
| Week 0 | 21.1 (18.4, 26.2) | 22.3 (18.2, 24.7) | 0.829 |
| Week 12 | 21.8 (16.9, 26.4) | 19.9 (17.6, 22.5) | 0.117 |
| Change | 0.5 (-3.8, 2.3) | -2.5 (-5.1, 1.7) | 0.083 |
| Ferritin (ng/mL) |  |  |  |
| Week 0 | 5316.2 (3466.6, 7409.1) | 2937.8 (1254.3, 5278.9) | 0.004 |
| Week12 | 5162.9 (2779.5, 6936.7) | 2732.3 (1758.5, 5011.8) | 0.002 |
| Change | -692.4 (-1757.6, 655.0) | -263.9 (-835.8, 595.2) | 0.343 |

*P* values were determined by Mann-Whitney U test.

**Table S10. Genotypes and responses of the patients to thalidomide in the extension phase (n=90).**

| Genotype/SNP | | Excellent response | Good response | No response | *P* |
| --- | --- | --- | --- | --- | --- |
| *HBB* | β0/β0, n (%) | 9 (37.5) | 7 (29.2) | 8 (33.3) | 0.073 |
|  | β0/non-β0, n (%) | 35 (64.8) | 14 (25.9) | 5 (9.3) |  |
|  | non-β0/non-β0, n (%) | 7 (58.3) | 4 (33.3) | 1 (8.3) |  |
| *HBG2* rs7482144 | G/G, n (%) | 35 (53.0) | 19 (28.8) | 12 (18.2) | 0.454 |
|  | A/G, n (%) | 16 (66.7) | 6 (25) | 2 (8.3) |  |
| *HBS1L-MYB* rs9399137 | T/T, n (%) | 23 (42.6) | 17 (31.5) | 14 (25.9) | 0.001 |
|  | C/T, n (%) | 25 (75.8) | 8 (24.2) | 0 (0) |  |
|  | C/C, n (%) | 3 (100) | 0 (0) | 0 (0) |  |
| *HBS1L-MYB* rs4895441 | A/A, n (%) | 21 (41.2) | 18 (35.3) | 12 (23.5) | 0.007 |
|  | A/G, n (%) | 28 (75.7) | 7 (18.9) | 2 (5.4) |  |
|  | G/G, n (%) | 2 (100) | 0 (0) | 0 (0) |  |
| *BCL11A* rs4671393 | A/A, n (%) | 3 (75) | 0 (0) | 1 (25) | 0.277 |
|  | A/G, n (%) | 27 (64.3) | 11 (26.2) | 4 (9.5) |  |
|  | G/G, n (%) | 21 (47.7) | 14 (31.8) | 9 (20.5) |  |
| *BCL11A* rs10189857 | A/A, n (%) | 5 (100) | 0 (0) | 0 (0) | 0.386 |
|  | A/G, n (%) | 20 (57.1) | 11 (31.4) | 4 (11.4) |  |
|  | G/G, n (%) | 26 (52) | 14 (28) | 10 (20) |  |
| *BCL11A* rs1427407 | T/T, n (%) | 4 (100) | 0 (0) | 0 (0) | 0.498 |
|  | T/G, n (%) | 21 (58.3) | 11 (30.6) | 4 (11.1) |  |
|  | G/G, n (%) | 26 (52) | 14 (28) | 10 (20) |  |
| *BCL11A* rs11886868 | C/C, n (%) | 49 (57.6) | 24 (28.2) | 12 (14.1) | 0.282 |
|  | C/T, n (%) | 2 (40) | 1 (20) | 2 (40) |  |

*P* value was determined by Fisher’s exact test.

**Table S11. ELS of the patients treated with thalidomide for 48 weeks.**

| Time | Cases, n | ELS (d), Mean ± SD |
| --- | --- | --- |
| Pre-treatment | 90 | 12.8 ± 6.0 |
| 12 weeks | 76 | 17.4 ± 7.1 |
| 24 weeks | 63 | 21.2 ± 8.0 |
| 48 weeks | 52 | 23.1 ± 8.5 |

**Table S12. Ferritin of the patients treated with thalidomide for 96 weeks. The data are expressed as median (range).**

| Time | Cases, n | Ferritin (ng/mL) |
| --- | --- | --- |
| pre-treatment | 90 | 3706.2 (1993.7, 6116.9) |
| 12 weeks | 71 | 3301.4 (1992.8, 6126.8) |
| 24 weeks | 48 | 4017.8 (2717.5, 6677.0) |
| 36 weeks | 13 | 3019.6 (849.7, 6296.9) |
| 48 weeks | 54 | 3107.3 (1584.4, 5192.8) |
| 60 weeks | 10 | 2176.6 (1016.9, 3285.3) |
| 72 weeks | 12 | 2013.5 (930.3, 4428.4) |
| 84 weeks | 5 | 2619.4 (1089.1, 4010.8) |
| 96 weeks | 8 | 1939.3 (1287.2, 3024.2) |

**Table S13. Quality of life reflected by questionnaire and performance status scores.**

| Indicator | Placebo | Thalidomide | *P* |
| --- | --- | --- | --- |
| ECOG performance status | | | |
| ECOG score | | | |
| Week 0 | 1.6 ± 0.6 | 1.6 ± 0.6 | 0.819 |
| Week 12 | 1.4 ± 0.5 | 0.8 ± 0.7 | 0.000 |
| TranQoL questionnaire | | | |
| Summary scores | | | |
| Week 0 | 58.4 ± 13.1 | 59.3 ± 13.4 | 0.733 |
| Week 12 | 59.3 ± 15.1 | 63.4 ± 12.6 | 0.149 |
| Physical health | | | |
| Week 0 | 57.2 ± 12.7 | 57.7 ± 14.2 | 0.868 |
| Week 12 | 56.8 ± 14.4 | 63.2 ± 15.1 | 0.033 |
| Emotional health | | | |
| Week 0 | 64.3 ± 16.4 | 63.3 ± 16.2 | 0.763 |
| Week 12 | 64.9 ± 17.3 | 66.9 ± 15.0 | 0.523 |
| Family health | | | |
| Week 0 | 51.4 ± 19.2 | 54.4 ± 17.9 | 0.426 |
| Week 12 | 54.3 ± 20.1 | 58.4 ± 15.6 | 0.262 |
| School and career health | | | |
| Week 0 | 50.5 ± 26.0 | 53.5 ± 27.6 | 0.573 |
| Week 12 | 51.6 ± 27.8 | 58.0 ± 23.0 | 0.218 |
| QLQ-C30 questionnaire | | | |
| Physical functioning (PF) | | | |
| Week 0 | 80.1 ± 16.3 | 80.7 ± 15.1 | 0.862 |
| Week 12 | 81.1 ± 16.6 | 85.6 ± 11.0 | 0.123 |
| Role functioning (RF) | | | |
| Week 0 | 83.3 ± 23.3 | 86.8 ± 17.2 | 0.407 |
| Week 12 | 83.7 ± 24.6 | 89.2 ± 19.9 | 0.225 |
| Emotional functioning (EF) | | | |
| Week 0 | 81.3 ± 18.2 | 82.8 ± 16.8 | 0.670 |
| Week 12 | 82.1 ± 20.4 | 85.2 ± 14.1 | 0.387 |
| Cognitive functioning (CF) | | | |
| Week 0 | 90.1 ± 15.6 | 88.5 ± 14.2 | 0.600 |
| Week 12 | 84.4 ± 20.0 | 89.2 ± 14.0 | 0.167 |
| Social functioning (SF) | | | |
| Week 0 | 69.4 ± 27.3 | 65.6 ± 26.9 | 0.496 |
| Week 12 | 67.3 ± 29.7 | 74.7 ± 25.5 | 0.196 |
| Global health (QL) | | | |
| Week 0 | 67.9 ± 21.2 | 64.2 ± 17.8 | 0.365 |
| Week 12 | 62.9 ± 23.9 | 67.5 ± 18.8 | 0.295 |
| Fatigue (FA) | | | |
| Week 0 | 32.2 ± 18.2 | 35.2 ± 19.6 | 0.439 |
| Week 12 | 30.6 ± 18.8 | 27.5 ± 21.4 | 0.455 |
| Nausea and vomiting (NV) | | | |
| Week 0 | 2.0 ± 6.5 | 3.5 ± 9.7 | 0.394 |
| Week 12 | 3.1 ± 8.8 | 3.1 ± 7.4 | 0.969 |
| Pain (PA) | | | |
| Week 0 | 14.6 ± 19.1 | 13.9 ± 17.3 | 0.843 |
| Week 12 | 14.3 ± 20.1 | 11.5 ± 18.2 | 0.470 |
| Dyspnoea (DY) | | | |
| Week 0 | 19.7 ± 24.5 | 19.4 ± 21.6 | 0.952 |
| Week 12 | 20.4 ± 24.4 | 11.8 ± 18.8 | 0.054 |
| Insomnia (SL) | | | |
| Week 0 | 12.2 ± 18.9 | 14.6 ± 22.7 | 0.582 |
| Week 12 | 17.7 ± 26.4 | 7.6 ± 18.5 | 0.033 |
| Appetite loss (AP) | | | |
| Week 0 | 12.9 ± 17.8 | 18.7 ± 27.4 | 0.219 |
| Week 12 | 15.0 ± 18.1 | 13.9 ± 21.6 | 0.790 |
| Constipation (CO) | | | |
| Week 0 | 10.9 ± 20.8 | 6.9 ± 13.7 | 0.273 |
| Week 12 | 6.1 ± 13.0 | 9.0 ± 16.5 | 0.339 |
| Diarrhoea (DI) | | | |
| Week 0 | 9.5 ± 16.7 | 3.5 ± 10.3 | 0.034 |
| Week 12 | 11.6 ± 21.0 | 4.2 ± 11.1 | 0.033 |
| Financial difficulties (FI) | | | |
| Week 0 | 51.7 ± 36.0 | 56.2 ± 34.5 | 0.527 |
| Week 12 | 44.9 ± 40.0 | 40.3 ± 36.4 | 0.553 |

*P* values were determined by Student's *t* test.

**Table S14. Comparison of the incidence of the adverse events between the two groups during the placebo-controlled period.**

| Adverse events | Total, n | Placebo (n=50) | | | Thalidomide (n=49) | | | *P* |
| --- | --- | --- | --- | --- | --- | --- | --- | --- |
|  |  | Cases, n | Incidence, % | Days, median | Cases, n | Incidence, % | Days, median |  |
| [Drowsiness](http://www.youdao.com/w/drowsiness/#keyfrom=E2Ctranslation) | 33 | 12 | 24 | 4.5 | 21 | 42.9 | 3 | 0.057 |
| Dizziness | 29 | 9 | 18 | 1 | 20 | 40.8 | 2 | 0.016 |
| Fatigue | 27 | 12 | 24 | 2 | 15 | 30.6 | 3 | 0.505 |
| Pyrexia | 18 | 6 | 12 | 1 | 12 | 24.5 | 1 | 0.125 |
| Sore throat | 13 | 6 | 12 | 1.5 | 7 | 14.3 | 5 | 0.774 |
| Rash | 12 | 3 | 6 | 6 | 9 | 18.4 | 4 | 0.071 |
| Abdominal pain | 10 | 3 | 6 | 2 | 7 | 14.3 | 2 | 0.200 |
| Nausea | 10 | 2 | 4 | 2 | 8 | 16.3 | 1.5 | 0.051 |
| Anorexia | 7 | 3 | 6 | 2 | 4 | 8.2 | 6.5 | 0.715 |
| Flustered | 7 | 3 | 6 | 1 | 4 | 8.2 | 2.5 | 0.715 |
| Constipation | 6 | 1 | 2 | 78 | 5 | 10.2 | 5 | 0.112 |
| Limbs edema | 6 | 1 | 2 | 1 | 5 | 10.2 | 2 | 0.112 |
| Headache | 5 | 3 | 6 | 1 | 2 | 4.1 | 5.5 | 0.999 |
| Diarrhea | 5 | 2 | 4 | 2 | 3 | 6.1 | 1 | 0.678 |
| Limb numbness | 3 | 0 | NA | NA | 3 | 6.1 | 6 | 0.118 |
| Facial edema | 3 | 0 | NA | NA | 3 | 6.1 | 2 | 0.118 |
| Myalgia | 3 | 2 | 4 | 1.5 | 1 | 2.0 | 1 | 0.999 |
| Nosebleed | 3 | 0 | NA | NA | 3 | 6.1 | 2 | 0.118 |
| Ostealgia | 3 | 1 | 2 | 1 | 2 | 4.1 | 1 | 0.617 |
| Upper respiratory tract infection | 3 | 1 | 2 | 1 | 2 | 4.1 | 4 | 0.617 |
| Skin itch | 1 | 1 | 2 | 1 | 0 | NA | NA | 0.999 |
| Depression | 1 | 0 | NA | NA | 1 | 2.0 | 10 | 0.495 |
| Parotitis | 1 | 0 | NA | NA | 1 | 2.0 | 2 | 0.495 |
| Tinnitus | 1 | 1 | 2 | 1 | 0 | NA | NA | 0.999 |
| Xerostomia | 1 | 0 | NA | NA | 1 | 2.0 | 4 | 0.495 |

*P* values were determined by Fisher’s exact test.

**Table S15. Adverse events in patients upon thalidomide in the extension phase.**

| Adverse events | Cases, n | Incidence, % | Grade |
| --- | --- | --- | --- |
| [Drowsiness](http://www.youdao.com/w/drowsiness/#keyfrom=E2Ctranslation) | 18 | 20.0 | I, II |
| Dizziness | 16 | 17.8 | I, II |
| Fatigue | 12 | 13.3 | I, II |
| Rash | 9 | 10.0 | I, II |
| Constipation | 7 | 7.8 | I, II |
| Pyrexia | 6 | 6.7 | I, II |
| Limb numbness | 5 | 5.6 | I, II |
| Nausea | 5 | 5.6 | I, II |
| Anorexia | 4 | 4.4 | I, II |
| Cold | 4 | 4.4 | I, II |
| Limb edema | 4 | 4.4 | I |
| Abdominal pain | 3 | 3.3 | I |
| Nosebleed | 3 | 3.3 | I |
| Ostealgia | 3 | 3.3 | I |
| Sore throat | 3 | 3.3 | I |
| Cough | 2 | 2.2 | I |
| Diarrhea | 2 | 2.2 | I |
| Flustered | 2 | 2.2 | I |
| Glossoplegia | 2 | 2.2 | I, II |
| Xerostomia | 2 | 2.2 | I |
| Amenorrhea | 1 | 1.1 | I |
| Chest pain | 1 | 1.1 | I |
| Depression | 1 | 1.1 | I |
| Facial edema | 1 | 1.1 | I |
| Headache | 1 | 1.1 | I |
| Insomnia | 1 | 1.1 | II |
| Lymphadenectasis | 1 | 1.1 | I |
| Myalgia | 1 | 1.1 | I |
