# Supplementary material for: Safety and efficacy of thalidomide in patients with transfusion-dependent β-thalassemia: a randomized clinical trial
Source: Signal Transduct Target Ther. 2021 Nov 18;6:405. doi: 10.1038/s41392-021-00811-0 (PMC8602273; doi:10.1038/s41392-021-00811-0)
Supplement: Supplementary file 2 — Protocol [file 41392_2021_811_MOESM2_ESM.pdf]

A multicenter, randomized, double-blinded, placebo-controlled phase II clinical trial of thalidomide for the treatment of transfusion-dependent β-thalassemia major and intermediate

Clinical trial protocol

Final version

Version number: Version 2.0

Version date: April 23, 2018

Research team leader unit: Chinese Academy of Medical Sciences

Research person in charge: Guangbiao Zhou

Contents

Project members and division of labor........ 1

The preliminary screening flowchart........ 3

Scheme summary........ 4

1 Research background........ 13

1.1 Overview........ 13

1.2 Basis for project establishment........ 15

1.3 Selection of the combination therapy and control group........ 19

1.4 Basic principles for establishing the end point........ 20

1.5 Patient self-assessment results (CPRO)........ 20

2 Research purpose........ 22

2.1 Main purpose of the study........ 22

2.2 Secondary purpose of the study........ 22

3 Research plan and enrollment........ 23

4 Research population........ 24

4.1 Participant selection........ 24

4.2 Inclusion criteria........ 24

4.3 Exclusion criteria........ 24

5 Test process........ 26

5.1 Visit evaluation plan........ 26

5.2 Visit evaluation schedule........ 27

5.3 Informed consent/data protection agreement........ 28

5.4 Screening period........ 28

5.5 Randomization........ 30

5.6 Research treatment........ 30

5.7 Inspection and evaluation of each visit point during the study treatment period........ 36

5.8 Study completion assessment........ 39

5.9 Final analysis time........ 41

6 Efficacy and safety evaluations........ 42

6.1 Primary end-point........ 42

6.2 Change in the amount of Hb........ 42

6.3 Secondary end-points........ 42

6.4 Exploratory end-points........ 43

6.5 Evaluation of efficacy........ 43

6.6 Adverse events........ 44
7 Implementation of the blindness, blindness breaking and unblinding........ 51
7.1 Implementation of the blindness........ 51
7.2 Blindness breaking of individual cases in emergency circumstances........ 51
7.3 Regulations of unblinding........ 51
8 Data processing and statistical analysis........ 53
8.1 Data processing........ 53
8.2 Statistical analysis plan........ 54
9 Independent Assessment Committee (IAC)........ 59
10 Ethics........ 60
10.1 Ethics committee........ 60
10.2 Informed consent........ 60
10.3 Participants' information privacy........ 60
11 Clinical trial management........ 61
11.1 Scheme formulation and implementation........ 61
11.2 Experimental training........ 61
11.3 Supervision and inspection of the trial........ 61
11.4 Drug administration........ 62
11.5 Information preservation........ 62
12 Publication of the research results........ 63
13 Early termination of a single center........ 64
References........ 65
Annex 1 ECOG Performance Status........ 67
Annex 2 New York Heart Association (NYHA) Functional Classification........ 68
Annex 3 Common Terminology Criteria for Adverse Events v3.0 (CTCAE)........ 69
Annex 4 Classfication Criteria of Neurotoxicity........ 78
Annex 5 Relationship Between Adverse Events and Experimental Drugs........ 79
Annex 6 Important Information for Participants and Informed Consent Documentation........ 80
1 Research background........ 80
2 Research purpose........ 80
3 Possible benefits of participating in the study........ 81
4 Introduction of research drugs........ 82
5 Research process........ 83
5.1 Informed consent........ 83

5.2 Screening.......83

5.3 Treatment.......84

5.4 Follow-up.......84

5.5 Research period.......84

6 Things you should pay attention to in your research.......85

7 Possible risks of participating in the study.......86

7.1 Thalidomide side effects.......86

7.2 Response.......86

7.3 Those who are not suitable for participation in the study.......87

8 Related costs.......88

9 Confidentiality of research records.......88

10 Participants' rights.......88

11 Consulting contact information.......89

Annex 7 Questionnaire on Participants' Health Status.......90

Annex 8 Health Questionnaire.......95

Annex 9 World Medical Association Declaration of Helsinki.......98

A. Introduction.......98

B. Basic principles for all medical research.......99

C. Additional principles for medical research combined with medical care.......101

Annex 10 The Risk Management Plan of Thalidomide to Prevent Pregnancy.......103

1 Risk of pregnancy.......103

2 Classification of the participants.......103

3 Medication guidance for participants.......103

4 Contraceptive measures.......104

5 Pregnancy testing.......105

6 Risk management measures during the trial.......106

Annex 11 Thalidomide Medication Guidance.......108

Annex 12 Thalidomide Information Sheet.......111

Annex 13 Emergency Response Plan for Pregnancy.......114

Project members and division of labor

Applicant's name	Guangbiao Zhou	
Applicant's unit	Chinese Academy of Medical Sciences	
Applicant's contact number	15801289902	
Project name	A multicenter, randomized, double-blinded, placebo-controlled phase II clinical trial of thalidomide for the treatment of transfusion-dependent β-thalassemia major and intermediate	
Applicant's professional title	Researcher	
Applicant's degree	Medical PhD	
Key members of the team	Name	Division
Senior title	Guangbiao Zhou	Project leader of the scientific research
	Saijuan Chen	Experimental design, results interpretation and paper modification
	Jiangming Chen	Case collection and clinical observation
	Weijian Zhu	Case collection and clinical observation
	Xiaoqin Chen	Experimental design
	Ying Huang	Hb analysis and mechanism research
	Ken Huang	Clinical observation
	Jianquan Xu	Clinical observation
	Yi Huang	Clinical observation
	Wenqiang Wu	Clinical observation
	Songhua Liu	Clinical observation
	Ronglan Li	Patient follow-up and records
	Shuying Chen	Drug custody and distribution registration
	Guohui Li	Case collection and clinical observation
	Sheng He	Research on single nucleotide polymorphism

Intermediate title	Weida Wang	Experimental design
	Ning Cai	Clinical observation
	Yun Tan	Gene sequencing and mechanism research
	Liwei Qu	Fluorescence quantitative PCR
	Jinyan Li	Clinical observation
Junior title	Jie Liu	Statistical analysis
	Lan Huang	Clinical observation
	Meiling Hu	Clinical observation
	Huanju Yang	Clinical observation
	Qiyang Lu	Cell separation
	Tianying Luo	Cell separation
	Meiguang Zhou	Clinical observation
Multi-center cooperation unit and person in charge	Principal	Cooperation unit
	Guangbiao Zhou	State Key Laboratory of Molecular Oncology, Chinese Academy of Medical Sciences
	Saijuan Chen	State Key Laboratory of Medical Genomics, Ruijin Hospital affiliated with Shanghai Jiao Tong University School of Medicine
	Jiangming Chen	Department of Hematology, Wuzhou Gongren Hospital
	Xiaoqin Chen	State Key Laboratory of Oncology in South China, Sun Yat-Sen University Cancer Center
	Weijian Zhu	Department of Hematology, Zhuhai People’s Hospital
	Ken Huang	Department of Hematology, Affiliated Hospital of Youjiang Medical University for Nationalities
	Yi Huang	Department of Hematology, Guigang People’s Hospital
	Jianquan Xu	Department of Hematology, Yulin Guinan Hospital
	Wenqiang Wu	Department of Hematology, Wuzhou Red Cross
	Songhua Liu	Department of Hematology, Hospital of Traditional Chinese Medicine of Wuzhou City

The preliminary screening flowchart

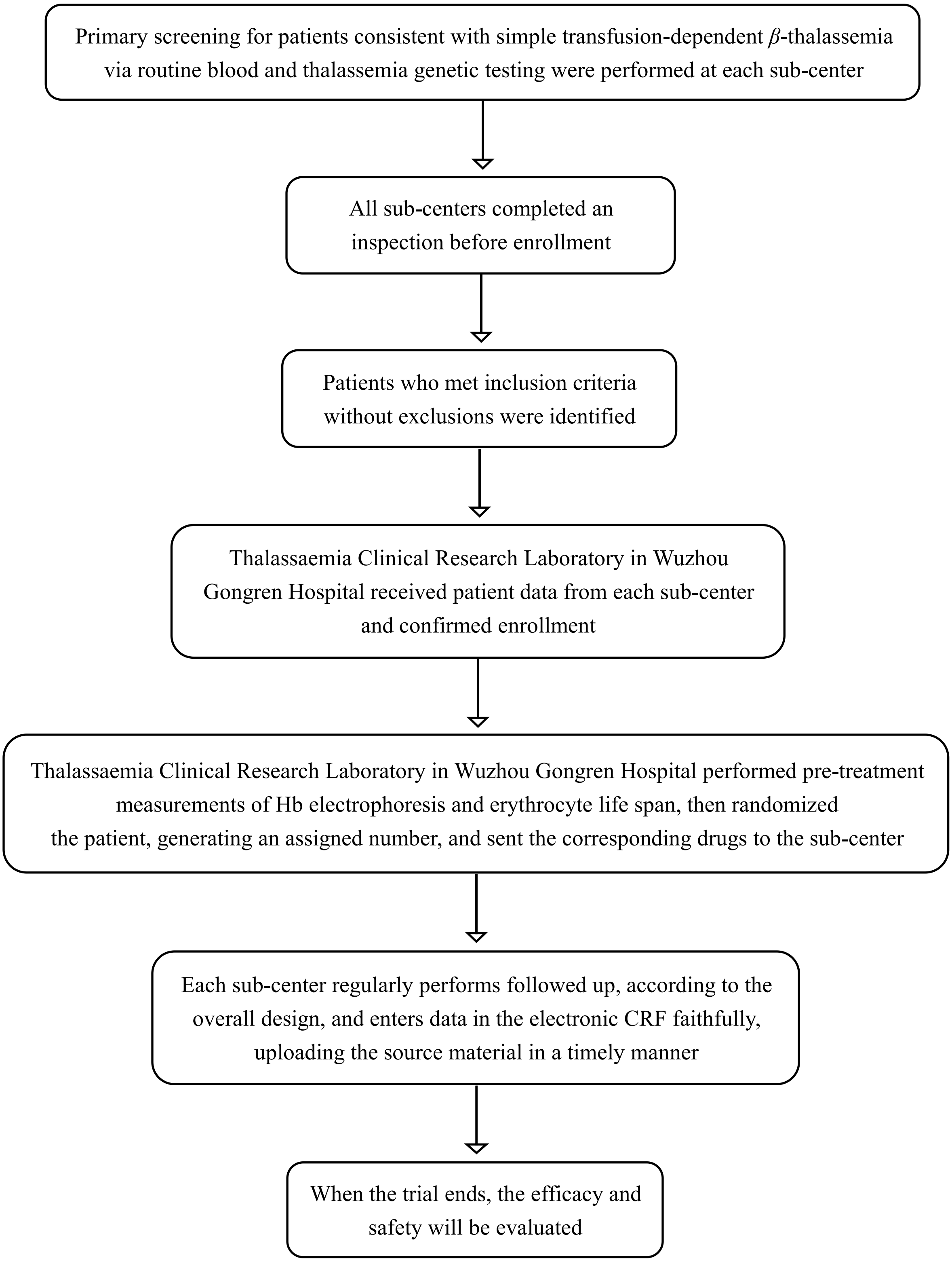

Figure 1. The preliminary screening flowchart for thalassaemia clinical research sub-centers

Scheme summary

Scheme title	A multicenter, randomized, double-blinded, placebo-controlled phase II clinical trial of thalidomide for the treatment of transfusion-dependent *β*-thalassemia major and intermediate
Institution	Wuzhou Gongren Hospital
Indications	Transfusion-dependent *β*-thalassemia major and intermediate
The study drug	Thalidomide, 25 mg/tablet
Objective	Main objective: to evaluate the efficacy of thalidomide for the treatment of transfusion-dependent *β*-thalassemia major and intermediate, with the main observational index as the 12-week change in the Hb (Hb) level.<br>Secondary objective: to evaluate the clinical safety of thalidomide for the treatment of transfusion-dependent *β*-thalassemia major and intermediate, and to observe the effect of thalidomide on blood transfusion dependence.
Assessment endpoints	Primary endpoint: the 12-week change in Hb level in patients treated with thalidomide and placebo.<br>Secondary endpoints: a reduction in the transfusion burden, and changes in the fetal Hb (Hb F) level, erythrocyte life span (ELS), and hemolytic markers, including the total bilirubin (TBIL), indirect bilirubin (IBIL), and lactate dehydrogenase (LDH) levels, and the reticulocyte and nucleated red cell counts. Additionally, a safety evaluation will be performed at the end of the follow-up period.

Overall design	Our study was defined as a multicenter, randomized, double-blinded, placebo-controlled phase II clinical trial and comprises two successive experimental stages: a placebo-controlled period and an extension phase.<br>Patients diagnosed with transfusion-dependent *β*-thalassemia major or intermediate are randomized to receive starch placebo or thalidomide treatment at a dose of 100-150 mg/day. Patients are transfused with red blood cells at 0.5 units/10 kg when their Hb level was < 70 g/L. The efficacy will be evaluated after 12 weeks of treatment. Patients with a poor or no elevation in Hb who are still transfusion-dependent will be able to enter the extension phase and receive thalidomide directly, at their request. Additionally, in patients with an obvious elevation in Hb who are free from blood transfusions for at least 6 weeks, the original treatment will be continuously administered for further evaluation.<br>When the last patient is enrolled and completes their first 12 weeks of follow-up, the placebo-controlled period of the study will end. All data generated during the placebo-controlled period are sealed; thus, an unblinding conference will be officially convened, and the efficacy evaluation will be conducted.<br>In the extension phase, all patients undergo thalidomide treatment. The patients are followed up every 14 days for the first 12 weeks, and every 3 months thereafter. The total observation time of the trial is expected to be 2 years. The Hb level and long-term adverse effects will be recorded and evaluated.
Experimental participants	Patients clinically diagnosed with transfusion-dependent *β*-thalassemia major or intermediate

Inclusion criteria	Patients must meet all the following inclusion criteria before enrollment:<br>1. Clinical and genetic diagnosis of β-thalassemia major or intermedia;<br>2. Transfusion dependence, defined as receiving at least eight transfusions or at least 100 ml/kg of their body weight in packed red cells per year; or frequent transfusion was required to maintain Hb > 70 g/L in the 2 years before enrollment;<br>3. Fourteen years of age or older, with no sex limitations;<br>4. Eastern Cooperative Oncology Group (ECOG) performance status score of 0 ~ 3;<br>5. The last transfusion was received 14 days or more before enrollment;<br>6. Signed written informed consent was given before entry into the study;<br>7. An estimated life expectancy of at least 3 months;<br>8. Four weeks after gastrointestinal bleeding cessation, the Hb level and reticulocyte count remain stable;<br>9. For patients with glucose-6-phosphate dehydrogenase deficiency, no abnormal hemolytic factors within the first 3 months of inclusion.

Exclusion criteria	Patients were not enrolled if they met any of the following exclusion criteria:<br>1. Use of thalidomide, hydroxyurea, decitabine, azacitidine, butyratesor, erythropoietin, or yisuishengxue granules (a traditional Chinese medicine formula, proved to be effective in patients with thalassemia in China) within 3 months before enrollment;<br>2. Deficiencies in vitamin B12 and folate;<br>3. Comorbid severe cardiopulmonary or cerebrovascular diseases;<br>4. Comorbid abnormal liver and kidney functions;<br>5. A medical history of intravenous or arterial thrombosis within 3 months;<br>6. Comorbidity with other causes of anemia;<br>7. Mental illness;<br>8. For female patients: pregnant or lactating, or of childbearing age and unwilling to take contraceptive measures;<br>9. Allergy to the drug ingredients;<br>10. Current participation in any other clinical trial;<br>11. Other conditions considered not suitable for participation by the researchers.

Sample size estimation	Based on our previous study and literature review, the Hb level in patients receiving placebo intervention was assumed to be 60 g/L and to not improve with intervention. In contrast, the Hb level in the thalidomide group was expected to rise to at least 72 g/L (i.e., at least 12 g/L higher than that in the control group). The standard deviation was presumed to be 20 g/L after 12 weeks of treatment.<br>Given that the ratio of patients assigned to the two groups is 1:1, the comparison in Hb levels was processed as an independent-samples *t*-test, with $P < 0.05$ considered statistically significant. Setting α to 0.05 and β to 0.2 in a two-sided test, and under the assumption that 10% of the patients would withdraw or drop out, we estimated that enrollment of approximately 100 patients was required, with 50 patients in each group.
Study period	April 2018 to April 2021
Randomization	Randomization was centrally conducted by an independent statistician using the RANDBETWEEN function of Microsoft Excel 2016, and two columns of random codes corresponding to the two groups of participants (a total of 100 cases) were generated, respectively. In accordance with the requirements of a double-blinded and single-simulated clinical trial, staff members not involved in the study were responsible for numbering the drugs. Sequentially numbered, opaque sealed envelopes (SNOSE) was used to maintain allocation concealment. Eligible patients were randomly allocated to treatment and control groups and the results of this process were preserved at Wuzhou Gongren Hospital in Guangxi Province.

Research treatment	The study comprises two stages:<br>1. The placebo-controlled period<br>Starch placebo and thalidomide are orally administered each night, after dinner, at an initial dose of 100 mg/day, which is increased to 150 mg/day at 3 days if no adverse effects are reported. Simultaneously, a traditional blood transfusion is combined as supplementary treatment, if necessary.<br>The blood transfusion standard is as follows: Hb < 70 g/L, or Hb > 70 g/L with cardiac function reaching grade III or above (see Annex 2 for the New York Heart Association (NYHA) Heart Disease Grading Standard).<br>The transfusion volume standard is as follows: patients complete a routine blood examination every 14 days and are transfused with leukocyte-poor red blood cells at 0.5 units/10 kg when their Hb level is < 70 g/L. If a patient receives a transfusion between day 78 and day 85 during the treatment period due to unforeseen circumstances, the timing of the efficacy evaluation will be delayed by 7 days from the day of transfusion for that particular case.<br>The efficacy will be evaluated after 12 weeks of treatment (i.e., at the end of placebo-controlled period). For all patients, the trial data of the first 12 weeks will be sealed. Subsequently, patients with a lack of efficacy can choose to enter the extension phase at their own request, while patients with a good, or even excellent, response will be continuously administered the original treatment for further evaluation in the extension phase.<br>2. The extension phase<br>All patients receive thalidomide treatment in this stage. The dosage, and blood transfusion and transfusion volume standards are the same as those in the placebo-controlled period. The patients will be followed up every 14 days for the first 12 weeks, and every 3 months thereafter. Simultaneously, concomitant medications, adverse effects, and patient compliance data will be faithfully recorded.
Time of unblinding	When the last participant (numbered 100) is enrolled into the study and completes 12 weeks of treatment, the placebo-controlled period of the study will come to an end. As all trial data from the first 12 weeks are sealed, an unblinding conference will be officially convened, and the efficacy evaluation will be conducted.

Analysis time	All data generated in both groups during the first phase will be compared and analyzed when the last patient completes their first 12 weeks of follow-up. The Hb level and long-term adverse effects will be evaluated when a majority of patients completes 12 months of follow-up in the second phase.
Emergency break	Partners, lead researchers, and sponsors will jointly determine whether to take an urgent break in treatment in cases of the following three emergencies:<br>1. The patient requires emergency aid and treatment;<br>2. Cessation of all experimental drugs does not solve the circumstances of the emergency;<br>3. The emergency measures taken in breaking cases are significantly different from those in non-breaking cases.<br>Partners, lead researchers, and sponsors will objectively try to avoid any exigent unblinding, unless the emergency break in treatment would affect the measures taken for rescue treatment.
Efficacy evaluation criteria	Response criteria were defined in terms of an elevation in Hb level and the need for transfusions as follows :<br>1. Excellent response: an elevation in the total Hb level ≥ 20 g/L, and freedom from blood transfusion for at least 6 weeks;<br>2. Good response: an elevation in the total Hb level between 10 g/L and 20 g/L, or an elevation in Hb level < 10 g/L with Hb > 70 g/L, and freedom from blood transfusion for at least 6 weeks;<br>3. No response: an elevation in the total Hb level < 10 g/L and continued transfusion-dependence.

Follow-up plan	Follow-up will be regularly performed, according to the overall design, and concomitant medications, adverse effects, and patient compliance will be faithfully recorded. Additionally, laboratory tests and evaluations of efficacy, safety, and mechanisms will be performed according to the following schedule:<br>Efficacy evaluation: routine blood and transfusion volume assessments will be regularly performed during the treatment period;<br>Safety evaluation: examinations will be performed once every month during the first 3 months, and once every 3 months thereafter. The evaluation items are as follows: (1) adverse events, such as thrombosis and neurotoxicity; (2) routine blood tests values; and (3) serum concentrations of ALT, AST, TBIL, DBIL, IBIL, Cr, and LDH.<br>Drug mechanism evaluation: clinical features and genetic background related to efficacy will be evaluated to determine the mechanism underlying thalidomide effects in *β*-thalassaemia.
Statistical analysis	1. Datasets are defined as follows:<br>1) The full analysis set (FAS) will comprise patients who take the drug at least once after enrollment, without missing data for the main evaluation indicator. Cases that lack baseline data for the evaluation indicator will be eliminated from the FAS;<br>2) The per protocol set (PPS) is based on the FAS, and will comprise patients who satisfy the inclusion and exclusion criteria, have valid baseline values and good compliance, and rigorously follow the clinical trial protocol (e.g., non-use of thalidomide, hydroxyurea, decitabine, azacitidine, butyratesor, erythropoietin, or yisuishengxue granules);<br>3) The safety set (SS) will comprise patients who take the drug at least once after enrollment and receive treatment for at least 1 month.<br>Both the FAS and PPS will used in the efficacy analysis; however, the FAS will be regarded as the primary index. Additionally, the SS will be used in the safety analysis.<br>2. Efficacy and safety indicators:<br>The Hb level is considered the primary indicator in this study, and the Hb values at different time points, as well as the elevation in Hb level, are the main criteria of efficacy. The secondary indicators include reductions in the

	transfusion burden, Hb F level, ELS, hemolytic markers, and adverse effects. After unblinding, differences between the two groups in the above indicators will be evaluated to determine the efficacy of thalidomide in treating transfusion-dependent β-thalassemia, and its safety will be evaluated in terms of long-term adverse events.<br>3. Statistical analysis<br>Data were analyzed according to participants' randomized treatment assignments, regardless of their subsequent medication status (intention- to-treat). The primary and secondary outcomes were compared between the experimental and control groups using the Student's t-test at a 2-sided α level of 5%, without correction for multiple comparisons. To further compare the dynamic Hb levels and transfusion volume between the two randomized arms, we constructed a linear mix-effect regression model in which the repeated measures were the dependent variables and intervention group was the independent variables. Data analyses were performed using IBM SPSS Statistics 26.0 (Chicago, IL, USA). All statistical tests were two-sided and P values less than 0.05 were considered statistically significant.
Results announcement	Research results will be disseminated to each sub-center in the form of a bulletin. Relevant papers will be published, with the byline decided according to actual contributions.

1 Research background

1.1 Overview

Thalassemia is the most common genetic disease in the world. According to the chain whose synthesis is impaired, it can be divided into α and β thalassemia; according to the severity of the disease, it can be divided into gene carrier, thalassaemia minor, thalassaemia intermedia, and thalassaemia major [1]. Generally speaking, thalassaemia minor and gene carriers with a clinically silent, mildly hypochromic and microcytic anemia that generally do not require treatment patients with thalassemia major need regular blood transfusion and iron chelation therapy for survival.

Patients with β-thalassemia intermedia (TI) have varying severity of anemia, symptoms appear in infancy, and their clinical manifestations are ranging in severity from thalassemia major to the β-thalassemia carrier state, with mild or moderately splenomegaly. There may be jaundice, varying skeletal deformities, and gonadal developmental retardation. In mild cases, the clinical symptoms are mild. Examination reveals microcytic hypochromic anemia and moderate Hb decrease; severe TI are similar to thalassaemia major with hepatosplenomegaly, and laboratory tests show changes in blood and bone marrow similar to thalassaemia major. Patients with severe TI require regular blood transfusions to maintain alive.

In addition, although some TI patients do not rely on regular blood transfusions, these patients lack effective treatment. Because of chronic severe anemia and iron overload, their complications are often much severer than those with TM. For this part of TI patients, intermittent blood transfusion and iron chelation therapy are reliable treatments [2]. However, due to the tightness of blood sources in our country, the limited amount of reimbursement for blood transfusion, the high expenses related to blood transfusion, and the high cost of iron chelation therapy, all these limit their being widely used. Therefore, it is of great clinical value to explore drug therapy to improve the prognosis of patients with transfusion dependent thalassemia (TDT).

Blood transfusion is the mainstay treatment for this disease. It is best to receive leucoreduced packed red blood cells to avoid blood transfusion reactions. Blood transfusion therapy can ameliorate the severity of anemia and reduce extramedullary hematopoiesis, and is a common treatment method for TI patients. The most difficult choice for TI patients is whether they need preventive blood transfusion, when to start blood transfusion and whether they need regular blood transfusion. Indications for blood transfusion in TI patients include: Hb < 70 g/L, or symptoms

such as fatigue, heart failure, skeletal deformities, and extramedullary hematopoiesis. Taher et al.'s indications for starting blood transfusions are Hb < 50 g/L, progressive enlargement of the spleen (increased by 3 cm or more per year), growth disorders or gonadal developmental retardation, severe bone deformation, decreased exercise tolerance, pregnancy, surgery, infection, and other serious complications [2].

Although blood transfusion therapy can alleviate the suffering of thalassaemia patients, it is not an effective way to achieve a cure. Long-term repeated blood transfusions will increase the risk of patients suffering from hepatitis B, AIDS and other transfusion-associated infections. At the same time, the bioavailability of iron decreases due to repeated bone marrow ineffective erythropoiesis, long-term chronic hemolysis increased intestinal iron absorption, and ineffective erythropoiesis in the bone marrow, etc. over time this can increased iron concentration and overload. As a result, the patient's heart, liver, spleen, kidney, and other organs are damaged, seriously affecting the patient's quality of life.

The use of iron chelators can increase the excretion of iron from urine and feces, but it cannot prevent the absorption of iron from the gastrointestinal tract. Usually the iron overload is evaluated after one year of beginning regular transfusion or 10 to 20 units of red blood cells are transfused. When iron overload occurs, iron chelation therapy should be administered to remove iron. Commonly used iron chelators include: (1) Deferoxamine, continuous subcutaneous injection once a night for 12 hours, or intravenous infusion of isotonic glucose solution for 8 to 12 hours, 5 to 7 days a week, long-term application. Deferoxamine has little side effects, occasionally allergic reactions, even long-term use can cause cataracts and long bone evelopmental retardation, excessive dosage can cause vision and hearing loss; combined application of vitamin C and iron chelators can increases iron excretion from urine; (2) Deferiprone 75~100 mg/kg/d, taken orally in 3 times; common adverse reactions include cytopenia and joint pain [3]; (3) Exjade (Deferasirox) is effective orally and has a long half-life. It only needs to be taken orally once a day, so clinical application is more convenient [4]. However, the above three iron chelators all require long-term use, are expensive, and impose a heavy financial burden on thalassaemia patients and families.

Splenectomy has a better effect on Hb H disease, but it is less effective on β-thalassaemia major and intermediate. Splenectomy can lead to weakened immune function, and should be performed after 5 to 6 years old and the indications should be strictly controlled. The red blood

cells of patients with Hb H disease are mainly destroyed in the spleen, so the anemia after splenectomy can be significantly improved; however, for patients with *β*-thalassaemia, the red blood cells are mainly destroyed in the bone marrow, so the efficacy of splenectomy is limited. Moreover, splenectomy is a traumatic operation, and there is a risk of weakened immunity and increased thrombosis after operation.

Hematopoietic stem cell transplantation is the current method to cure *β*-thalassemia major. If HLA-matched hematopoietic stem cell donors are available, hematopoietic stem cell transplantation should be the first choice for the treatment of severe *β*-thalassemia [5]. The optimal age for transplantation in patients with thalassemia major is 6 years old, Carrying out hematopoietic stem cell transplantation is too early, and organ function is underdeveloped, Too late to perform hematopoietic stem cell transplantation leads to increased rejection, iron deposition, organ failure, etc, which leads to a decrease in the success rate of transplantation Long-term survival rates that will not be obtained if the optimal transplant period is missed.

1.2 Basis for project establishment

1.2.1 Basic information

Thalidomide

Generic name of the drug: Fan Ying Ting

English name: thalidomide

Chemical name: N-(2,6-dioxo-3-piperidyl)-phthalimide

Structural formula:

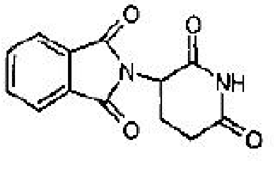

Molecular formula: $C_{13}H_{10}N_2O_4$

Molecular weight: 258.23

Dosage form: tablet

Specification: 25 mg/tablet

The clinical effect of thalidomide in the treatment of thalassemia

Thalidomide is a commonly used clinical drug, mainly used for the treatment of neoplastic

diseases and rheumatic diseases [6]. In 2008, Aguilar-Lopez et al. treated a female TI patient with thalidomide and achieved good results [7]. The patient was 21 years old and began to transfusion at 5 months of age, she was splenectmized at the age of 5 years. The patient initated 100 mg/d thalidomide in December 2001 with a Hb concentration of 46 g/L. Hb increaes to 70 g/Lwas observed after 3 months. since than, she has received thalidomide therapy uninterruptedly and never was transfused again, with Hb levels between 76 to 106 g/L. Since then, 4 cases have reported that the use of thalidomide in the treatment of NTDT has achieved good results. All reported 5 patients were effective with thalidomide, and all of them had a rapid onset of response. Most of them were response about 1 month after medication. When the drug takes effect, continue to take the drug, the increase of Hb is between 27 to 58 g/L. During the observation period of 10 months to 6 years, the efficacy of the drug can be maintained. The dose to maintain the efficacy is about 25 mg/d to 50 mg/d.

In vitro cell experiments have shown that thalidomide induces erythroid kruppel-like factor (EKLF), GATA-1, and γ-globin gene expression, producing Hb F, which is potentially mediated by the release of reactive oxygen species (ROS) [8-9]. However, as yet, there are only case reports in the clinical data regarding thalidomide for the treatment of β-thalassemia. Therefore, it is necessary to understand the value of thalidomide for the treatment of β-thalassemia in terms of its effectiveness and the appropriate population.

Based on the above-mentioned case reports, we started using thalidomide as a treatment in two cases of sibling thalassemia (with double heterozygous genes) in July 2015. Both patients showed a significant increase in Hb approximately 1 month after treatment initiation. The two patients had Hb levels of 57 g/L and 75 g/L before medication, which rose to 120 g/L and 126 g/L after medication, respectively. Up to December 2017, the two patients have been followed for 29 months and 20 months, respectively, and their Hb level has remained at approximately 110 g/L. This is consistent with the above-mentioned case reports.

Subsequently, our hospital began to systematically study the effectiveness of thalidomide for the treatment of thalassemia; this study has continued for 29 months. Among 12 patients with medium to severe thalassemia who underwent thalidomide treatment, 9 patients had a significant increase in Hb at approximately 1 month after medication initiation, rising from 51.3 ± 21.5 g/L before treatment to 103.8 ± 11.9 g/L after medication. Up to December 2017, 9 patients for whom

thalassemia was effective have been followed up for a minimum of 3 months and a maximum of 29 months; their Hb level has remained at approximately 110 g/L [10]. Therefore, based on our observations in this small sample, the effectiveness of thalidomide appears to much higher than the reported efficacy of hydroxyurea in the treatment of thalassemia.

1.2.2 Similar foreign products

Hb F inducers currently used in international research include hydroxyurea and histone deacetylase (HDAC) inhibitors, such as sodium butyrate, azacitidine, and decitabine, which are described in detail below.

Hydroxyurea

It is generally believed that hydroxyurea treats *β*-thalassemia by increasing *γ*-chain synthesis. In patients with severe β-thalassemia, hydroxyurea has an effective rate ranging 32% to 81.8%, manifested by a reduction in blood transfusions of more than 40%. Furthermore, a small number of patients (11% to 18.2%) can become transfusion-free (i.e., the Hb level is maintained at > 70 g/L without blood transfusions) [11]. Additionally, hydroxyurea is effective in 70% of patients with *β*-thalassemia intermediate (i.e., the Hb level is increased by > 10 g/L) [12]. However, the long-term use of hydroxyurea shows a downward trend [13].

Azacitidine and decitabine

Azacitidine is considered to have high potential in increasing the production of Hb F-inducing drugs. Azacytidine and decitabine act as histone deacetylases and DNA methyltransferase (DNMT) inhibitors, increasing Hb F, and can regulate gene expression levels through demethylation. After a normal birth, the promoter region of the *γ*-globin gene is gradually methylated, causing the *γ*-globin gene to be silenced, while demethylation can reopen the *γ*-globulin gene. Thus, azacytidine and decitabine can achieve the goals of increasing the Hb F level and reducing anemia. Studies have confirmed that azacitidine and decitabine alone can achieve a certain effect on *β*-thalassaemia intermediate. For example, in the study Olivieri et al. regular decitabine treatment was administered to patients with *β*-thalassemia intermediate (0.2 mg/kg subcutaneous injection, twice a week for 12 weeks), and their mean Hb level increased from 78.8 ± 8.8 g/L before treatment to 90.4 ± 7.7 g/L after treatment ($P = 0.004$) [14].

Decitabine is currently the strongest known DNMT inhibitor, and can activate gene transcription by blocking DNA methylation. Decitabine alone is used to treat *β*-thalassemia

intermediate, and some patients have more severe platelet rise, even affects the following treatment [14]. The main problems with decitabine are as follows: (1) it is a chemotherapy drug with potential carcinogenic effects; and (2) it is expensive and difficult for patients to take for a long term.

Butyric acid derivatives

Butyrate and its derivatives can selectively stimulate the γ-globin gene in the embryo or fetus, directly activating the expression of the γ-globin gene, and thereby synthesizing large numbers of γ- and α-chains and Hb F, and compensating for the deficiency in β-globin peptide chains and the resulting series of damage to red blood cells.

Sodium butyrate and arginine butyrate are considered as low-risk and effective in the treatment of sickle cell anemia and thalassemia. Studies have shown that butyric acid can increase β-globin gene expression. In addition, butyric acid can increase the histone acetylation of the β-globin gene; however, gene expression was not increased after this phenomenon. The clinical application of butyric acid is cumbersome, and its efficacy is controversial; thus, treatment with butyric acid is basically considered as not feasible.

1.2.3 The mechanism of thalidomide in the treatment of thalassemia

It is well established that the efficient induction of Hb F is an effective method for the treatment of β-thalassemia. Hb F inducers can induce γ-globin gene expression to produce high levels of Hb F expression via various epigenetic and molecular mechanisms. Currently little research has been performed to evaluate whether thalidomide can effectively induce an increase in Hb F, and the mechanism remains unknown. Furthermore, the direct target of thalidomide in the treatment of thalassemia remains unclear. However, some evidence indicates that the mechanism of action might be as follows.

Thalidomide can effectively enhance the expression of GATA-1 and EKLF in hematopoietic stem cells (HSCs) and erythroid progenitor cells derived from β-globin gene mutations, and induce γ-globin gene expression of Hb F [8].

This has been demonstrated in a number of studies; these studies suggest that the effect of thalidomide is related to the synthesis of inflammatory cytokines, such as NF-κB-induced tumor necrosis factor (TNF-α), vascular endothelial growth factor (VEGF), and prostaglandin E2 (PGE2), as well as an increased released of reactive oxygen species (ROS). The effect of thalidomide is

probably due to the ability of ROS to activate p38 mitogen-activated protein kinase (MAPK), which results in an increased level of Hb F [15].

Another study reported that thalidomide can increase the expression of GATA-1 transcription factor via the induction of signal transducer and activator of transcription 5 (STAT5) and the inhibition of external pathways that promote apoptosis in erythropoietic cells. Additionally, GATA-1 can effectively induce γ-globin gene expression of Hb F [16].

Furthermore, thalidomide induces the expression of γ-globin mRNA in a dose-dependent manner, with no effect on β-globin expression. Western blot analysis has demonstrated that thalidomide can activate the p38 MAPK signaling pathway in a time- and dose-dependent manner, increasing γ-globin gene expression by improving histone H4 acetylation [9].

1.2.4 Thalidomide as an innovative treatment of thalassemia

The current preliminary data suggests that thalidomide is more efficient, economical, and practical than are other Hb F inducers. In addition, it has fewer adverse reactions, without secondary pulmonary interstitial lesions and potential carcinogenic effects.

1.3 Selection of the combination therapy and control group

1.3.1 Existing treatment options for thalassemia

As commonly known, thalassemia is an inherited genetic defect, with no effective medical treatment as yet. Although blood transfusions and iron chelation therapy can maintain the patient's life, there is, unfortunately, no way to prevent the disease from gradually worsening. Allogeneic hematopoietic stem cell transplantation is the only way to achieve a cure. However, due to factors such as high economic costs and limited sources of matched donors, it is difficult to carry out universally.

1.3.2 Basis for choosing a blood transfusion scheme as a control

There is currently no effective medical treatment for thalassemia. Allogeneic hematopoietic stem cell transplantation is the only way to achieve a cure. However, as mentioned above, due to factors such as high economic costs, limited donor matching sources, and high treatment-related mortality, it is difficult to carry out universally. Blood transfusion therapy is the main means to prolong the life of patients, and it is also the basis of the current treatment of thalassemia. Thus, the choice of combining basic transfusion therapy with thalidomide or placebo not only protects the rights and safety of patients, but also allows the determination of the efficacy of thalidomide.

1.4 Basic principles for establishing the end point

The main efficacy endpoint of this study: changes in Hb levels at the end of follow-up.

The traditional treatment of thalassemia relies exclusively on blood transfusions to maintain the patient's Hb level at a target level. After the cessation of blood transfusions for a period of time, the patient's Hb level will gradually decline. Early data supporting the present trial showed that the Hb level in those who responded to the drug will gradually increase after 1 month of treatment, and reach a steady-state Hb level after 3 months of treatment. The blood transfusion standard in the present study was set to Hb < 70 g/L, and routine blood assessments are reviewed every 14 days. When the blood transfusion standard is reached, leucoreduced-packed red blood cells are transfused according to the standard of 0.5 units/10 kg of body weight. This study was designed to fully, and in a balanced manner, reflect the basic level of Hb in the two groups of patients with thalassemia, and at the same time allow the determination of the therapeutic effect by analyzing the difference between these two groups in terms of the amount of Hb after treatment.

At the end of follow-up, the blood transfusion volume, ELS, hemolysis response indicators (total bilirubin, indirect bilirubin, LDH, reticulocyte count, and peripheral nucleated red blood cell count), and change in Hb F level will be compared between groups, and a safety evaluation will be conducted.

Thalassemia is a hemolytic anemia. Traditional treatment can only rely on blood transfusion to maintain the patient's Hb at a target level. The comparison of blood transfusion volume and red blood cell life measurement can reflect the difference in efficacy between the two groups. Changes in hemolysis indicators indirectly reflect the efficacy. Thalidomide is a Hb F inducer, and the change of Hb F level before and after treatment can be used as an auxiliary judgment of efficacy. Secondary endpoints also include safety assessments, including the incidence of adverse events, the incidence of abnormal values in clinical laboratory tests, and other safety data (such as electrocardiograms, vital signs, special examinations, etc.). Safety The data from the beginning of the study treatment to 28 days after the end of the treatment are analyzed. However, if the participant terminates the study treatment due to an adverse event (Adverse Event AE), the data should be analyzed until the AE disappears or improves.

1.5 Patient self-assessment results (CPRO)

Patient Reported Outcomes (CPROs) are report directly from the patient, and pertain to the

patient's own health status and treatment outcomes, as well as the patient's own assessment of the clinical outcome of the disease or health condition, without the influence of a doctor or others. The clinical course of thalassemia may include anemia, hepatosplenomegaly, cardiac insufficiency, biliary calculi, high iron load, and immunodeficiency. Patients may experience disease-related symptoms (such as pain, fatigue, and recurrent infections) and treatment-related side effects that may affect their Health Related Quality of Life (HRQoL), including their physical, social, psychological, and functional health. This trial uses the TranQol-Adult_AU1.0 as a measure of the participant's self-assessment outcomes in terms of the HRQoL and disease- and treatment-related symptoms. The TranQol-Adult_AU1.0 measures five main functional domains (physical, emotional, family, study, and work). Additionally, the Core questionnaire from the Life Quality of European Organization for Research and Treatment of Cancer (EORTC) is completed at the same time as the TranQol-Adult_AU1.0. Relationships between clinical significance and efficacy and responses on these two questionnaires of the quality of life in Chinese patients with blood transfusion-dependent thalassaemia will be evaluated. In addition, participants will complete the Eortcqlq-c30, which measures five main functions (physical, occupational, emotional, cognitive, and social), and comprises three symptom scales (fatigue, nausea/vomiting, and pain), six items (dyspnea, sleep disturbances, decreased appetite, constipation, diarrhea and economic effects) and general health and quality of life scales.

2 Research purpose

2.1 Main purpose of the study

To evaluate the efficacy of thalidomide in the treatment of transfusion-dependent β-thalassemia major and intermediate, and the main observational index is the change of Hb level in 12 weeks.

2.2 Secondary purpose of the study

To evaluate the clinical safety of thalidomide in the treatment of transfusion-dependent β-thalassemia major or intermediate and observe the effect of thalidomide on blood transfusion dependence.

3 Research plan and enrollment

The research plan was launched in April 2018, and the research period is about 36 months. 100 patients are planned to be enrolled.

4 Research population

4.1 Participant selection

Before randomization, participants must meet all inclusion criteria and do not meet any of the exclusion criteria. Before randomization, the results must be reviewed by the investigator.

4.2 Inclusion criteria

Patients must meet all the following inclusion criteria before enrollment:

Patients must meet all the following inclusion criteria before enrollment:

1. Clinical and genetic diagnosis of β-thalassemia major or intermedia;

2. Transfusion dependence, defined as receiving at least eight transfusions or at least 100 ml/kg of their body weight in packed red cells per year; or frequent transfusion was required to maintain Hb > 70 g/L in the 2 years before enrollment;

3. Fourteen years of age or older, with no sex limitations;

4. Eastern Cooperative Oncology Group (ECOG) performance status score of 0 ~ 3;

5. The last transfusion was received 14 days or more before enrollment;

6. Signed written informed consent was given before entry into the study;

7. An estimated life expectancy of at least 3 months;

8. Four weeks after gastrointestinal bleeding cessation, the Hb level and reticulocyte count remain stable;

9. For patients with glucose-6-phosphate dehydrogenase deficiency, no abnormal hemolytic factors within the first 3 months of inclusion.

4.3 Exclusion criteria

Patients were not enrolled if they met any of the following exclusion criteria:

1. Use of thalidomide, hydroxyurea, decitabine, azacitidine, butyratesor, erythropoietin, or yisuishengxue granules (a traditional Chinese medicine formula, proved to be effective in patients with thalassemia in China) within 3 months before enrollment;

2. Deficiencies in vitamin B12 and folate;

3. Comorbid severe cardiopulmonary or cerebrovascular diseases;

4. Comorbid abnormal liver and kidney functions;

5. A medical history of intravenous or arterial thrombosis within 3 months;

6. Comorbidity with other causes of anemia;

7. Mental illness;

8. For female patients: pregnant or lactating, or of childbearing age and unwilling to take contraceptive measures;

9. Allergy to the drug ingredients;

10. Current participation in any other clinical trial;

11. Other conditions considered not suitable for participation by the researchers.

5 Test process

5.1 Visit evaluation plan

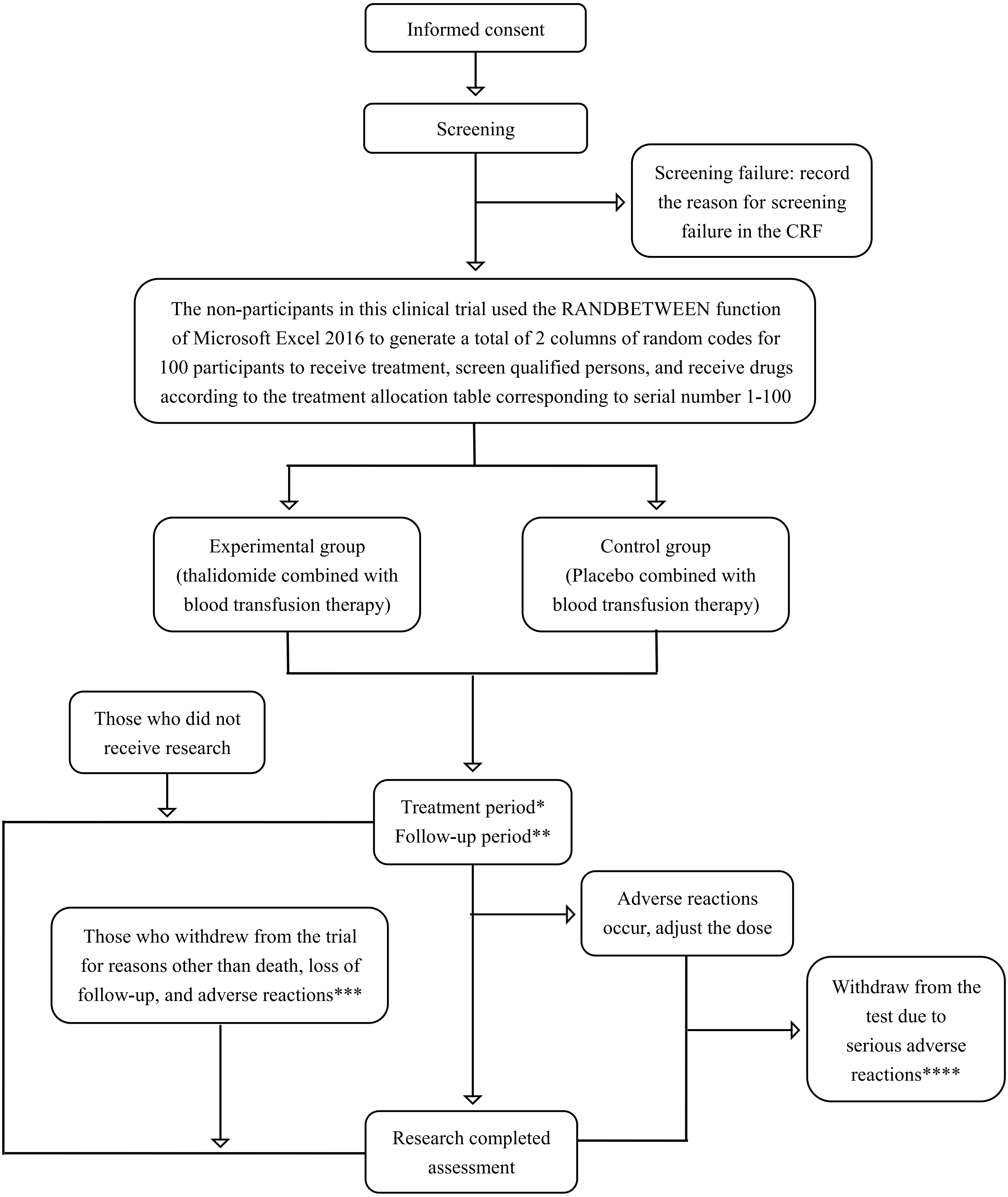

5.2 Visit evaluation schedule

**Visit**	**1**	**2**	**3**	**4**	**5**	**6**	**7**	**8**	**9**	**10**
Time compared to baseline examination	Screening	Week 2	Week 4	Week 6	Week 8	Week 10	Week 12	Month 6	Month 9	Month 12
Medication period (days)	-7~0	15	29	43	57	71	85	180	270	360
Informed consent	X									
Medical history	X									
Vital signs	X	X	X	X	X	X	X	X	X	X
Medical examination	X	X	X	X	X	X	X	X	X	X
Blood transfusion history	X	X	X	X	X	X	X	X	X	X
Routine blood	X	X	X	X	X	X	X	X	X	X
Urine routine	X									X
Stool occult blood test	X		X		X		X	X	X	X
Hb analysis	X						X			X
Thalassaemia genetic testing	X									
G-6PD detection	X									
T lymphocyte detection (CD3/CD4/CD8)	X						X			X
Liver and kidney function, myocardial enzymes	X		X		X		X	X	X	X
Folic acid, VitB12, Serum ferritin	X									
Coomb test	X									
Sugar soluble, hot soluble, acid soluble test	X									
EPO test	X									
Red blood cell life measurement	X						X			X
Blood concentration	X		X		X		X	X	X	X
Electrocardiogram	X						X			X
Echocardiography	X						X			X
Upper abdominal magnetic resonance	X						X			X
HCG	X						X			X
Record concomitant medication		X	X	X	X	X	X	X	X	X
Compliance assessment		X	X	X	X	X	X	X	X	X
Record adverse events		X	X	X	X	X	X	X	X	X

5.3 Informed consent/data protection agreement

The investigator is responsible for explaining the purpose, method, benefits and potential risks of this clinical trial to each participant, and obtaining the informed consent signed by the participants of the clinical trial. Prior to any procedures related to clinical trials, informed consent must be obtained. For those who cannot sign the informed consent for any reason, their parents, legal guardians or protectors must sign the informed consent. By signing the informed consent form, the participant/patient must also agree to allow the clinical research auditor/health survey organization to verify the original data obtained from the clinical research in order to determine the reliability of the clinical research data results.

The informed consent signed and signed by the participants of the clinical trial must be properly preserved by the investigator, and the proof of informed consent must also be attached to the case report form and the treatment record form of the clinical trial participant.

5.4 Screening period

5.4.1 Screening period inspection and evaluation

The following checks or evaluations should be completed during screening:

1. Inquiry of medical history: including medical history, previous genetic diagnosis, pre-screening treatment and other comorbidities, medication history, fever and blood transfusion history in recent 3 months;

2. The accurate number and volume of blood transfusions (RBC units) in the last 12 months;

3. Physical examination: including height, weight, blood pressure, heart rate examination;

4. Routine blood tests: including blood white blood cell count, Hb, red blood cell count, platelet count, reticulocyte count, nucleated red blood cell count;

5. Hb analysis: including Hb A, Hb A2, Hb F;

6. Thalassaemia genetic testing;

7. G-6PD detection;

8. T lymphocyte detection (CD3/CD4/CD8);

9. Blood biochemical tests: ALT, AST, TBIL, DBIL, IBIL, BUN, Cr, LDH;

10. Anti-human globulin test, triple dissolution test (sugar dissolution, hot dissolution, acid dissolution);

11. Three items of anemia (folic acid, VitB12, serum ferritin), EPO determination;

12. Red blood cell life measurement;

13. Routine urine tests: white blood cells, urine protein, red blood cells;

14. Stool occult blood test;

15. Standard 12-lead ECG;

16. Heart color ultrasound, upper abdominal magnetic resonance;

17. Pregnancy test: suitable for women of childbearing age.

According to the enrollment/exclusion criteria, qualified participants were enrolled.

5.4.2 Information to be collected when screening fails

If the participant fails to randomize within 21 days for any reason after signing the informed consent form, the participant is a screening failure. The screening inspection and evaluation results and the reason for the screening failure must be recorded in the CRF.

The reason for the failed screening may be one of the following:

1. Unacceptable medical history/ simultaneously diagnosed disease;
2. Unacceptable inspection results;
3. Failure to meet diagnostic/severity criteria;
4. Use of banned drugs / treatments;
5. Participant withdrew from the trial;
6. Others.

5.5 Randomization

Randomization was centrally conducted using the RANDBETWEEN function of Microsoft Excel 2016, and two columns of random codes corresponding to the two groups of participants (a total of 100 cases) were generated, respectively. In accordance with the requirements of a double-blinded and single-simulated clinical trial, staff members not involved in the study were responsible for numbering the drugs. Sequentially numbered, opaque sealed envelopes (SNOSE) was used to maintain allocation concealment. Eligible patients were randomly allocated to treatment and control groups and the results of this process were preserved at Wuzhou Gongren Hospital in Guangxi Province.

5.6 Research treatment

5.6.1 Research drugs, research treatments and experimental drugs

Thalidomide (Fan Ying Ting)/placebo is the study drug for this trial, which is a tablet, 25 mg/tablet. It's purchased from Jiangsu Changzhou Pharmaceutical Factory and preserved at room temperature.

5.6.2 Treatment programs

The study comprises two stages:

1. The placebo-controlled period

Starch placebo and thalidomide are orally administered each night, after dinner, at an initial dose of 100 mg/day, which is increased to 150 mg/day at 3 days if no adverse effects are reported. Simultaneously, a traditional blood transfusion is combined as supplementary treatment, if necessary.

The blood transfusion standard is as follows: Hb < 70 g/L, or Hb > 70 g/L with cardiac function reaching grade III or above (see Annex 2 for the New York Heart Association (NYHA) Heart Disease Grading Standard).

The transfusion volume standard is as follows: patients complete a routine blood examination every 14 days and are transfused with leukocyte-poor red blood cells at 0.5 units/10 kg when their Hb level is < 70 g/L. If a patient receives a transfusion between day 78 and day 85 during the treatment period due to unforeseen circumstances, the timing of the efficacy evaluation will be delayed by 7 days from the day of transfusion for that particular case.

The efficacy will be evaluated after 12 weeks of treatment (i.e., at the end of

placebo-controlled period). For all patients, the trial data of the first 12 weeks will be sealed. Subsequently, patients with a lack of efficacy can choose to enter the extension phase at their own request, while patients with a good, or even excellent, response will be continuously administered the original treatment for further evaluation in the extension phase.

2. The extension phase

All patients receive thalidomide treatment in this stage. The dosage, and blood transfusion and transfusion volume standards are the same as those in the placebo-controlled period. The patients will be followed up every 14 days for the first 12 weeks, and every 3 months thereafter. Simultaneously, concomitant medications, adverse effects, and patient compliance data will be faithfully recorded.

5.6.3 Window period for research treatment and evaluation

The window period from signing the informed consent form to starting the study treatment is 21 days.

The window period from randomization to start of study treatment is 3 days, but if the medical condition cannot be received within the 3 day window period, it can be extended to 2 weeks. If the study treatment is not received within 2 weeks, If the study treatment is not received within 2 weeks, it cannot be conducted even if the body recovers.

The window of study treatment delay caused by AE is 14 days, and more than 14 days will be treated as a withdrawal group. However, if the participant obviously benefits from the study treatment and the reason for the delayed medication disappears, the study can be restarted with the consent of the investigator and the sponsor. This selection should be made very cautious to determine the safety of the participant and to evaluate whether the benefit outweighs the risks to the participant.

5.6.4 No study treatment after randomization

For patients who did not receive study treatment within 2 weeks of randomization, a “study completion assessment” was performed to record the reasons for not receiving study treatment. Patients may not receive study treatment after randomization due to one of the following reasons:

1. Death;

2. Security/effectiveness concerns;

3. Hope to participate in other clinical trials;

4. The researchers think it is inappropriate to participate;

5. Other reasons.

5.6.5 Research treatment records

The use of study drugs should be recorded in detail in the participant's medical record and recorded in the CRF. Try to ensure that the participants come to the hospital strictly for the visit time specified in the plan.

Thalidomide is an oral drug, and participants must fill in the medication log carefully every day. At each visit, according to the medication log card, count pills and the information provided by the participant or caregiver to record the medication.

Participants should be instructed to return the remaining medication and packaging regardless of whether all medications have been taken, and at no time to transfer pills from one vial to another. This information should be recorded in the original record at each visit.

5.6.6 Duration of treatment

All data generated in both groups during the first phase will be compared and analyzed when the last patient completes their first 12 weeks of follow-up. The Hb level and long-term adverse effects will be evaluated when a majority of patients completes 12 months of follow-up in the second phase.

5.6.7 Criteria for early termination or withdrawal of participants:

Any of the following occurs in any patient during the trial period, the trial shall be terminated:

1. When a severe toxic reaction occurs, and the researchers determine it is related to the drug being studied, stop the drugs in patients with symptomatic treatment, reduce to a class II hematology toxicity or under class I non-hematologic toxicity, patient can be cured with a reduced test doses and the doses can be reduced at most for the second time, if there is still a serious toxicity reaction ($\geq$ 3 degrees non-blood toxicity (except for hair loss) or 4 degrees blood toxicity), then terminate the test;

2. Fecal occult blood test should be monitored during the follow-up. If positive, gastrointestinal bleeding should be excluded. If gastrointestinal bleeding is confirmed, then terminate the test;

3. Patients with G-PD-induced hemolytic anemia occurs between the 1 and 85 days after

treatment begins;

4. Uncontrollable concomitant diseases;

5. The patient requests withdrawal;

6. Poor compliance;

7. The researchers concluded that the treatment should end.

Participants have the right to withdraw from the study at any time for any reason, whether during the supportive trial or formal trial phase of the study. The investigator should contact the patients by telephone or follow-up, or through his or her relatives, to get as complete a picture as possible of the reasons for withdrawal

The investigator also has the right to determine the withdrawal of the participants from the study in the event of relapse of original disease. The study cannot be continued because of a serious adverse event, violation of treatment protocols, poor compliance, taking other drugs that affect the evaluation of efficacy during the trial, management or another reasons. Unnecessary withdrawal should be avoided because too many withdrawals will make study results unreliable. If a patient withdraws from the study due to adverse events or abnormal laboratory test results, this case should be recorded in the case report form. Patients who withdraw early cannot be replaced by other participants.

5.6.8 Research the information, packaging, and labeling of therapeutic drugs.

Both thalidomide and placebo are tablets, packaged in white plastic bottles, 20 tablets/bottle, 25 mg/tablet. This product should be placed at room temperature and kept in a dry place away from light. The validity period is 3 years.

Thalidomide/placebo will be packaged in a blinded manner. One bottle is a minimum package, 20 tablets/bottle, and the box label must be marked as a special drug for clinical research, indicating the trial protocol number, drug name, packaging, traits, usage and dosage, drug number, participant’s number, initials, medication Treatment course, production batch number, expiration date, storage conditions and company name. The bottle label should be marked as a special drug for clinical research, indicating the trial protocol number, drug name, specifications, production batch number, expiration date, storage conditions, drug number, participant’s number, initials, medication course and company name.

5.6.9 Guiding principles for medication

1. Guiding principles for research medication

Start taking 100 mg/d and take it orally after dinner every day. Observe whether there are adverse reactions such as rash and limb numbness. If it is tolerable, increase to 150 mg/d after 3 days and continue taking. If the participant has drug-related adverse reactions, adjust the medication according to the principle of dose adjustment of 5.4.10.

2. Combined medication

Throughout the study, the participants were not allowed to use other stimulating erythroid hematopoietic drugs except for the test drugs, including erythropoietin (EPO), hydroxyurea, Yisuishengxue granules, decitabine, azacitidine, Butyrate drugs, various Chinese herbal medicines, etc., but iron chelators can be used.

Researchers can take appropriate supportive treatment after evaluating the relationship between adverse events and medication. Record the start and duration of supportive treatment in the CRF. These treatments include antiemetics, antidiarrheal, antipyretic, antiallergic, diabetes treatment, antihypertensive medications, analgesics, antibiotics, and other blood products.

Non hematologic toxicity can be treated with corresponding drugs at grade II, and recorded in the combination of drugs.

Basic diseases (such as hypertension, diabetes, etc.) should be given maintenance treatment.

All concomitant medications received by the patient and the reasons for their medication should be fully recorded in the CRF.

5.6.10 Dose adjustment

1. The main adverse reaction of thalidomide is neurotoxicity:

1) When grade 2 neurotoxicity occurs, the dose is reduced from 150 mg/d to 50 mg/d;

2) When grade 3 nervous system toxicity occurs, the drug is temporarily stopped, and the drug is started at 50 mg/d after the symptoms disappear.

3) The treatment was discontinued when the neurological toxicity was grade 4 and the trial was withdrawn.

A maximum of two dose reductions are allowed, ie the lowest dose is 50 mg once daily.

2. For non-neurological toxicity, the treatment is as follows:

When the patient has ≥ 3 grade of non-hematological toxicity (except hair loss), stop the drug

first and treat it symptomatically. If the toxic reaction cannot be recovered to ≤ 1 degree on 14 days, the study will be withdrawn; if the toxic reaction can be recovered to ≤ 1 degree on 14 days, continue Treatment, but the dose per dose is reduced to 1/2.

If the patient reappears ≥ 3 degrees of non-hematological toxicity (except hair loss) during treatment after dose reduction, the drug will be stopped first and symptomatic treatment will be taken. If the toxicity cannot be recovered to ≤ 1 degrees within 14 days, the study will be withdrawn;

If the toxic reaction can be restored to ≤ 1 grade in 14 days, treatment can be continued, but the dose is reduced to 1/4.

3. Dose escalation

The investigator believes that other non-hematological toxicity ≥ 3 grades that may be related to thalidomide (or placebo) should be discontinued when thalidomide (or placebo) is restored to ≤ 1 grade toxicity or baseline level At the time, after reducing the dose according to the plan, closely monitor and gradually resume medication. Specific method: add 1 tablet (25 mg) every 5 days to the above dose adjustment target level.

5.6.11 Concomitant treatment/co-administration

Refers to all drugs (not including experimental drugs) and therapies used randomly until the end of treatment. The drugs include not only the drugs prescribed by the doctor, but also all non-prescription drugs, vitamins, herbs, etc.

1. Recommended concomitant treatment

Venous thrombosis is a common side effect of thalidomide, so it is strongly recommended that participants with potential risk of thrombosis receive prophylactic treatment during the trial, and oral low-dose aspirin is the first choice, if aspirin contraindications (G-6PD deficiency, etc.) exist, such participants should receive full-dose warfarin or other antithrombotic prophylactic drugs (according to the investigator's choice), or low molecular weight heparin. The use of anticoagulants for the prevention of VTE (venous thromboembolic events) will be determined by the investigator and will be selected based on the individual's thrombosis risk, bleeding risk, and compliance with antithrombotic drug treatment to obtain the best individual efficacy.

2. Possible concomitant treatment

1) Researchers can take corresponding supportive treatment after evaluating the relationship

between adverse events and medication. Record the start and duration of supportive treatment in the CRF. These treatments include antiemetics, antidiarrheal, antipyretic, antiallergic, diabetes treatment, antihypertensive medications, analgesics, antibiotics, and other blood products;

2) Non hematologic toxicity can be treated with corresponding drugs at grade II, and recorded in the combination of drugs;

3) Basic diseases (such as hypertension, diabetes) should be given maintenance treatment;

4) All concomitant medications received by the patient and the reasons for their medication should be fully recorded in the CRF.

3. Disabled concomitant treatment

1) Throughout the study, the participants were not allowed to use other stimulating erythroid hematopoietic drugs except for the test drugs, including erythropoietin (EPO), hydroxyurea, Yisuishengxue granules, decitabine, azacitidine, Butyrate drugs, various Chinese herbal medicines, etc., but iron chelators can be used;

2) Erythropoietin (EPO) treatment is not allowed.

5.7 Inspection and evaluation of each visit point during the study treatment period

The participants were examined and evaluated according to the visit plan (see the visit assessment plan table). During the trial period, the investigator department can add other necessary inspection items or inspection times according to clinical needs, and the results are recorded in the “unplanned visit” of the CRF.

The visit time of each course of treatment, the day when thalidomide/placebo was taken as the first day, the previous day was -1d, and so on.

The evaluation of adverse events should be from the beginning of the study treatment to 28 days after the end of the study treatment. If the participant terminates the study treatment due to the adverse event, it should be evaluated until the adverse event disappears or improves.

5.7.1 Follow-up during the screening period (-1d, window period ± 7d)

1. Vital signs, including body temperature, heart rate, breathing, blood pressure;

2. Physical examination;

3. ECOG performance status;

4. Record blood transfusion history;

5. Routine blood, including: white blood cell count, absolute neutrophil count, absolute

lymphocyte count, absolute monocyte count, absolute eosinophil count, absolute basophil count, red blood cell count, Hb, platelet count;

6. Urine routine, including urine protein, urine glucose, urine occult blood, and urine leukocytes;

7. Stool occult blood test;

8. Hb electrophoresis analysis;

9. G-6PD detection;

10. T lymphocyte detection (CD3/CD4/CD8);

11. Blood biochemistry, before the day of administration. Including AST/ALT, total bilirubin, direct bilirubin, indirect bilirubin, total protein, albumin, globulin, lactate dehydrogenase, uric acid and urea nitrogen, creatinine;

12. Electrocardiogram, monitoring the following indicators: heart rate, RR interval, QRS interval, QT/QTc;

13. Myocardial enzymes;

14. Anti-human globulin test and triple dissolution test;

15. Three items of anemia, EPO determination;

16. Red blood cell life measurement;

17. Cardiac ultrasound and magnetic resonance of the upper abdomen;

18. Pregnancy tests, only in fertile female participants, need not be repeated if results are available 1-3 days prior to screening;

19. Symptoms and signs that existed within one week before treatment, collect the symptoms and signs that existed within one week before treatment within 3 days before starting treatment, such as: fatigue, nausea, vomiting (times/day), abdominal pain, diarrhea (times/day), Constipation, fever (˚C), rash, palpitations, chest tightness, drowsiness, dizziness, vision loss, edema (parts), numbness of the fingertips, etc;

20. Combined medication and therapy (refers to medication and therapy other than research treatment).

5.7.2 The time settings of follow-up during the trial (window period ± 7 days)

Follow-up every 14 days for the first 3 months after medication to detect routine blood Hb levels. Follow-up to carry out the following inspections and evaluations, researchers can add other

necessary inspection items and increase the number of inspections according to clinical needs. The results are recorded as “Unplanned Visits” in CRF. And the follow-up of day 29, 57, 85, 180, 270 is as follows:

1. Vital signs, including body temperature, heart rate, breathing, blood pressure;

2. Physical examination;

3. Blood transfusion unit during treatment;

4. Routine blood, including: white blood cell count, red blood cell count, nucleated red blood cell count, reticulocyte count, Hb, platelet count;

5. Stool occult blood test;

6. Increased Hb electrophoresis and erythrocyte longevity measurement items at the 12th week of treatment;

7. Blood biochemistry, including AST, ALT, total bilirubin, direct bilirubin, indirect bilirubin, total protein, albumin, globulin, lactate dehydrogenase, uric acid and urea nitrogen, creatinine;

8. Myocardial enzymes;

9. Record of adverse events that occurred during the course of treatment;

10. Combined medications and therapies (refers to medications and therapies not researched).

5.7.3 The end of the treatment (360 days of treatment, window period ± 7 days)

All participants receiving study treatment (except death, loss of follow-up, and participants withdrawing from the trial) were given a “end of treatment” visit (window period ± 7 days) after the end of treatment. Inspection and evaluation:

1. Vital signs, including body temperature, heart rate, breathing, blood pressure;

2. Physical examination;

3. ECOG performance status;

4. Pregnancy tests are limited to female participants of fertility;

5. Record the unit and frequency of blood transfusion during the course of treatment;

6. Routine blood, including: white blood cell count, red blood cell count, nucleated red blood cell count, reticulocyte count, Hb, platelet count;

7. Red blood cell life measurement;

8. Urine routine, including urine protein, urine glucose, urine occult blood, and urine leukocytes;

9. Stool occult blood test;

10. Hb electrophoresis;

11. T lymphocyte detection (CD3/CD4/CD8);

12. Blood biochemistry, including AST, ALT, total bilirubin, direct bilirubin, indirect bilirubin, total protein, albumin, globulin, lactate dehydrogenase, blood glucose, uric acid and urea nitrogen, creatinine;

13. Electrocardiogram, testing the following indicators: heart rate, PR interval, QRS interval, QT, QTc;

14. Myocardial enzymes;

15. Cardiac ultrasound and magnetic resonance of the upper abdomen;

16. Evaluation of adverse events;

17. Combine medication and therapy;

18. Compliance assessment.

If the participant discontinues study treatment due to an adverse event, the evaluation of the adverse event should be at least until it disappears or improves.

5.7.4 On the 28th day after the last dose of thalidomide

For female participants with fertility, a pregnancy test is required on the 28th day after the last dose of thalidomide (If the inspection has been carried out within 7 days, it is not necessary to repeat the test). If the patient cannot come to the research center for testing, it can also be tested at the nearest medical institution. If it is positive, the researcher needs to be reported and treated as Serious Adverse Events (SAE). The results are recorded in the CRF “Unplanned Visits”.

5.8 Study completion assessment

1. Study completion assessment

For all randomized participants, “Study completion assessment” should be conducted as soon as possible after the study treatment or when final analysis is required. Evaluation items include:

1) Those who have not received study treatment need to record the reasons for not receiving study treatment;

2) At the end of the study treatment, record the reason for the end of the treatment;

3) Summarize the efficacy and summarize the effective and ineffective events.

2. Response criteria

Response criteria were defined in terms of an elevation in Hb level and the need for transfusions as follows :

1) Excellent response: an elevation in the total Hb level ≥ 20 g/L, and freedom from blood transfusion for at least 6 weeks;

2) Good response: an elevation in the total Hb level between 10 g/L and 20 g/L, or an elevation in Hb level < 10 g/L with Hb > 70 g/L, and freedom from blood transfusion for at least 6 weeks;

3) No response: an elevation in the total Hb level <10 g/L and continued transfusion-dependence.

The blood transfusion standard is as follows: Hb < 70 g/L, or Hb > 70 g/L with cardiac function reaching grade III or above (see Annex 2 for the New York Heart Association (NYHA) Heart Disease Grading Standard).

The transfusion volume standard is as follows: patients complete a routine blood examination every 14 days and are transfused with leukocyte-poor red blood cells at 0.5 units/10 kg when their Hb level is < 70 g/L. If a patient receives a transfusion between day 78 and day 85 during the treatment period due to unforeseen circumstances, the timing of the efficacy evaluation will be delayed by 7 days from the day of transfusion for that particular case.

3. The following situations require "research completion assessment"

1) Those who did not receive study treatment within 2 weeks after randomization were deemed to have withdrawn from the study, and a "study completion assessment" was conducted to record the reasons for not receiving study treatment;

2) Participants who discontinued study treatment due to death, loss of follow-up, or participants withdrew from the study shall be evaluated for "study completion";

3) Participants who started a new treatment plan to terminate study treatment, conducted a "end of treatment" visit, and completed a "study completion assessment";

4) Those who discontinue study treatment due to serious adverse reactions or other reasons;

5) By the time of the final analysis, participants who have not completed the study treatment will need to conduct a "study completion assessment" and treatment may continue.

4. The participant may end treatment for one of the following reasons:

1) Disease progression;

2) Intolerable adverse events;

3) Start a new treatment;

4) Death;

5) Lost follow-up visit;

6) Participant withdrew from the trial;

7) The investigator judges that it is not suitable to continue the study and treatment;

8) Other reasons (including participants who continue treatment after disease progression).

5. The participant may not visit for one of the following reasons:

1) Lost follow-up visit;

2) Participant withdrew from the trial;

3) Other reasons.

5.9 Final analysis time

All data generated in both groups during the first phase will be compared and analyzed when the last patient completes their first 12 weeks of follow-up. The Hb level and long-term adverse effects will be evaluated when a majority of patients completes 12 months of follow-up in the second phase.

6 Efficacy and safety evaluations

6.1 Primary end-point

Primary end-point is the change in Hb level at 12 weeks after treatment initiation.

6.2 Change in the amount of Hb

The traditional treatment of thalassemia relies exclusively on blood transfusions to maintain the patient's Hb level at a target level. After the cessation of blood transfusions for a period of time, the patient's Hb level will gradually decline. Early data supporting the present trial showed that the Hb level in those who responded to the drug will gradually increase after 1 month of treatment, and reach a steady-state Hb level after 3 months of treatment. The blood transfusion standard in the present study was set to Hb < 70 g/L, and routine blood assessments are reviewed every 14 days. When the blood transfusion standard is reached, leucoreduced-packed red blood cells are transfused according to the standard of 0.5 units/10 kg of body weight. This study was designed to fully, and in a balanced manner, reflect the basic level of Hb in the two groups of patients with thalassemia, and at the same time allow the determination of the therapeutic effect by analyzing the difference between these two groups in terms of the amount of Hb after treatment.

6.3 Secondary end-points

At the end of follow-up, the blood transfusion volume, ELS, hemolysis response indicators (total bilirubin, indirect bilirubin, LDH, reticulocyte count, and peripheral nucleated red blood cell count), and change in Hb F level will be compared between groups, and a safety evaluation will be conducted.

6.3.1 Transfusion volume

The effective treatment in this study will gradually negate the need for blood transfusions. In this study, the blood transfusion standard was set to Hb < 70 g/L, with routine blood tests performed once every 14 days in the enrolled patients. When the Hb level reaches < 70 g/L, patients receive 0.5 units/10 kg of body weight, infused with leucoreduced-packed red blood cells. From day 78 to day 85 after treatment initiation, if a patient receives a transfusion due to an unexpected situation, the timing of the efficacy evaluation is delayed by 7 days from the day of transfusion for that particular case. The therapeutic effect will be judged by statistically comparing the difference in blood transfusion volume between the two groups after treatment

6.3.2 Red blood cell life measurement

Thalassemia is a hemolytic anemia, and the lifespan of red blood cells is significantly shorter than normal. Early studies have shown that thalidomide can induce the γ-globin gene to produce high levels of Hb F via different epigenetic and molecular mechanisms, thereby constructing a new peptide chain balance structure and reducing red blood cell destruction. The difference in the red blood cell life between the two groups will be used to judge the therapeutic effect.

6.3.3 Hemolysis indicators

Thalassemia is a hemolytic anemia, and group differences in the changes in hemolysis indicators (including total bilirubin, indirect bilirubin, LDH, reticulocyte count, and nucleated red blood cell count) are considered to indirectly reflect treatment efficacy.

6.3.4 Changes in the Hb F level

Early studies have shown that thalidomide can induce the γ-globin gene to produce high levels of Hb F via different epigenetics and molecular mechanisms. Thalidomide is a Hb F inducer; thus, the change in Hb F level from before to after treatment can be used as an auxiliary judgment of efficacy.

6.3.5 Safety evaluation

The safety assessment includes the incidence of adverse events, the incidence of abnormal values on clinical laboratory tests, and other safety data (such as electrocardiograms, vital signs, special examinations, etc.). Safety data, collected from the initiation the study treatment to 28 days after the end of treatment, will be analyzed. However, if a participant discontinues the study treatment due to adverse events, the data collected until the adverse event disappears or improves will be analyzed.

6.4 Exploratory end-points

Exploratory end-points refer to the observation of clinical features and genetic background related to efficacy. The effect of thalidomide in the treatment of thalassemia is considered as related to the induction of Hb F; however, its specific target gene has not been clarified in clinical trials. Therefore, we plan to conduct related gene and RNA-sequencing tests, comparing gene expression and activity before and after treatment. Additionally, we plan to explore the clinical features and genetic background related to efficacy.

6.5 Evaluation of efficacy

Testing within 7 days before the first treatment is required to establish the baseline values for

the evaluating of efficacy. Thus, the testing required for response evaluation during the treatment period must be performed ≤ 7 days before study treatment initiation and must not exceed the study treatment window.

6.5.1 Evaluation parameters

During the trial, the effectiveness of the study treatment will be evaluated using the following parameters:

1. Hb level;
2. Blood transfusion units, frequency;
3. Change in bilirubin and LDH values;
4. Reticulocyte and nucleated red blood cell counts;
5. Red blood cell lifespan;
6. Hb F electrophoresis level;
7. Clinical characteristics and genetic background related to efficacy.

6.5.2 "A sufficient evaluation of efficacy"

The study protocol stipulates that "a sufficient evaluation of efficacy" includes at least the evaluation of the Hb volume, blood transfusion volume, and Hb F value. Therefore, the evaluation at the third month after treatment initiation must comprise a full evaluation.

6.6 Adverse events

6.6.1 Adverse event definition

In this study, adverse events refers to any adverse and unexpected medical events or signs (including abnormal laboratory results), and symptoms or diseases (or an aggravation of any previously existing condition) that occur in participants after receiving study treatment, but do not necessarily have a causal relationship with the study drug. According to the requirements of the Good Clinical Practice (GCP), regardless of whether there is a causal relationship with the test drug, the researcher must record all adverse events in the original records and enter it into the CRF.

6.6.2 Records of adverse events

All participants in the trial must be evaluated for adverse events from the initiation of study treatment to at least 28 days after the last dose of the study drug. However, if a participant discontinues study treatment due to an adverse event, the participant should be evaluated until the

adverse event disappears and participant's condition is improved.

During each trial visit, the participant is queried in a non-directional manner to determine whether an adverse event occurred. This information can also be obtained through the participant's active report or physical examination, laboratory examination, or other evaluations during the visit. Participants are instructed that if any adverse symptoms or adverse reactions occur during the trial, the investigator must be notified. Within a few weeks of a participant's inability to be evaluated in the research center, the participant is contacted by phone, as necessary to evaluate the course and nature of the adverse event; each adverse event will be evaluated as fully as possible to determine the following characteristics:

1. Type of adverse event;
2. Severity classification;
3. Start and end dates;
4. Relationship with study treatment;
5. Prognosis (healed, improved, not improved, with sequelae, death, or unknown);
6. Whether severe adverse events (SAE) and SAE conditions occurred (death, hospitalization, extended hospital stays, disability, dysfunction, congenital malformations, or others);
7. Relationship with test supplies;
8. Measures taken (medication continued, dose reduced, medicine suspended for __ days and then resumed, medication stopped, or symptomatic treatment received).

The progression of anemia in an ongoing clinical trial and hospitalization due to anemia alone are not considered as adverse events. However, isolated abnormal laboratory test values and test results that have clinical significance or require medical intervention are considered as adverse events and will be recorded in the CRF "Adverse Event Summary Table".

If possible, a specific medical diagnosis is reported, rather than isolated laboratory abnormalities, symptoms, or signs. However, laboratory or examination abnormalities, symptoms, and signs must be recorded in the original records. If the abnormality is not part of the medical diagnosis, the laboratory abnormality, symptom, or sign is also recorded in the CRF "Adverse Event Summary Form".

Regardless of the level of classification, as long as the event meets SAE standards, it must be recorded and reported as a SAE.

6.6.3 Adverse event classification

The intensity of all adverse events will be graded according to the National Cancer Institute (NCI) Partial Common Toxicity Criteria (CTC) Adverse Event Classification CTC AE3.0 (Annex 3) Grade 5 scale and recorded in the CRF. If the CTC AE classification does not exist for an adverse event, it will be graded according to the CTC AE 4.03 specific grade description, as described below.

CTC AE classification	Definition
Grade 1	Mild; asymptomatic or mildsymptoms; clinical or diagnostic observations only; intervention not indicated
Grade 2	Moderate: minimal, local or noninvasive intervention indicated; limiting age-appropriate instrumental ADL *
Grade 3	Severe or medically significant but not immediately life-threatening; hospitalization or prolongation ofhospitalization indicated; disabling; limiting self care ADL **
Grade 4	Life-threatening consequences; urgent intervention indicated.
Grade 5	Death related to AE

Activities of Daily Living (ADL): * Instrumental ADL refer to the preparation of meals, shopping for groceries or clothes, using the telephone, managing money, etc. ** Self-care ADL refer to bathing, dressing and undressing, feeding oneself, using the toilet, taking medications, and a lack of bedridden status.

6.6.4 The causal relationship between treatment/experimental drugs and adverse events

Refer to Section 5.4.1 for the definitions of the study treatment and experimental drugs. The evaluation of the causal relationship between treatment/experimental drugs and adverse events follows the guidelines of the National “Administrative Measures on the Report and Monitoring of Adverse Drug Reactions”, and will be conducted according to the five criteria of adverse reaction/event analysis. According to these criteria, the relevance will be classified into 6 levels: positive, likely, possible, unrelated, to be evaluated, and unable to be evaluated (see Annex 5).

If the treatment or a certain trial drug is considered as positively associated with the adverse event (which includes possibly related and unable to be evaluated classification), the presence of a

causal relationship can be determined as “yes”; such adverse events can be considered as research treatment-related or trial drug-related adverse events. If an adverse event is considered as possibly or absolutely unrelated to the research treatment or a trial drug, the presence of a causal relationship can be determined as “no”; these adverse events can be considered as unrelated to the research treatment or the adverse event of the trial drug.

If an adverse event related to the trial drug occurs, it must be considered whether the participant’s medical history could cause the adverse event or increase the participant’s susceptibility.

6.6.5 Serious adverse events

In order to ensure the safety of the participants, regardless of whether a causal relationship exists, any serious adverse events that occur within 28 days after the initiation of study treatment to the end of study treatment must be reported to the sponsor immediately (within 24 hours). Serious adverse events refer to the following adverse events:

1. Causation of death;
2. Life-threatening conditions;
3. Causation of congenital malformations;
4. Causation of significant or permanent human disability or organ damage;
5. Causation of hospitalization or prolonged hospitalization.

The following admission treatment events are not considered as SAE:

1. Standard treatment procedures associated with the dosing regimen;
2. Events that occur as part of the monitoring of routine treatment or research indications, that are associated with any aggravation;
3. Platelet transfusions as a routine treatment indicator;
4. Events due to the testing plan/examinations that are related to the disease (e.g. surgery, scanning, endoscopy, bone marrow aspiration, and other laboratory tests);
5. Hospitalization or prolonged hospitalization due to a technical operation or social reasons
6. Events due to an existing admission treatment plan;
7. Selective treatment of existing diseases not related to the indications of this trial;
8. Emergency treatment or observation leading to hospitalization, unless other severity criteria are met;

9. Additionally, hospitalization simply due to anemia progression is not considered a serious adverse event.

6.6.6 Handling of adverse events

Once an adverse event is found, follow-up must be conducted until it disappears or improves. Appropriate measures must be taken for all adverse events, such as continued medication administration, dose reduction, recovery after drug suspension, withdrawal of medication, symptomatic treatment, and the termination of research treatment.

If a female participant or female partner of a male participant becomes pregnant or pregnancy is suspected (including a positive pregnancy test, without consideration of age or disease status at the time of the pregnancy test or within 28 days after the last administration of medication), the following treatment and reporting plan must be implemented (see Annex 3: Emergency Plan for Pregnancy Events):

1. When pregnancy is confirmed, pregnancy is suspected, or a pregnancy test is positive, the researcher must immediately report this SAE to the sponsor and the research team leader unit, as well as the Chinese Food and Drug Administration (CFDA), in writing (see the table for the specific list and contact details);

2. Participants must be immediately instructed to stop using the test drugs and return any unused drugs to the investigator. Male participants must ensure complete abstinence before they can continue to receive treatment;

3. It is recommended that the pregnant participant or the female sexual partner of the male participant terminate the pregnancy;

4. The pregnant woman should be followed up until the termination of pregnancy, to determine the outcome, including spontaneous or voluntary termination of pregnancy, details regarding childbirth, whether there birth defects or maternal and/or neonatal comorbidities were present. In addition, the pregnancy results must be reported in the initial SAEs follow-up supplementary report.

1) If the consequences of this pregnancy meet the SAE classification criteria, it should be reported immediately, including spontaneous or artificial abortion (any congenital malformations of fetuses detected must be recorded), stillbirth, and neonatal death. For congenital malformations (including congenital malformations in aborted fetuses), the investigator must immediately report

this SAE within 24 hours, according to planned procedures (in writing to the sponsor and the research team leader unit and CFDA);

2) If there are surviving “normal” babies, the investigator should report to the sponsor and the research team leader unit and CFDA in writing within 24 hours of learning of the event;

3) All neonatal deaths occurring within 28 days of birth, regardless of whether there a causal relationship with the test drug exists, should be reported as an SAE;

4) Any infant death after 28 days of birth, if the investigator believes that it is related to intrauterine exposure to the test drug, should also be reported in writing to the sponsor and the research team leader unit and CFDA within 24 hours of learning of the event.

If it is discovered that the female participant or the female sexual partner of the male participant is not pregnant, the investigator and the sponsor must decide whether the participant should continue to participate in the trial.

6.6.7 Reporting of serious adverse events

If a SAE occurs during the trial, the research center must immediately take measures to protect the safety of the participant, and must immediately (within 24 hours) report the SAE to the sponsor and the research team leader unit and CFDA in the form of a book; the researcher must record the occurrence time, duration, measures, and outcomes of SAEs in the “Serious adverse event report form”. The researcher must sign and date this report.

Serious adverse event reporting unit

**Reporting unit**	**Contact person**	**Contact number**
CFDA Safety Supervision Department Drug Research and Supervision Department	—	010-68313344-1013
Safety Supervision Department, Food and Drug Administration of Wuzhou City, Guangxi Zhuang Autonomous Region	—	0774-5835811
Changzhou Pharmaceutical Co., Ltd.	Liu Zheng	15295230525

6.6.8 Laboratory abnormalities

When abnormal laboratory tests are not accompanied by clinical symptoms and signs, they are not recorded as adverse events; such changes will be considered in the data analysis. However, if the investigator believes that the necessary treatment should be given because of the degree of the laboratory test abnormality, the laboratory examination value must be recorded as an adverse event, along with information regarding the accompanying treatment. All laboratory tests should be followed up until remission or stability.

7 Implementation of the blindness, blindness breaking and unblinding

7.1 Implementation of the blindness

Using the RANDBETWEEN function of Microsoft Excel 2016, two columns of random codes corresponding, respectively, to the two groups of participants (a total of 100 cases); namely, a treatment allocation table with serial numbers of 1-100, is created. In accordance with the requirements of a double-blinded and single-simulated clinical trial, staff members not involved in the study are responsible for numbering the drugs. A detailed document is used to record the entire blinding process, and the results of this process are preserved at Wuzhou Gongren Hospital in Guangxi Province.

Preparing the medicine: the experimental drugs (thalidomide and placebo) are blindly packed under the supervision of experts in biostatistics according to the random coding of drugs.

7.2 Blindness breaking of individual cases in emergency circumstances

Partners, lead researchers, and sponsors will jointly determine whether to take an urgent break in treatment in cases of the following three emergencies:

1. The patient requires emergency aid and treatment;

2. Cessation of all experimental drugs does not solve the circumstances of the emergency;

3. The emergency measures taken in breaking cases are significantly different from those in non-breaking cases.

Partners, lead researchers, and sponsors will objectively try to avoid any exigent unblinding, unless the emergency break in treatment would affect the measures taken for rescue treatment. Once the blindness is broken, the case will be regarded as a drop-out one. The case should be included in the safety analysis if there is an adverse event, and be included in the efficacy analysis if there is data on curative effect before the blindness breaking.

7.3 Regulations of unblinding

The inspectors should regularly check the implementation status from the start of the trial. Deal with the emergency circumstance and report it in time when there is a necessity for unblinding. The database is locked after the data are questioned and blindly reviewed. The main researchers, the sponsors, the professional staff in biostatistics and the people concerned preserving the results of blindness will convene an unblinding conference officially. Distinguish that the medication code of each participant belongs to test group or control group, and then hand

over the results of blindness to the experts in biostatistics for statistical analysis. Finally submit the statistical analysis when completed to the unit in charge of the clinical trial to write a summary report. During the trial process, the double-blinded trial is deemed to fail if the rate of unblinding exceeds 20%.

8 Data processing and statistical analysis

The biostatistics department of CRO company designated by the sponsor is responsible for statistical analysis of the data obtained in this trial.

8.1 Data processing

8.1.1 Traceability of data, completion and transferring of Case Report Form (CRF)

Wuzhou Gongren Hospital of Guangxi Province are in charge of the data management. It is necessary to ensure the authenticity, integrity and privacy of the clinical trial data. And the process of data management should comply with CIH, GCP, FDA 21, CFR, Part 11 and other stipulations.

Ensure the traceability of clinical trial data.

Case Report Form (CRF) shall be filled out truthfully by researchers or the staff designated by researchers with reference to the original case and shall not be altered at will. Perform a standard operation on the basis of CRF filling instructions and sign the mender's name and the modified date if there is a necessity of alteration. Researchers of each center should verify the information of each CRF to ensure its accuracy and authenticity, and then sign the name and date.

Researchers and data management staff are supposed to check the reclaimed CRF after it is completed, and sign the CRF freight note after confirmation.

8.1.2 Design and establishment of Database

The database is established by the medical data department and should comply with the requirements of FDA 21, CFR and Part 11. The database should manage the data traces including system login, data entry, modification, and deletion. The database should be established using the CDISC standard as far as possible.

8.1.3 Data entry and modification

The data administrators designated by the statistical unit are in charge of the data entry and management. Data administrators apply a specialized data management system to compile the data entry procedure for data entry and management. In order to ensure the accuracy of the data, two data administrators should independently conduct the data entry for two times each and proofread.

For questions existing in the CRF, data administrators will generate a form of data ready for query (DRQ) and send it to researchers through clinical inspectors. Researchers should answer the questions and return it as soon as possible. Then data administrators will modify, confirm and import the data according to researchers' answer. DRQ can be issued again if necessary.

8.1.4 Data verification

Data verification includes computerized programmed verification (Edit Check), manual verification and data verification conference. The inconsistent data (Data Query) found in the process of verification should be corrected in time, or the data department should issue a discrepancy report and submit it to researchers for confirmation before modification.

8.1.5 Data locking

After reviewing and confirming the correctness of the established database, the main researchers, the sponsors, and the staff in statistical analysis jointly lock the data. Generally speaking, the locked data file will not be changed. The problems found after data locking should be strictly modified in accordance with the process of unlocking and relocking.

8.2 Statistical analysis plan

8.2.1 Sample size

Based on our previous study and literature review, the Hb level in patients receiving placebo intervention is assumed to be 60 g/L and to not improve with intervention. In contrast, the Hb level in the thalidomide group is expected to rise to at least 72 g/L (i.e., at least 12 g/L higher than that in the control group). The standard deviation is presumed to be 20 g/L after 12 weeks of treatment.

Given that the ratio of patients assigned to the two groups is 1:1, the comparison in Hb levels is processed as an independent-samples t-test, with $P < 0.05$ considered statistically significant. Setting α to 0.05 and β to 0.2 in a two-sided test, and under the assumption that 10% of the patients would withdraw or drop out, we estimate that enrollment of approximately 100 patients is required, with 50 patients in each group.

8.2.2 Datasets of statistical analysis

Datasets are defined as follows:

1) The full analysis set (FAS) will comprise patients who take the drug at least once after enrollment, without missing data for the main evaluation indicator. Cases that lack baseline data for the evaluation indicator will be eliminated from the FAS;

2) The per protocol set (PPS) is based on the FAS, and will comprise patients who satisfy the inclusion and exclusion criteria, have valid baseline values and good compliance, and rigorously follow the clinical trial protocol (e.g., non-use of thalidomide, hydroxyurea, decitabine, azacitidine,

butyratesor, erythropoietin, or yisuishengxue granules);

3) The safety set (SS) will comprise patients who take the drug at least once after enrollment and receive treatment for at least 1 month.

Both the FAS and PPS will used in the efficacy analysis; however, the FAS will be regarded as the primary index. Additionally, the SS will used in the safety analysis.

8.2.3 Efficacy and safety indicators

The Hb level is considered the primary indicator in this study, and the Hb values at different time points, as well as the elevation in Hb level, are the main criteria of efficacy. The secondary indicators include reductions in the transfusion burden, Hb F level, ELS, hemolytic markers, and adverse effects. After unblinding, differences between the two groups in the above indicators will be evaluated to determine the efficacy of thalidomide in treating transfusion-dependent *β*-thalassemia, and its safety will be evaluated in terms of long-term adverse events.

8.2.4 Statistical analysis

Data are entered by two staff members (two times each) into a database created using Epidata, and are locked after a lack of errors is confirmed. Data were analyzed according to participants' randomized treatment assignments, regardless of their subsequent medication status (intention-to-treat). The primary and secondary outcomes were compared between the experimental and control groups using the Student's t-test at a 2-sided α level of 5%, without correction for multiple comparisons. Continuous variables with a normal distribution will be expressed as the mean ± standard deviation (SD), and variables with a non-normal distribution will be expressed as the median (inter-quartile range), and the Mann-Whitney U test will be used to compare groups. To further compare the dynamic Hb levels and transfusion volume between the two randomized arms, we constructed a linear mixed-effects regression model in which the repeated measures were the dependent variables and intervention group was the independent variables. Data analyses were performed using IBM SPSS Statistics 26.0 (Chicago, IL, USA). All statistical tests were two-sided and *P* values less than 0.05 were considered statistically significant.

8.2.5 Significance level

A hypothesis test is performed with the significance level of 0.05 (two-sided) for the efficacy indicator.

8.2.6 Treatment compliance

At the dose level specified in the protocol (including the reduced dose level), thalidomide or placebo should be administrated for at least 12 week. The situation that the days for participants taking thalidomide account for at least 80% of the expected days (i.e. the actual days of taking tthalidomide/the expected days for the course of treatment) is regarded as “completing a course of treatment”. Other situations beyond the following one are all defined as “poor treatment compliance” rather than “completing a course of treatment”, whereas it should be counted when considering how many courses of treatment the participants have received.

8.2.7 Missing values and outliers

CRF must be filled in clearly and there should be no missing data in principle, especially significant indicators (for example, the main efficacy and safety indicators). The basic data in CRF, such as gender, dates of birth, enrollment, and observation, shall not be missing. The negative result observed in the trial, the testing result of zero, and the result not attained should all be represented with the corresponding symbols. Fill in all the blanks with no vacancy to distinguish them from missing data.

The outlier handling should be considered from the majors in medicine and statistics, especially the medical professional knowledge. The processing of outliers should be performed during blindness inspection.

8.2.8 Incomplete date

It is necessary to record the detailed dates of visits, including year, month and day. If the day is not recorded, the 15th day of the month shall be counted.

8.2.9 General principles of statistical analysis

All statistical tests are two-sided unless otherwise specified, and *P* values less than 0.05 are considered statistically significant.

Calculate the mean, standard deviation, median, minimum, and maximum for description of quantitative indicators, and count the number of cases and calculate the percentage of each category for description of categorical variables.

Independent-samples *t*-test, Chi-square test or rank sum test are used for the homogeneous comparison among groups.

8.2.10 Comparability/equilibrium analysis of demographic data and baseline characteristics

Comparability/equilibrium analysis, including demographic characteristics, classification of disease, course of disease, ECOG score, previous transfusion volume, and previous dose of iron-removing preparations, is mainly used to judge the effectiveness of randomization. If the randomization is good, there is no need to consider the influence of other factors when doing statistical analysis; otherwise, an extra correction analysis of the influencing factors is required.

8.2.11 Classification of participants

The frequency and percentage of the treatment period and follow-up period will be used to summarize the classification of the participants (analyze the enrolled population, population removed from the trial early and main reasons for their withdrawals). Summarize the circumstances of participants and use a frequency table to record the deviation.

8.2.12 The experimental drug

Provide the statistical data on the dose of experimental drug of the safetey population (including mean, median, standard deviation and range). Also provide administration information about daily average dose, accumulated dose, dose intensity and relative dose intensity.

8.2.13 Concomitant treatment

All the drug combinations and non-drug treatments recorded during the clinical trial will be summarized in a frequency table. Concomitant treatment/drug combination include all medicines (excluding the experimental drug) used from 14 days before randomization to the end of the trial when doing statistical analysis.

8.2.14 Efficacy analysis

The efficacy analysis will be based on FAS and PPS. The primary endpoint of the change in Hb level and secondary endpoints of the transfusion burden, erythrocyte life span, hemolytic markers and the change in Hb F level will be evaluated by the independent assessment committee.

8.2.15 Missing data of efficacy analysis

In order to further evaluate the impact of missing data on the efficacy analysis and to summarize the compliance of the study follow-up visit, the following information will be provided according to the different treatments:

1. Proportion of participants who miss the visit or have incomplete data collection during the follow-up visit;

2. Number of evaluations with missing visit and incomplete data collection.

If any necessary data on efficacy evaluation is missed out in the screening period or the first 3 months during treatment, the participant is considered to have incomplete data at the time of this follow-up visit.

8.2.16 Safety analysis

All participants who have received treatment will be included in the safety analysis.

Classify the adverse events with the MedDRA classification system and grade the severity of toxic reactions according to NCI-CTCAE version 3.0. Summarize the following adverse events: leading to death or termination of the treatment, categorized as Grade 3 or 4 by NCI-CTCAE, related to the experimental drug CPT and serious adverse events, respectively.

The frequency of adverse events will be listed according to the human organs and preferred terminology. In the case-by-case analysis, participants who have the same certain adverse event more than once are counted only once.

Provede descriptive statistics (average, median, minimum and maximum standard deviation) for data of vital signs and weight.

List all the completed examination items and descriptive variables (based on the range of normal value and judgment of researchers on clinical significance) in the form of a crosstab before and after administration for laboratory examinations and electrocardiogram examinations. And list the examination items with abnormal values and clinical significance.

The safety evaluation is based on the analysis of the data from the start of the treatment to 28 days after the end of the treatment. But if the participant terminates the treatment due to adverse events, the safety should be evaluated when the adverse event disappears or improves and the data should also be included in the safety analysis.

9 Independent Assessment Committee (IAC)

The trial will specifically establish an Independent Assessment Committee (IAC), including at least 3 doctors with professional knowledge and experience in the diagnosis and management of thalassemia, but they do not directly participate in the execution of the trial.

IAC will verify the efficacy evaluation date and disease progression and evaluate the previous treatment schemes. The data verified by IAC will be used for final analysis and the evaluation of these data will be included in the research report.

The verification conducted by the IAC is independent of the researchers' evaluation of disease remission during the trial to reasonably manage the participants. The independent verification needs to be completed blindly.

10 Ethics

The main researchers have the responsibility to ensure that the conduct of this trial will fully comply with the Declaration of Helsinki and the Chinese Criterions for the Quality Control of Clinical Trial of drugs and other relevant regulations.

10.1 Ethics committee

The main researchers and sponsors should ensure that the trial meets GCP requirements and is reviewed and approved by a qualified ethics committee. Before the start of the trial, the trial protocol, informed consent and other necessary materials should be submitted to the ethics committee for review. The sponsors can not provide trial drugs until receiving the approval of the ethics committee. The main researchers are responsible to report the progress of the trial, any modification of the trial protocol and serious adverse events occurring during the trial to the ethics committee.

10.2 Informed consent

See Annex 6.

10.3 Participants' information privacy

The researchers have a duty to maintain the anonymity of the participants. Only the code can be used to identify the participant rather than the real name in CRF or other documents. Researchers must properly preserve the screening table with the personal information of the participant, which is used only for real-time data verification.

11 Clinical trial management

11.1 Scheme formulation and implementation

The design and formulation of the clinical trial scheme are jointly completed by the sponsors and researchers. And the trial scheme and contract on behalf of the agreement on execution are signed.

Any individual participating in the trial shall not violate the scheme. If there is a violation of the scheme, write out any modification related to the scheme and report it to the ethics committee for approval before execution.

11.2 Experimental training

Ensure that all relevant staff involved in the trial receive the necessary training organized by the sponsors. Convene a conference towards all researchers to make them familiarize with the research protocol and the distribution procedure of the trial drug before starting the trial in earnest. Simultaneously hold a necessary training on knowledge of GCP.

11.3 Supervision and inspection of the trial

The trial is supervised by qualified supervisors who are approved and sent by the sponsors to ensure the safety of the participants and that the trial are carried out in strict accordance with the requirements of the scheme. The supervisors will visit the research institution during the trial and complete the following tasks (including but not limited to):

1. Confirm that the safety and rights of the participants are fully protected;

2. Confirm that the trial is carried out in full accordance with the requirements of the research scheme, GCP and other relevant regulations;

3. Confirm that informed consent is completed in strict accordance with the requirements and the consent form is signed by the participants themselves;

4. Verify whether the adverse events occurred are true and report them correctly and timely;

5. Check the CRF and original data to confirm that if the CRF is filled accurately and no blank item exists;

6. Confirm that various documents related to the trial have been properly archived and saved.

When independent ethics committees (EC), regulatory agencies (such as CFDA) and the company authorized representatives conduct a supervision or inspection, researchers are required to allow them to be in direct contact with the sites of the trial, the original records, and the

corresponding supporting records of the participants' participation in the trial. The researchers should be on the scene as much as possible during the process of supervision and/or inspection.When any regulatory agency contacts with researchers for inspection, researchers should immediately inform Changzhou Pharmaceutical Factory.

11.4 Drug administration

Thalidomide (and placebo) for this clinical trial are provided by the sponsors. Researchers should bring the CFDA approval, the copy of the drug's certificate of qualification, ethics committee approval and other relevant materials to the office of the clinical drug trials agency for registration procedures before the trial, and store the drug in a special place as required. Drugs should be strictly managed by the specially-assigned person in accordance with the drug administration regulations and the receiving, distributing and storing of the drugs must be recorded in detail. The experimental drug is only used for this trial and can not be used in any other trials. Check the amount of the drugs carefully and destroy the remaining drugs in time after the trial .

11.5 Information preservation

The researchers should preserve the whole original records of all participants in a specific and safe place in case of the sponsor's supervision. According to the requirements of GCP, all original records and CRFs are supposed to be kept for 5 years from the end of the trial. Besides, the sponsors should also preserve the materials for future reference.

12 Publication of the research results

The results of this trial can be published in medical publications and journals, or be used for teaching purposes. Since the trial is defined as a multi-center trial, the articles published on the basis of the results of this trial should include the contributions of all researchers and sponsors of each sub-center. And the explanation of contributions should be listed in the published article. Decide the byline according to relevant stipulations and actual contributions when publishing a summary report.

The researchers should agree not to conduct publication or distribution activities without any drafts of sponsors' reviewing or commenting (including but not limited to submitting research reports to relevant government departments, publishing the trial and research results on the public registration network, namely public registry). For this purpose, drafts of publications or completed publications should be submitted to the sponsors within 4 weeks prior to the expected release date.

13 Early termination of a single center

The main researchers and sponsors have the right to prohibit a certain sub-center from participating in the following study at any time during the clinical trial with reasonable cause. Possible reasons for termination include (but are not limited to):

1) The quantity or quality of the enrollment is too low;

2) Inaccurate or incomplete of data collection;

3) Falsify records;

4) Failure to comply with the clinical trial protocol.

References

[1] Padate B, Bain BJ, de la Fuente J. Ineffective hemopoietic in β-thalasswmia major visualised[J]. Am J Hematol, 2001, 86(4): 372.

[2] Taher A, Vichinsky E, Musallam K, Cappellini MD, et al. Guidelines for the management of non transfusion dependent thalassaemia (NTDT). Nicosia (Cyprus): Thalassaemia International Federation, 2013.

[3] Chand G, Chowdhury V, Manchanda A, Singh S. Deferiprone-induced arthropathy in thalassemia: MRI findings in a case[J]. Indian J Radiol Imaging, 2009, 19(2): 155-157.

[4] Grady RW, Galanello R, Randolph RE, et al. Toward optimizing the use of deferasirox: potential benefits of combined use with deferoxamine[J]. Haematologic, 2013, 98(1): 129-135.

[5] Weatherall DJ. The thalassemias: disorders of globin synthesis. In: Kaushansky K, Prchal JT, Press OW, et al., eds. Williams Hematology, 9th ed. New York: McGraw-Hill Education; 2016, 725-748.

[6] Stewart AK. How thalidomide works against cancer. Science, 2014, 343(6168): 256-257.

[7] Aguilar-Lopez LB, Delgado-Lamas JL, Rubio-Jurado B, et al. Thalidomide therapy in a patient with thalassemia major[J]. Blood Cells Mol Dis, 2008, 41(1): 136-137.

[8] Jalali FMA, Dehghani FA, Hajizamani S, et al. Thalidomide is more efficient than sodium butyrate in enhancing GATA-1 and EKLF gene expression in erythroid progenitors derived from HSCs with β-globin gene mutation[J]. Int J Hematol Oncol Stem Cell Res, 2016, 10(1): 37-41.

[9] Aerbajinai W, Zhu J, Gao Z, et al. Thalidomide induces gamma-globin gene expression through increased reactive oxygen species-mediated p38 MAPK signaling and histone H4 acetylation in adult erythropoiesis[J]. Blood, 2007, 110(8): 136-137.

[10] Chen J, Zhu W, Cai N, et al. Thalidomide induces haematologic responses in patients with β-thalassaemia. Eur J Haematol, 2017, 99(5): 437-441.

[11] Bayanzay K, Khan R. Meta-analysis on effectiveness of hydroxyurea to treat transfusion-dependent β-thalassemia[J]. Hematology, 2014, 20(8): 469-476.

[12] El-Beshlawy A, El-Ghamrawy M, EL-Ela MA, et al. Response to hydroxycarbamide in pediatric β-thalassemia intermedia: 8 years' follow-up in Egypt. Ann Hematol, 2014, 93(12):

2045-2050.

[13] Kosaryan M, Zafari M, Alipur A, Hedayatizadeh-Omran A. The effect and side effect of hydroxyurea therapy on patients with *β*-thalassemia: a systematic review to December 2012[J]. Hemoglobin, 2014, 38(4): 262-271.

[14] Olivieri NF, Sauntharajah Y, Thayalasuthan V, et al. A pilot study of subcutaneous decitabine in *β*-thalassemia intermedia[J]. Blood, 2011, 118(10): 2708-2711.

[15] Ali DF, Seyed AH, Mohammad S, et al. Evaluation of novel fetal hemoglobin inducer drugs in treatment of *β*-hemoglobinopathy disorders[J]. Int J Hematol Oncol Stem Cell Res, 2013, 7(3): 47-54.

[16] Grzasko N, Chocholska S, Goracy A, et al. Thalidomide can promote erythropoiesis by induction of STAT5 and repression of external pathway of apoptosis resulting in increased expression of GATA-1 transcription factor[J]. Pharmacological Reports, 2015, 67(6): 1193-1200.

Annex 1 ECOG Performance Status

0	Fully active, able to carry on all pre-disease performance without restriction
1	Restricted in physically strenuous activity but ambulatory and able to carry out work of a light or sedentary nature, e.g., light house work, office work
2	Ambulatory and capable of all selfcare but unable to carry out any work activities; up and about more than 50% of waking hours
3	Capable of only limited selfcare; confined to bed or chair more than 50% of waking hours
4	Completely disabled; cannot carry on any selfcare; totally confined to bed or chair
5	Dead

Oken M, Creech R, Tormey D, et al. Toxicity and response criteria of the Eastern Cooperative Oncology Group. Am J Clin Oncol. 1982; 5: 649-655.

Annex 2 New York Heart Association (NYHA) Functional Classification

Class	Patient Symptoms
I	No limitation of physical activity. Ordinary physical activity does not cause undue fatigue, palpitation, dyspnea (shortness of breath).
II	Slight limitation of physical activity. Comfortable at rest. Ordinary physical activity results in fatigue, palpitation, dyspnea (shortness of breath).
III	Marked limitation of physical activity. Comfortable at rest. Less than ordinary activity causes fatigue, palpitation, or dyspnea.
IV	Unable to carry on any physical activity without discomfort. Symptoms of heart failure at rest. If any physical activity is undertaken, discomfort increases.
**Class**	**Objective Assessment**
A	No objective evidence of cardiovascular disease. No symptoms and no limitation in ordinary physical activity.
B	Objective evidence of minimal cardiovascular disease. Mild symptoms and slight limitation during ordinary activity. Comfortable at rest.
C	Objective evidence of moderately severe cardiovascular disease. Marked limitation in activity due to symptoms, even during less-than-ordinary activity. Comfortable only at rest.
D	Objective evidence of severe cardiovascular disease. Severe limitations. Experiences symptoms even while at rest.

Adapted from Dolgin M, Association NYH, Fox AC, Gorlin R, Levin RI, New York Heart Association. Criteria Committee. Nomenclature and criteria for diagnosis of diseases of the heart and great vessels. 9th ed. Boston, MA: Lippincott Williams and Wilkins; March 1, 1994.
Original source: Criteria Committee, New York Heart Association , Inc. Diseases of the Heart and Blood Vessels. Nomenclature and Criteria for diagnosis, 6th edition Boston, Little, Brown and Co. 1964, p 114.

Annex 3 Common Terminology Criteria for Adverse Events v3.0 (CTCAE)

**Adverse Event**	**Grade**				
	**I**	**II**	**III**	**IV**	**V**
**Blood/bone marrow**					
Hb	<LLN - 10.0 g/dL<br><LLN - 6.2 mmol/L<br><LLN -100 g/L	<10.0 - 8.0 g/dL<br><6.2 - 4.9 mmol/L<br><100 - 80g/L	<8.0 - 6.5 g/dL<br><4.9 - 4.0 mmol/L<br><80 - 65 g/L	<6.5 g/dL<br><4.0 mmol/L<br><65 g/L	Death
Leukocytes	<LLN - 3000/mm^3<br><LLN - 3.0 x 109 /L	<3000 - 2000/mm^3<br><3.0 - 2.0 x 109 /L	<2000 - 1000/mm^3<br><2.0 - 1.0 x 109 /L	<1000/mm^3<br><1.0 x 109 /L	Death
Neutrophils	<LLN - 1500/mm^3<br><LLN - 1.5 x 109 /L	<1500 - 1000/mm^3<br><1.5 - 1.0 x 109 /L	<1000 - 500/mm^3<br><1.0 - 0.5 x 109 /L	<500/mm^3<br><0.5 x 109 /L	Death
Platelets	<LLN - 75,000/mm^3<br><LLN - 75.0 x 109 /L	<75,000 - 50,000/mm^3<br><75.0 - 50.0 x 109 /L	<50,000 - 25,000/mm^3<br><50.0 - 25.0 x 109 /L	<25,000/mm^3<br><25.0 x 109 /L	Death
Blood/Bone Marrow - Other	Mild	Moderate	Severe	Life-threatening; disabling	Death
**Coagulation**					
PTT (Partial Thromboplastin Time)	>1 - 1.5 x ULN	>1.5 - 2 x ULN	>2 x ULN	—	—
**Metabolism**					
Bilirubin	>ULN - 1.5 x ULN	>1.5 - 3.0 x ULN	>3.0 - 10.0 x ULN	>10.0 x ULN	—
ALT	>ULN - 2.5 x ULN	>2.5 - 5.0 x ULN	>5.0 - 20.0 x ULN	>20.0 x ULN	—
GGT	>ULN - 2.5 x	>2.5 - 5.0 x	>5.0 - 20.0 x	>20.0 x	—

Adverse Event	Grade				
	I	II	III	IV	V
	ULN	ULN	ULN	ULN	
Alkaline phosphatase	>ULN - 2.5 x ULN	>2.5 - 5.0 x ULN	>5.0 - 20.0 x ULN	>20.0 x ULN	—
Hypocalcemia	<LLN - 8.0 mg/dL<br><LLN - 2.0 mmol/L<br>Ionized calcium:<br><LLN - 1.0 mmol/L	<8.0 - 7.0 mg/dL<br><2.0 - 1.75 mmol/L<br>Ionized calcium:<br><1.0 - 0.9 mmol/L	<7.0 - 6.0 mg/dL<br><1.75 - 1.5 mmol/L<br>Ionized calcium:<br><0.9 - 0.8 mmol/L	<6.0 mg/dL<br><1.5 mmol/L<br>Ionized calcium:<br><0.8 mmol/L	Death
Hypokalemia	<LLN - 3.0 mmol/L	—	<3.0 - 2.5 mmol/L	<2.5 mmol/L	Death
creatinine	>ULN - 1.5 x ULN	>1.5 - 3.0 x ULN	>3.0 - 6.0 x ULN	>6.0 x ULN	Death
Proteinuria	1+ or<br>0.15 - 1.0 g/24 hrs	2+ to 3+ or<br>>1.0 - 3.5 g/24 hrs	4+ or<br>>3.5 g/24 hrs	Nephrotic syndrome	Death
Hburia	Present	—	—	—	Death
**Gastrointestinal tract**					
Stomatology	Oral mucosa erythema	Ulcer or pseudomembrane	Ulcer or pseudomembranes converge into pieces, and small damage causes bleeding	Tissue necrosis, apparent spontaneous bleeding, secondary life-threatening symptoms	Death
Anorexia	Loss of appetite without alteration in eating habits	Oral intake altered without significant weight	Associated with significant weight loss or malnutrition (e.g.,	Life-threatening consequences	Death

Adverse Event	Grade				
	I	II	III	IV	V
		loss or malnutrition; oral nutritional supplements indicated	inadequate oral caloric and/or fluid intake); IV fluids, tube feedings or TPN indicated		
Nausea	Loss of appetite without alteration in eating habits	Oral intake decreased without significant weight loss, dehydration or malnutrition; IV fluids indicated <24 hrs	Inadequate oral caloric or fluid intake; IV fluids, tube feedings, or TPN indicated ≥24 hrs	Life-threatening consequences	Death
vomiting	1 episode in 24 hrs	2 - 5 episodes in 24 hrs; IV fluids indicated <24 hrs	≥6 episodes in 24 hrs; IV fluids, or TPN indicated ≥24 hrs	Life-threatening consequences	Death
Diarrhea	Increase of <4 stools per day over baseline; mild increase in ostomy output compared to baseline	ncrease of 4 - 6 stools per day over baseline; IV fluids indicated <24hrs; moderate increase in ostomy output compared to baseline; not interfering with ADL	ncrease of ≥7 stools per day over baseline; incontinence; IV fluids ≥24 hrs; hospitalization; severe increase in ostomy output compared to baseline; interfering with ADL	Life-threatening consequences (e.g., hemodynamic collapse)	Death

Adverse Event	Grade				
	I	II	III	IV	V
Constipation	Occasional or intermittent symptoms; occasional use of stool softeners, laxatives, dietary modification, or enema	Persistent symptoms with regular use of laxatives or enemas indicated	Symptoms interfering with ADL; obstipation with manual evacuation indicated	Life-threatening consequences (e.g., obstruction, toxic megacolon)	Death
PULMONARY/UPPER RESPIRATORY					
Cough	Symptomatic, non narcotic medication only indicated	Symptomatic and narcotic medication indicated	Symptomatic and significantly interfering with sleep or ADL	—	—
ALLERGY/IMMUNOLOGY					
Allergic reaction	Transient flushing or rash; drug fever <38°C(<100.4°F)	Rash; flushing; urticaria; dyspnea; drug fever≥38°C(≥100.4°F)	Symptomatic bronchospasm, with or without urticaria; parenteral medication(s) indicated; allergy-related edema/angioedema; hypotension	Anaphylaxis	Death
**Skin**					
Rash	Macular or papular eruption or erythema without associated symptoms	Macular or papular eruption or erythema with pruritus or other associated symptoms;	Severe, generalized erythroderma or macular, papular or vesicular eruption;	Generalized exfoliative, ulcerative, or bullous dermatitis	Death

Adverse Event	Grade				
	I	II	III	IV	V
		localized desquamation or other lesions covering <50% of body surface area (BSA)	desquamation covering ≥50% BSA		
Alopecia	Baldness	Thinning or patchy	Complete	—	—
CONSTITUTIONAL SYMPTOMS					
Fatigue	Mild fatigue over baseline	Moderate or causing difficulty performing some ADL	Severe fatigue interfering with ADL	Disabling	—
**Infection**					
Febrile neutropenia (fever of unknown origin without clinically or microbiologically documented infection) (ANC <1.0 x 109/L, fever ≥38.5°C)	—	—	Present	Life-threatening consequences (e.g., septic shock, hypotension, acidosis, necrosis)	Death
**Cardiovascular**					
Prolonged QTc	QTc >0.45 - 0.47 second	QTc >0.47 - 0.50 second; ≥0.06 second above baseline	QTc >0.50 second	QTc >0.50 second; life threatening signs or symptoms	Death

Adverse Event	Grade				
	I	II	III	IV	V
				(e.g., arrhythmia, CHF, hypotension, shock syncope); Torsade de pointes	
Cardiac ischemia/infarction	Asymptomatic arterial narrowing without ischemia	Asymptomatic and testing suggesting ischemia; stable angina	Symptomatic and testing consistent with ischemia; unstable angina; intervention indicated	Acute myocardial infarction	Death
Left ventricular systolic dysfunction	Asymptomatic, resting ejection fraction (EF) <60 - 50%; shortening fraction (SF) <30 - 24%	Asymptomatic, resting EF <50 - 40%; SF <24 - 15%	Symptomatic CHF responsive to intervention; EF <40 - 20% SF <15%	Refractory CHF or poorly controlled; EF <20%; intervention such as ventricular assist device, ventricular reduction surgery, or heart transplant indicated	Death
Cardiac troponin I (cTnI)	—	—	Levels consistent with unstable angina as defined by the manufacturer	Levels consistent with myocardial infarction as defined by the manufacture	Death

Adverse Event	Grade				
	I	II	III	IV	V
				r	
Cardiac troponin T (cTnT)	0.03 - <0.05 ng/mL	0.05 - <0.1 ng/mL	0.1 - <0.2 ng/mL	0.2 ng/mL	Death
**edema**					
Edema: head and neck	Localized to dependent areas, no disability or functional impairment	Localized facial or neck edema with functional impairment	Generalized facial or neck edema with functional impairment (e.g., difficulty in turning neck or opening mouth compared to baseline)	Severe with ulceration or cerebral edema; tracheotomy or feeding tube indicated	Death
Edema: limb	5 - 10% inter-limb discrepancy in volume or circumference at point of greatest visible difference; swelling or obscuration of anatomic architecture on close inspection; pitting edema	>10 - 30% inter-limb discrepancy in volume or circumference at point of greatest visible difference; readily apparent obscuration of anatomic architecture; obliteration of skin folds; readily apparent deviation from normal anatomic contour	>30% inter-limb discrepancy in volume; lymphorrhea; gross deviation from normal anatomic contour; interfering with ADL	Progression to malignancy (i.e., lymphangio sarcoma); amputation indicated; disabling	Death

Adverse Event	Grade				
	I	II	III	IV	V
Edema: trunk/genital	Swelling or obscuration of anatomic architecture on close inspection; pitting edema	Readily apparent obscuration of anatomic architecture; obliteration of skin folds; readily apparent deviation from normal anatomic contour	Lymphorrhea; interfering with ADL; gross deviation from normal anatomic contour	Progression to malignancy (i.e., lymphangio sarcoma); disabling	Death
Edema: viscera	Asymptomatic; clinical or radiographic findings only	Symptomatic; medical intervention indicated	Symptomatic and unable to aliment adequately orally; interventional radiology or operative intervention indicated	Life-threatening consequences	Death
**Nervous system**					
Dizziness	With head movements or nystagmus only; not interfering with function	Interfering with function, but not interfering with ADL	Interfering with ADL	Disabling	—
Insomnia	Occasional difficulty sleeping, not interfering with function	Difficulty sleeping, interfering with function but not interfering with ADL	Frequent difficulty sleeping, interfering with ADL	Disabling	—

**Adverse Event**	**Grade**				
	**I**	**II**	**III**	**IV**	**V**
Pain (non-tumor)	Mild pain not interfering with function	Moderate pain; pain or analgesics interfering with function, but not interfering with ADL	Severe pain; pain or analgesics severely interfering with ADL	Disabling	—

Annex 4 Classfication Criteria of Neurotoxicity

**Adverse Event**	**Grade**				
	**1**	**2**	**3**	**4**	**5**
Peripheral sensory neuropathy	Asymptomatic; clinical or diagnostic observations only; intervention not indicated	Moderate symptoms; limiting instrumental ADL *	Severe symptoms; limiting self care ADL **; assistive device indicated	Life-threatening consequences; urgent intervention indicated	Death
Definition: A disorder characterized by inflammation or degeneration of the peripheral sensory nerves.					

Activities of Daily Living (ADL)

* Instrumental ADL refer to preparing meals, shopping for groceries or clothes, using the telephone, managing money, etc.

** Self care ADL refer to bathing, dressing and undressing, feeding self, using the toilet, taking medications, and not bedridden.

Annex 5 Relationship Between Adverse Events and Experimental Drugs

According to the National Adverse Drug Reaction Reporting and Monitoring Management Approach, the relevance evaluation is classified into 6 types on the basis of the 5 criteria about adverse reactions/events: certain, probable, possible, probable not, pending evaluation and unable to evaluate.

**Classification**	**1**	**2**	**3**	**4**	**5**
Certain	+	+	+	+	-
Probable	+	+	+	?	-
Possible	+	±	±?	?	±?
Probable not	-	-	±?	?	±?
Pending evaluation	Additional materials are required for evaluation				
Unable to evaluate	The information requested for evaluation is not available				

Note: "+" means "yes"; "-" means "no"; "±" means "no final conclusion"; "?" means "unknown".

Five criteria for the analysis of adverse reactions/events:

1. Is there a reasonable time relationship between the medication and the occurrence of adverse reaction/event?

2. Does the response/event conform to the known type of adverse reactions of the drug?

3. Does the response/event disappear or alleviate when stopping taking the drug or reducing the dosage of the drug?

4. Will the same reaction/event appear again when the suspected drug is administrated again?

5. Can the response/event be explained by the effect of co-administration, the progression of the patient's condition or the impact of other treatments?

If relevance evaluation is deemed to be certain, probable or possible, then the response/event can be regarded as an adverse reaction caused by the drug, but whether it is a serious adverse event should be judged according to the severity.

Annex 6 Important Information for Participants and Informed Consent Documentation

Scheme title: A multicenter, randomized, double-blinded, controlled phase II clinical trial of thalidomide in the treatment of transfusion-dependent β-thalassemia major/intermediate

Solemn statement: if you do not fully understand the content of this informed consent, please consult researchers or relevant staff and do not sign this form without satisfactory answers.

Dear patients (family members):

Please read the following content as carefully as possible before you decide whether to participate in this study. The following content can help you learn about the study, including its purpose, the procedures and duration, and the benefits, risks and discomforts derived from participation. You can also consult with your relatives and friends, or ask a doctor to provide reasonable explanations, to help you make a decision regarding participation, as you wish.

1 Research background

Thalassemia, a hereditary and hemolytic anemia, is caused by a deficiency in one or more globin chains in Hb on account of genetic defects. According to the severity, the disease can be divided into thalassemia major, intermediate, and minor. The disease is widely distributed in many parts of the world; however, it is more common in Guangdong, Guangxi, and Sichuan. Thalassemia minor does not require special treatment, but thalassemia intermediate and major should be treated with blood transfusions, iron chelators, splenectomy, or hematopoietic stem cell transplantation. However, long-term blood transfusions and iron removal treatment are all expensive. Splenectomy has a good effect on Hb H disease, but poor effects on β-thalassemia intermediate and major; furthermore, splenectomy can increases the risk of weakened immunity, thrombogenesis, etc., and is a traumatic operation. Hematopoietic stem cell transplantation (HSCT) is an effective method to radically cure β-thalassemia major and it should be the first choice in treating β-thalassemia major if there are human leukocyte antigen-matched donors of hematopoietic stem cells. Additionally, the success rate of allogeneic HSCT is lower in patients with thalassemia who have older age, more complications, and poor organ function.

2 Research purpose

This study is a multicenter, randomized, double-blinded, placebo-controlled II clinical trial for transfusion-dependent β-thalassemia (Transfusion dependence, defined as receiving at least

eight transfusions or at least 100 ml/kg of their body weight in packed red cells per year; or frequent transfusion was required to maintain Hb > 70 g/L in the 2 years before enrollment). The main objective of the study is to evaluate whether thalidomide has a better clinical outcome compared to that with placebo in the treatment of transfusion-dependent β-thalassemia.

1) At present, thalassemia still lacks a standardized, effective, and economical treatment. Thus, the purpose of this study is to explore a new medical treatment for thalassemia. The study of thalidomide treatment in patients with thalassemia is expected to verify that it can effectively improve the Hb level, reduce or negate the need of blood product infusions, and continuously improve the quality of life of patients.

2) The use of thalidomide in patients with thalassemia is expected to significantly reduce the cost of medical treatment, and effectively reduce the economic burden of patients.

3 Possible benefits of participating in the study

Thalassemia currently lacks effective drug therapy, and usually relies on blood transfusions to improve the Hb level and quality of life. However, blood transfusions can lead to iron overload, with excessive iron deposition in the heart, liver, pancreas and other organs, resulting in organ dysfunction, and even organ failure.

In this study, thalidomide treatment in patients with thalassemia is expected to improve the Hb level, reduce or negate the need of blood product infusions, and continuously improve the quality of life of patients.

You and society may benefit from this study, which includes the possibility of improvement in your condition. Additionally, this study may help confirm an effective treatment for other patients with similar conditions. No matter which group you are assigned to, you will receive at least basic blood transfusions, as the current treatment of thalassemia.

The sponsors will provide the study medications (thalidomide/placebo) free of charge and bear the cost of the examinations related to this study, including the cost of blood transfusions during the study period (the researcher will bear the remaining research-related expenses, after reimbursement by the local medical insurance; any remaining costs will be paid by the patient). Note: The blood transfusion standards in this study are as follows: Hb < 70 g/L, or Hb > 70 with cardiac function reaching New York Heart Association (NYHA) heart disease grading standard III grade or above.

The sponsors will provide a certain amount of transportation subsidy, as well as a blood nutrition subsidy, as needed for scientific research.

4 Introduction of research drugs

4.1 Thalidomide

According to “Chinese Pharmacopoeia 2010 Edition”, thalidomide is classified into the following categories:

1) Antipyretic, analgesic, anti-inflammatory, and anti-rheumatic drugs and anti-gout drugs (P1013);

2) Medication in dermatology (P1366);

3) Medication in stomatology (P1450).

At present, thalidomide drug administration is only indicated for the control of leprosy disease; it is not currently indicated for the treatment of thalassemia and other diseases of the blood system. However, in recent years, many beneficial explorations have been carried out regarding thalidomide in the treatment of blood system diseases at home and abroad, and great progress has been made. A literature search conducted by Guangxi Medical Science Information Research Institute (as of 2017-06-28) indicated that the clinical application results of thalidomide in the treatment of thalassemia have been reported abroad (as case reports), and research results of thalidomide in the treatment of chronic anemia have been reported in China.

At the same time, the Chinese expert consensus on the diagnosis and treatment of myelodysplastic syndrome (MDS) (2014 edition) indicated that thalidomide treatment can improve erythroid hematopoiesis and reduce or negate transfusion dependence for some patients with MDS. Chinese guidelines for the diagnosis and treatment of multiple myelomas (2015 edition) include thalidomide as a multiple first-line treatment option for first-care patients. Thalidomide is included in the Chinese expert consensus on the diagnosis and treatment of primary bone marrow fibrosis (PMF) (2015 edition) with PMF anemia treatment regimens.

In summary, although thalassemia is not currently included in the indications of thalidomide drug administration, the efficacy and safety of thalidomide in other diseases of the blood system have been recognized, and thalidomide has been included in several recommended treatment protocols in blood disease guidelines.

4.2 Placebo

In this study, the placebo, a white or whitish tablet, is a formulation that does not contain thalidomide components, with exactly the same traits as those in the thalidomide preparation. Like thalidomide, the placebo is orally administered at a dose of 100-150 mg/d per night, after dinner.

5 Research process

5.1 Informed consent

If you agree to participate in this study, you must sign your name and provide the date on the signature page. Under special circumstances, you may entrust your legal guardian to sign on your behalf. You will then enter the screening period after signing the informed consent.

5.2 Screening

Researchers will judge whether you are suitable as a participant in this research by screening. You will undergo the following inspections and evaluations:

1) Medical history inquiry, which collects data regarding your medical history, previous genetic diagnoses, pre-screening treatments and other complications, history of medication, and the presence of fever and blood transfusions in the last 3 months;

2) Inquiries regarding the accurate number and amount of blood transfusions (red cell units) in the last 12 months;

3) Thalidomide guidance;

4) Physical examinations, including the assessment height, weight, blood pressure, heart rate;

5) Routine blood examinations, including the determination of the blood white blood cell classification count, Hb level, red blood cell count, platelet count, reticulocyte count, and nuclear red blood cell count;

6) Hb analysis for the levels of Hb A, Hb A2, and Hb F;

7) Detection of thalassemia;

8) Blood biochemical examinations for the levels of aspartate transaminase (AST), alanine transaminase (ALT), total bilirubin (TBIL), (direct bilirubin) DBIL, (indirect bilirubin) IBIL, blood urea nitrogen (BUN), creatine (Cr), and lactate dehydrogenase (LDH);

9) Anti-human globulin test and tri-solution test (sugar, heat, acid);

10) Assessment of three items of anemia (folic acid, vitamin B12, and serum ferritin) and EPO determination;

11) Red blood cell lifespan assessment;

12) Routine urine examination, including the levels of white blood cells, urine protein, and red blood cells;

13) Standard 12-lead electrocardiography;

14) Echocardiography, upper abdominal magnetic resonance;

15) Pregnancy test: for female participants of childbearing age;

16) Disease status assessment;

17) Patient self-assessment via a questionnaire commonly used internationally. You will be required to answer 50 simple questions so that your doctor can fully understand your health condition during the trial;

Your research physician will synthetically determine whether you can participate in this study according to the above examination results and your situation. If you are not suitable for the study after screening, the research physician will provide you with other appropriate treatments after consulting with you.

5.3 Treatment

Thalidomide will be added to traditional blood transfusion therapy, continuously administered at a dose of 100-150 mg/d. During the 3 months of treatment, routine blood, liver, and kidney function tests will be performed, and Hb electrophoresis will be monitored regularly.

5.4 Follow-up

In order to ensure the safety of your medication, you need to carry out the inspections and visits required during the follow-up period on the prescribed dates. Please follow the arrangements made by the research physician.

5.5 Research period

If you agree to participate in the study, the period from the date of signing the consent form to the end of the study treatment and the end of the observation period is the so-called “time of participation in the study”. During the study, the research physician will fully observe the treatment efficacy and adverse reactions. The efficacy is evaluated after 12 weeks of treatment (i.e. the end of placebo-controlled period). For patients with a lack of efficacy, the trial data of the first 12 weeks are sealed and the patient can choose to enter into the extension phase at their own request. For patients with a good, or even excellent, response, the trial data of the first 12 weeks

are sealed, and the original treatment is continuously administered for further evaluation in the extension phase.

6 Things you should pay attention to in your research

1) You may get varying degrees of improvement during this trial, including disease stability (i.e., no disease progression), disease remission, etc. No matter what type of improvement you experience, we recommend that you continue to receive treatment and do not quit at will, because patients presenting with disease stability still have a chance to get better relief as treatment continues.

2) You must carefully complete the questionnaire during the screening period and at each course of treatment, which will help the doctor fully understand your health condition;

3) You should come to the hospital according to the follow-up schedule made with the research doctor. The doctor will judge the safety and effectiveness of your treatment; thus, every visit is important;

4) You should follow the guidance of the research doctor and objectively complete the "medication diary card" in a timely manner after each medication;

5) You are required to return unused drugs and packaging during each visit, and bring in other medications you are taking simultaneously, including those you have to continue taking for other illnesses, so that the doctor make detailed records;

6) If you are using drugs other than those prescribed by your doctor, be sure to tell the research doctor;

7) If you have received treatment at another hospital or department, please tell the research doctor;

8) Please follow the doctor's advice as much as possible and come to the hospital for examinations on time;

9) If unexpected adverse events occur during the study, please notify the doctor immediately;

10) Thalidomide can cause birth defects (deformed fetus), including extremity disabilities, deformity of the legs and arms, and fetal death. If you are pregnant or preparing to become pregnant, or your spouse is preparing to become pregnant, you are not allowed to participate in this study. Fertile female participants or male participants with fertile spouses must use reliable contraceptive methods or suppress sensual passion completely from 28 days prior to treatment to

28 days after the end of treatment. Researchers will provide you with detailed thalidomide risk education and guidance. If you or your spouse becomes pregnant during this period, you will bear all the consequences. Please notify the research doctor immediately if you discover an unwanted pregnancy;

11) There are no special requirements for your daily diet and daily life.

7 Possible risks of participating in the study

7.1 Thalidomide side effects

1) Thalidomide is reported to have a strong teratogenic effect. Taking it in early pregnancy can cause fetal malformation, known as a short-legged seal fetus;

2) Thalidomide can cause peripheral neuropathy, manifested as numbness of the hands and feet, muscle tightness, and a feeling of weakness in the lower extremities, and can also cause multiple neuritis;

3) Thalidomide can also cause gastrointestinal discomfort, dry mouth, bitter mouth, loss of appetite, constipation, dizziness, headaches, lethargy, etc., and even neutropenia may occur. In severe cases, patients need to stop the medicine and be given symptomatic treatment. Occasionally, allergies cause drug eruptions. There are rare reports in the literature that taking this product may also cause thromboembolisms;

4) An adverse drug reaction in children has not been clarified; however, the drug instructions clearly indicate that children are prohibited from taking thalidomide;

5) Other adverse reactions that have not been discovered as yet are also possible.

7.2 Response

1) For women of childbearing age, effective contraceptive measures should be started at least 4 weeks before taking the medicine. The pregnancy test should be done in the first month; if the test is negative, the participant can continue taking the medicine, but should retake a pregnancy test when necessary. The patient may become pregnant at least 4 weeks after drug cessation. Breastfeeding is not allowed during medication and within 4 weeks of drug withdrawal;

2) Male participants should inform their sexual partners of the possible risks and must use a condom before any sexual contact with a fertile female during medication and within 4 weeks after drug cessation. Blood donation and sperm donation are forbidden during this period;

3) If a female participant or the sexual partner of a male participant becomes pregnant during

treatment, the woman must immediately stop using thalidomide and consult a doctor to deal with the fetus accordingly;

4) For patients with a high incidence of cardiovascular disease, attention must be paid to symptoms of heart failure and thrombosis, which can be prevented by taking aspirin. If patients are taking β-receptor antagonists during treatment, even more careful attention should be paid. Stop taking the study medicine and undergo symptomatic treatment when necessary;

5) The blood will be checked periodically during medication, and patients with an absolute neutrophil count below 750/mm^3 should stop taking the study drug.

7.3 Those who are not suitable for participation in the study

1) Those who have received EPO, Yisui Shengxue granules, hydroxyurea, thalidomide, decitabine, azacitidine, or butyrate within 3 months before enrollment;

2) Patients with comorbid severe cardiopulmonary or renal insufficiency;

3) Patients with comorbid cardiovascular and cerebrovascular diseases, malignant tumors, or other severe diseases;

4) Patients with a history of intravenous or arterial thrombosis within 3 months;

5) Patients with a merger of anemia caused by other reasons;

6) Patients with mental disease;

7) Patients who cannot take oral drugs;

8) Patients who are pregnant or lactating, or who are at childbearing age and unwilling to take contraceptive measures;

9) Patients who are allergic to this product and need to be engaged in driving and machine operation during the study;

10) Patients who are currently participating in other clinical trials;

11) Patients with other conditions considered not suitable for participation by the researchers.

During the study period, patients need to come to the hospital regularly as required. If there is any discomfort, a new change in your condition, or an unexpected situation, contact the hospital team in a timely manner to allow a timely judgment and early appropriate medical treatment, regardless of whether it is related to the study. At the same time, the research group will assess whether you should continue to use thalidomide for treatment.

8 Related costs

1) The sponsors will provide the study medications (thalidomide/placebo) free of charge and bear the cost of the examinations related to this study, including the cost of blood transfusions during the study period (the researcher will bear the remaining research-related expenses, after reimbursement by the local medical insurance; any remaining costs will be paid by the patient). Remarks: The blood transfusion standards in this study are as follows: Hb level < 70 g/L, or Hb level > 70 g/L with cardiac function reaching NYHA heart disease classification level III or above;

2) If the patient has an adverse reaction, the cost of treatment or prevention will not be free;

3) The treatment and examination of other comorbid diseases will not be free;

4) The sponsor is responsible for expenses related to regular follow up;

5) Patients are required to provide specimens for testing due to the needs of the study research, but the costs of testing are borne by the hospital.

9 Confidentiality of research records

Participants' participation and their personal data in the trial are all confidential content, and the records identifying the participants will be kept confidential. If the study results are published publicly, the identity of the participants will also be kept confidential.

10 Participants' rights

Participants participate in the trial voluntarily, and can refuse to participate at any stage of the trial, or withdraw from the trial at any time. Participants will not be submitted to discrimination or retaliation, and their medical treatment and rights will not be affected. However, participants are expected to complete the study as much as possible without special exceptions. Participants must tell the doctor in charge if he/she is withdrawing, in any case.

The participant's medical records (medical records, laboratory tests, etc.) will be kept intact in the hospital you visit, and the doctor will record the test results in the medical records. Researchers, ethics committees, and drug regulatory authorities are allowed to review the participants' medical records. Any public report on the results of this research will not disclose personal identities. We will make every effort to protect the privacy of your personal medical information, to the extent permitted by law.

You can voluntarily choose to participate in the study and can withdraw from the study, which is determined entirely on your wishes. You can refuse to participate in the study or

withdraw from the study at any time during the trial. This will not affect the relationship between you and the doctor, nor will it cause a loss to your medical or other interests.

For the sake of your best interests, the doctor or researcher may suspend your participation in the study at any time.

If you withdraw from the study for any reason, you may be asked about your use of the test drug. If the doctor thinks it is necessary, you may also be required to undergo laboratory and physical examinations.

11 Consulting contact information

If you have any questions about the study or think that adverse reactions have resulted from participating in the study, you can contact the research physician of the clinical trial at any time: _____; telephone number:_____.

This informed letter is completed in duplicate, one copy is for the participant and one copy is for the researcher, the letter is valid after both parties sign it.

The patient (family member) endorses the following statements:

I have understood the above situation and agree to participate in this study. I will cooperate with the clinician in strict accordance with the research requirements. Additionally, I will follow up in a timely manner and report any discomfort in a timely manner.

I have learned about the risks of thalidomide to pregnancy. In order to avoid pregnancy during medication, I will choose strict contraception measures and use condoms throughout the course of any sexual contact. If there is a need for pregnancy, I will inform the doctor more than 4 weeks in advance and stop taking the medicine in time.

The current long-term application of thalidomide does not rule out the possibility of unknown adverse events, but long-term anemia and organ iron deposition in patients with thalassemia can itself cause abnormalities or damage to organ functions and physical development. I understand this and agree to use thalidomide.

Patient’s signature: Date:

Physician’s signature: Date:

Annex 7 Questionnaire on Participants' Health Status

Mapi

LINGUISTIC VALIDATION CERTIFICATE

TranQoL

This is to certify that **Mapi Language Services** conducted the linguistic validation of the paper version of the **TranQoL** (source file name: **TranQol-Adult_AU1.0_eng-CAori**) into **Mandarin Chinese for Taiwan**.

The aim of linguistic validation is to obtain translations that are:

- conceptually equivalent to the original and comparable across languages;
- culturally relevant to the context of the target country;
- easily understood by the people to whom the translated instrument is administered.

This is achieved using a rigorous methodology[1] involving:

- a process which comprises several steps;
- the instrument's developer input on conceptual issues;
- a skilled team recruited by **Mapi Language Services** in the target country and headed by a consultant with knowledge of and experience in the field of Clinical Outcome Assessments. The consultant supervises and coordinates the linguistic validation process in his/her country;
- a centralized review process coordinated by **Mapi Language Services**, including quality control by linguists and discussions about translation decisions with the consultant at each step of the process.
- cross-cultural harmonisation to ensure common understanding of the instrument's concepts by all participants involved in the process and achieve conceptual equivalence across languages.

The aforementioned translation (filename: TranQol_Adult_AU1.0_cmn-TW, dated 11 January 2016) underwent the following steps:

- Forward Translation step – 2 forward translations by qualified translators and reconciliation
- Backtranslation step – 1 backtranslation by a qualified translator
- Developer's Review step
- Proofreading step

Mapi Language Services may not be held liable for any changes made on the translation after completion of projet **052273** by **Mapi Language Services** on **11 January 2016**.

Approved By:

Ana BAYLES
Managing Director
Mapi - Language Services

Mapi SAS
SAS au capital de 434 700 euros
27 rue de la Villette
69003 LYON
Tél. 04 72 13 66 67 - Fax 04 72 13 69 50
RCS Lyon 378 472 872 - TVA n° FR 66 378 472 872

[1] **References:**

- Acquadro C., Jambon B., Ellis D. and Marquis P. Language and translation issues. In Spilker B, ed. Quality of Life and Pharmacoeconomics in Clinical Trials. Philadelphia: Lippincott-Raven Publishers, 1996: 575-585.
- Linguistic Validation Manual for Health Outcomes Assessments. Acquadro C, Conway K, Giroudet C, Mear I. Second Edition - Mapi Institute, Lyon, France, January 2012 - ISBN: 2-9522021-0-9

TranQoL - Mandarin/Taiwan - Version of 11 January 16 - Mapi.
ID052273 / TranQol_Adult_AU1.0_cmn-TW
© Copyright 2015. Mapi. All Rights Reserved. Mapi LS T PM 310 v2.0 updated 21 12 2015

編號：________________

日期：________________

TranQol

adult

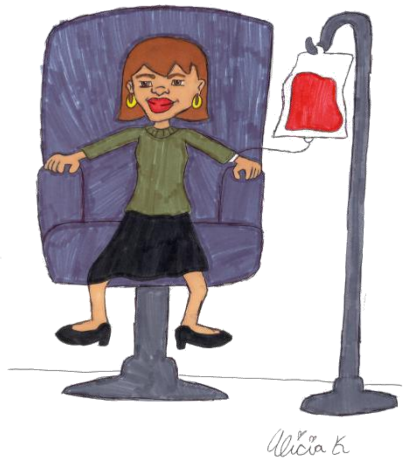

說明

針對此問卷，我們要求您回答有關**自己的狀況。** 本研究想要獲得您過去 7 天的經驗， 因此我們要求您**僅專注於過去 7 天。** 以勾選 (✓) 的方式在最符合您狀況的方格中記錄答案。 回答時請考慮您的一般狀況；不用擔心某答案是否由地中海型貧血所引起。

記住：選擇最符合您狀況的答案：（每一題只選擇**一個答案**)

答案沒有對錯之分， 重要的是您的看法。

舉例:

在過去 7 天…	從未	幾乎沒有	有時	經常
1. 我覺得無聊	❑	❑	✓	❑

註：您可以在問卷的最後提供其他意見

針對每一個項目，請告訴我們過去 **7** 天這些事情在您身上發生的頻率…

我們想要瞭解**您的身體健康**	從未	幾乎沒有	有時	經常	總是
1. 我有睡眠問題…	❑	❑	❑	❑	❑
2. 我沒有疼痛或不舒服…	❑	❑	❑	❑	❑
3. 疼痛讓我無法做我必須做的事…	❑	❑	❑	❑	❑
4. 我有足夠活力從事每日活動…	❑	❑	❑	❑	❑
5. 我無法從事需要中度體力要求的活動…	❑	❑	❑	❑	❑
6. 我無法從事需要高度體力要求的活動…	❑	❑	❑	❑	❑
7. 我覺得過於疲憊，無法從事我喜愛的活動… (即興趣、工藝、運動、樂器等)	❑	❑	❑	❑	❑
8. 我發現自己難以和同年紀的人做一樣事情…	❑	❑	❑	❑	❑
9. 我的健康狀況允許我可以盡可能參與我想要的社交活動…	❑	❑	❑	❑	❑
10. 我的治療讓我無法做我想做的事…	❑	❑	❑	❑	❑
我們想要瞭解**您的情緒健康**	**從未**	**幾乎沒有**	**有時**	**經常**	**總是**
11. 我感到悲傷…	❑	❑	❑	❑	❑
12. 我擔心我在接受輸血時受到感染…	❑	❑	❑	❑	❑
13. 我擔心健康越來越惡化…	❑	❑	❑	❑	❑
14. 我感到憤怒…	❑	❑	❑	❑	❑

請告訴我們過去 **7** 天這些事情在您身上發生的頻率…

我們想要瞭解**您的情緒健康（續上頁）…**	從未	幾乎沒有	有時	經常	總是
15. 我感到焦慮…	❑	❑	❑	❑	❑
16. 我覺得我的未來有希望…	❑	❑	❑	❑	❑
17. 我能自在地和親近的人談論我的狀況…	❑	❑	❑	❑	❑
18. 我擔心地中海型貧血可能會讓我提早死亡…	❑	❑	❑	❑	❑
19. 我擔心會痛的檢查…	❑	❑	❑	❑	❑
20. 我擔心是否能夠有小孩…	❑	❑	❑	❑	❑
21. 我對於輸血感到困擾…	❑	❑	❑	❑	❑
22. 我對於排鐵治療感到困擾…	❑	❑	❑	❑	❑
23. 我對於錯過治療感到有罪惡感…	❑	❑	❑	❑	❑
24. 我覺得自己跟別人不一樣…	❑	❑	❑	❑	❑
不管您目前性生活的狀況，請回答以下問題。 若您不想回答，請勾選此❑方格，並進到下一個單元。					
	從未	幾乎沒有	有時	經常	總是
25. 我對我的性生活感到滿意…	❑	❑	❑	❑	❑
我們想要瞭解**您的家庭…**	從未	幾乎沒有	有時	經常	總是
26. 家人給我足夠的支持…	❑	❑	❑	❑	❑
27. 朋友給我足夠的支持…	❑	❑	❑	❑	❑
28. 地中海型貧血對我家庭產生負面影響…	❑	❑	❑	❑	❑

請告訴我們過去 **7** 天這些事情在您身上發生的頻率…

我們想要瞭解**您的家庭（續上頁）…**	從未	幾乎沒有	有時	經常	總是
29. 我擔心藥物/醫材的花費…	❑	❑	❑	❑	❑
30. 我的治療對我/我的家庭造成財務壓力… （即往返醫院的交通、醫材、無法上班的時間）	❑	❑	❑	❑	❑
我們想要瞭解**您的學習與工作…**	從未	幾乎沒有	有時	經常	總是
31. 我難以跟上學校課業…<br>**或** 我沒有上學 ☐	❑	❑	❑	❑	❑
32. 地中海型貧血以及它的治療影響我的課業表現…<br>**或** 我沒有上學 ☐	❑	❑	❑	❑	❑
33. 我因為缺課而感到困擾…<br>**或** 我沒有上學 ☐	❑	❑	❑	❑	❑
34. 我難以做到原本該做的工作…<br>**或** 我沒有工作 ☐	❑	❑	❑	❑	❑
35. 我因為錯過工作而感到困擾…<br>**或** 我沒有工作 ☐	❑	❑	❑	❑	❑
36. 地中海型貧血限制了我的工作機會…	❑	❑	❑	❑	❑

還有其他任何事情困擾您嗎？

____

____

____

____

Annex 8 Health Questionnaire

Number of sub-center ________ Number of participants________

Initials ________ Number of courses________

If the participant himself fills in the form: ☐ Yes ☐ No

Date of filling ________

EORTC QLQ-C30 (V3.0)

We would like to know some information about you and your health and please answer all the questions below personally. The questions here have no “right” or “wrong” answers, so just draw a circle on the number that most closely matches your situation. The information you provide will be kept strictly confidential.

	no	a bit	very	quite
1. Do you have difficulty in some strenuous activities, for example, carrying a heavy shopping bag or suitcase?	1	2	3	4
2. Is it difficult for you to walk long distances?	1	2	3	4
3. Is it difficult for you to walk short distances outdoors?	1	2	3	4
4. Do you need to stay in bed or chair during the day?	1	2	3	4
5. Do you need any help from others when eating, dressing, bathing or going to the toilet?	1	2	3	4

Over the past week:	no	a bit	very	quite
6. Do you feel restricted for your work and daily activities	1	2	3	4
7. Do you feel restricted when engaging in your hobbies or leisure activities?	1	2	3	4
8. Are you short of breath?	1	2	3	4
9. Do you have any pain?	1	2	3	4
10. Do you need some rest?	1	2	3	4
11. Do you have trouble sleeping?	1	2	3	4
12. Do you feel weak?	1	2	3	4
13. Do you suffer from loss of appetite (no appetite)?	1	2	3	4
14. Do you feel nausea?	1	2	3	4

15. Have you vomited?	1	2	3	4
16. Do you suffer from constipation?	1	2	3	4

Over the past week:	no	a bit	very	quite
17. Do you suffer fromdiarrhea?	1	2	3	4
18. Do you feel tired?	1	2	3	4
19. Does pain affect your daily activities?	1	2	3	4
20. Do you have difficulty in concentrating on things, like reading newspapers or watching TV?	1	2	3	4
21. Do you feel nervous?	1	2	3	4
22. Do you feel worried?	1	2	3	4
23. Do you feel short-tempered?	1	2	3	4
24. Do you feel depressed?	1	2	3	4
25. Do you have difficulty in remembering things?	1	2	3	4
26. Does your physical condition or treatment affect your family life?	1	2	3	4
27. Does your physical condition or treatment affect your social activities?	1	2	3	4
28. Does your physical condition or treatment put you into financial difficulty?	1	2	3	4

Please choose a suitable number from 1-7 that most closely matches you and draw a circle for the following questions.

29. How do you evaluate your overall health condition over the past week?

1 2 3 4 5 6 7

quite poor quite good

30. How do you evaluate your total quality of life over the past week?

1 2 3 4 5 6 7

quite poor quite good

Patients sometimes point the following symptoms or problems. Please indicate the degree of these symptoms or problems over the past week and circle the number that most closely matches your situation.

Over the past week:	no	a bit	very	quite
31. Have you suffered from bone pain?	1	2	3	4
32. Have you suffered from back pain?	1	2	3	4
33. Have you suffered from pain in your knee (hipbone)?	1	2	3	4
34. Have you suffered from pain in your arm or shoulder?	1	2	3	4
35. Have you suffered from chest pain?	1	2	3	4
36. If you are suffering from pain, does the intensity increase with activity?	1	2	3	4
37. Have you ever felt sleepy?	1	2	3	4
38. Have you ever felt thirsty?	1	2	3	4
39. Do you feel sick?	1	2	3	4
40. Have you ever felt dry in the mouth?	1	2	3	4
41. Do you have hair loss?	1	2	3	4
42. If you have hair loss, please answer this question: do you feel upset about hair loss?	1	2	3	4
44. Have you suffered from prickling in your hands or feet?	1	2	3	4
44. Have you ever felt restless or emotional?	1	2	3	4
45. Have you suffered from hyperacidity or stomach heat?	1	2	3	4
46. Have you suffered from burning pain or sore in your eyes?	1	2	3	4

Over the past week:	no	a bit	very	quite
47. Do you feel that your physical attractiveness has diminished due to your disease or treatment?	1	2	3	4
48. Do you always think about your disease?	1	2	3	4
49. Have you ever worried about death?	1	2	3	4
50. Have you ever worried about your future health?	1	2	3	4

Annex 9 World Medical Association Declaration of Helsinki

Ethical Principles for Medical Research Involving Human Subject

Adopted by the 18th WMA General Assembly, Helsinki, Finland, June 1964, and amended by:

29th WMA General Assembly, Tokyo Japan, October 1975;

35th WMA General Assembly, Venice, Italy, October 1983;

41st WMA General Assembly, Hong Kong September 1989;

48th WMA General Assembly, Somerset West, Republic of South Africa, October 1996;

52nd WMA General Assembly, Edinburgh, Scotland, October 2000.

A. Introduction

1. The World Medical Association has developed the Declaration of Helsinki as a statement of ethical principles to provide guidance to physicians and other subjects in medical research involving human subjects. Medical research involving human subjects includes research on identifiable human material or identifiable data.

2. It is the duty of the physician to promote and safeguard the health of the people. The physician's knowledge and conscience are dedicated to the fulfillment of this duty.

3. The Declaration of Geneva of the World Medical Association binds the physician with the words, ‘‘The health of my patient will be my first consideration,’’ and the International Code of Medical Ethics declares that, ‘‘A physician shall act only in the patient's interest when providing medical care which might have the effect of weakening the physical and mental condition of the patient.’’

4. Medical progress is based on research which ultimately must rest in part on experimentation involving human subjects.

5. In medical research on human subjects, considerations related to the well-being of the human subject should take precedence over the interests of science and society.

6. The primary purpose of medical research involving human subjects is to improve prophylactic, diagnostic and therapeutic procedures and the understanding of the aetiology and pathogenesis of disease. Even the best proven prophylactic, diagnostic, and therapeutic methods must continuously be challenged through research for their effectiveness, efficiency, accessibility and quality.

7. In current medical practice and in medical research, most prophylactic, diagnostic and

therapeutic procedures involve risks and burdens.

8. Medical research is subject to ethical standards that promote respect for all human beings and protect their health and rights. Some research populations are vulnerable and need special protection. The particular needs of the economically and medically disadvantaged must be recognized. Special attention is also required for those who cannot give or refuse consent for themselves, for those who may be subject to giving consent under duress, for those who will not benefit personally from the research and for those for whom the research is combined with care.

9. Research Investigators should be aware of the ethical, legal and regulatory requirements for research on human subjects in their own countries as well as applicable international requirements. No national ethical, legal or regulatory requirement should be allowed to reduce or eliminate any of the protections for human subjects set forth in this Declaration.

B. Basic principles for all medical research

10. It is the duty of the physician in medical research to protect the life, health, privacy, and dignity of the human subject.

11. Medical research involving human subjects must conform to generally accepted scientific principles, be based on a thorough knowledge of the scientific literature, other relevant sources of information, and on adequate laboratory and, where appropriate, animal experimentation.

12. Appropriate caution must be exercised in the conduct of research which may affect the environment, and the welfare of animals used for research must be respected.

13. The design and performance of each experimental procedure involving human subjects should be clearly formulated in an experimental protocol. This protocol should be submitted for consideration, comment, guidance, and where appropriate, approval to a specially appointed ethical review committee, which must be independent of the investigator, the sponsor or any other kind of undue influence. This independent committee should be in conformity with the laws and regulations of the country in which the research experiment is performed. The committee has the right to monitor ongoing trials. The researcher has the obligation to provide monitoring information to the committee, especially any serious adverse events. The researcher should also submit to the committee, for review, information regarding funding, sponsors, institutional affiliations, other potential conflicts of interest and incentives for subjects.

14. The research protocol should always contain a statement of the ethical considerations

involved and should indicate that there is compliance with the principles enunciated in this Declaration.

15. Medical research involving human subjects should be conducted only by scientifically qualified persons and under the supervision of a clinically competent medical person. The responsibility for the human subject must always rest with a medically qualified person and never rest on the subject of the research, even though the subject has given consent.

16. Every medical research project involving human subjects should be preceded by careful assessment of predictable risks and burdens in comparison with foresee able benefits to the subject or to others. This does not preclude the participation of healthy volunteers in medical research. The design of all studies should be publicly available.

17. Physicians should abstain from engaging in research projects involving human subjects unless they are confident that the risks involved have been adequately assessed and can be satisfactorily managed. Physicians should cease any investigation if the risks are found to outweigh the potential benefits or if there is conclusive proof of positive and beneficial results.

18. Medical research involving human subjects should only be conducted if the importance of the objective outweighs the inherent risks and burdens to the subject. This is especially important when the human subjects are healthy volunteers.

19. Medical research is only justified if there is a reasonable likelihood that the populations in which the research is carried out stand to benefit from the results of the research.

20. The subjects must be volunteers and informed subjects in the research project.

21. The right of research subjects to safeguard their integrity must always be respected. Every precaution should be taken to respect the privacy of the subject, the confidentiality of the patient's information and to minimize the impact of the study on the subject's physical and mental integrity and on the personality of the subject.

22. In any research on human beings, each potential subject must be adequately informed of the aims, methods, sources of funding, any possible conflicts of interest, institutional affiliations of the researcher, the anticipated benefits and potential risks of the study and the discomfort it may entail. The subject should be informed of the right to abstain from participation in the study or to withdraw consent to participate at any time without reprisal. After ensuring that the subject has understood the information, the physician should then obtain the subject's freely-given informed

consent, preferably in writing. If the consent cannot be obtained in writing, the non-written consent must be formally documented and witnessed.

23. When obtaining informed consent for the research project the physician should be particularly cautious if the subject is in a dependent relationship with the physician or may consent under duress. In that case the informed consent should be obtained by a well-informed physician who is not engaged in the investigation and who is completely independent of this relationship.

24. For a research subject who is legally incompetent, physically or mentally incapable of giving consent or is a legally incompetent minor, the investigator must obtain informed consent from the legally authorized representative in accordance with applicable law. These groups should not be included in research unless the research is necessary to promote the health of the population represented and this research cannot instead be performed on legally competent persons.

25. When a subject deemed legally incompetent, such as a minor child, is able to give assent to decisions about participation in research, the investigator must obtain that assent in addition to the consent of the legally authorized representative.

26. Research on individuals from whom it is not possible to obtain consent, including proxy or advance consent, should be done only if the physical/mental condition that prevents obtaining informed consent is a necessary characteristic of the research population. The specific reasons for involving research subjects with a condition that renders them unable to give informed consent should be stated in the experimental protocol for consideration and approval of the review committee. The protocol should state that consent to remain in the research should be obtained as soon as possible from the individual or a legally authorized surrogate.

27. Both authors and publishers have ethical obligations. In publication of the results of research, the investigators are obliged to preserve the accuracy of the results. Negative as well as positive results should be published or otherwise publicly available. Sources of funding, institutional affiliations and any possible conflicts of interest should be declared in the publication. Reports of experimentation not in accordance with the principles laid down in this Declaration should not be accepted for publication。

C. Additional principles for medical research combined with medical care

28. The physician may combine medical research with medical care, only to the extent that the research is justified by its potential prophylactic, diagnostic or therapeutic value. When

medical research is combined with medical care, additional standards apply to protect the patients who are research subjects.

29. The benefits, risks, burdens and effectiveness of a new method should be tested against those of the best current prophylactic, diagnostic, and therapeutic methods. This does not exclude the use of placebo, or no treatment, in studies where no proven prophylactic, diagnostic or therapeutic method exists.

30. At the conclusion of the study, every patient entered into the study should be assured of access to the best proven prophylactic, diagnostic and therapeutic methods identified by the study.

31. The physician should fully inform the patient which aspects of the care are related to the research. The refusal of a patient to participate in a study must never interfere with the patient-physician relationship.

32. In the treatment of a patient, where proven prophylactic, diagnostic and therapeutic methods do not exist or have been ineffective, the physician, with informed consent from the patient, must be free to use unproven or new prophylactic, diagnostic and therapeutic measures, if in the physician's judgement it offers hope of saving life, reestablishing health or alleviating suffering. Where possible, these measures should be made the object of research, designed to evaluate their safety and efficacy. In all cases, new information should be recorded and, where appropriate, published. The other relevant guidelines of this Declaration should be followed.

Annex 10 The Risk Management Plan of Thalidomide to Prevent Pregnancy

1 Risk of pregnancy

Thalidomide is known to cause serious birth defects, including extremity disability, deformity of legs and arms or fetal death, so the risk management plan must be rigorously followed to prevent pregnancy.

2 Classification of the participants

Participants participating in this trial are divided into three types: fertile female participants, infertile female participants and male participants. Fertile female participants refer to sexually mature ones, including those who haven't performed a hysterectomy or bilateral oophorectomy before and who haven't experienced physiological amenorrhoea for at least 24 consecutive months, namely who have menstruation at any time within the previous 24 months (amenorrhea due to cancer treatment can not exclude the possibility of fertility).

3 Medication guidance for participants

Before starting the treatment of thalidomide, researchers must ensure that participants of different types fully understand the following risks and requirements and take the participant's request of commitment to comply with the risk management plan of thalidomide to prevent pregnancy as one of the enrollment criteria.

1) Fertile female participants:

a. Considering that thalidomide has potential risk of teratogenicity and lethality to fetuses, pregnancy is forbidden during the period of taking thalidomide;

b. Take continuous and effective contraceptive measures or suppress sensual passion completely within 28 days before treatment, during the treatment phase, during the interruption period of administration and within 28 days after stopping taking thalidomide;

c. Pregnancy testing should be carried out in accordance with the provisions of the protocol;

d. Notify researchers in time if pregnant;

e. Thalidomide is forbidden to share with others and all unused drugs must be returned to researchers at the end of each course of treatment;

f. Blood donation is forbidden during the period of taking thalidomide and within 28 days after stopping taking thalidomide;

g. Breastfeeding is forbidden during the period of taking thalidomide and within 28 days after

stopping taking thalidomide.

2) Infertile female participants:

a. Considering that thalidomide has potential risk of teratogenicity and lethality to fetuses, pregnancy is forbidden during the period of taking thalidomide;

b. Thalidomide is forbidden to share with others and all unused drugs must be returned to researchers at the end of each course of treatment;

c. Blood donation is forbidden during the period of taking thalidomide and within 28 days after stopping taking thalidomide.

3) Male participants:

a. There is a potential teratogenic risk when having sexual intercourse with a fertile woman;

b. Condoms are still needed when having sexual intercourse with a fertile woman, even if you have got a vasectomy;

c. Notify researchers immediately if the sexual partner is pregnant or the pregnancy test is positive during the trial;

d. Thalidomide is forbidden to share with others and all unused drugs must be returned to researchers at the end of each course of treatment;

e. Blood donation and sperm/semen donation are forbidden during the period of taking thalidomide and within 28 days after stopping taking thalidomide.

4 Contraceptive measures

Fertile female participants must take two reliable contraceptive measures or suppress sensual passion completely during the following study periods:

1) Within at least 28 days before taking the test drug;

2) During treatment;

3) Within at least 28 days after stopping the trial.

The two reliable contraceptive measures must include an high-efficient measure and an effective auxiliary measure (barrier). The following are several high-efficient and effective auxiliary contraceptive measures:

1) High-efficient measures

a. Intrauterine device (IUD);

b. Hormones (such as oral, injectable or implanted contraceptives);

c. Tubal ligation;

d. Vasectomy of the sexual partner.

2) Effective auxiliary measures

a. Condoms;

b. Diaphragm of the uterus;

c. Cervical cap.

Patients with multiple myeloma have a higher risk of venous thromboembolism and oral contraceptives or thalidomide can increase this risk, therefore oral contraceptives are not recommended in the trial. Other effective measures are supposed to be taken instead if the participants are taking oral contraceptives. It should be noted that the risk of venous thrombosis can continue 4-6 weeks after stopping oral contraceptives.

The male participant must wear a condom for contraception during the trial and within 28 days after stopping the trial, even if he has got a vasectomy before.

5 Pregnancy testing

Fertile women (including those who guarantee to suppress sensual passion completely) must take a medically supervised pregnancy test with the sensitivity of at least 25 mlU/ml before starting the trial, and promise to take reliable contraceptive measures or suppress sensual passion completely during treatment. The timing of pregnancy test is arranged as follows:

1) Before the randomization at the screening period, at least one pregnancy test should be performed on the premise of taking reliable contraceptive measures or suppressing sensual passion completely for no less than 28 days. If the contraceptive measures are judged to be unreliable, retest should be carried out with an interval of 10-14 days;

2) Pregnancy test should be performed within 1-3 days before the first treatment. And there is no need to retest if participants have the results within 1-3 days of screening period;

3) Before each course of treatment during the trial;

4) Take a test on the 28th day after taking the last dose of thalidomide (the pregnancy test can be performed at the nearby medical institution for patients who are inconvenient to come to the testing center and the positive testing results should be reported to researchers and recorded as a SAE).

5) Participants and researchers can increase the testing frequency according to the actual

situation.

6 Risk management measures during the trial

I. Before treatment

1) Researchers should fill in the “Thalidomide Medication Guidance” (see Annex 11) and provide the participants with risk education on safety information of thalidomide, relevant requirements of pregnancy risk test and matters needing attention. Both doctors and patients should sign.

2) Researchers should distribute the “Thalidomide Information Sheet” (see Annex 12) to all participants, which summarizes the important information about thalidomide that participants should learn about during the trial and issues need to be noticed when taking thalidomide.

3) Before the randomization at the screening period, at least one pregnancy test should be performed on the premise of taking reliable contraceptive measures or suppressing sensual passion completely for no less than 28 days for the fertile women. If the contraceptive measures are judged to be unreliable, retest should be carried out with an interval of 10-14 days. Pregnancy test should be performed within 1-3 days before the first treatment. And there is no need to retest if participants have the results within 1-3 days of screening period;

4) Develop a daily diary card of medicine taking for participants and instruct them to make detailed records of the daily dose, time, and whether discomfort occurs at each course of the treatment;

5) Develop an emergency response plan (see Annex 13) to make all participants clearly understand the disposal measures if pregnancy and other events occur.

II. During treatment

1) Fertile female participants must continuously take reliable contraceptive measures or suppress sensual passion completely during treatment;

2) Fertile female participants should take pregnancy test before each course of treatment. Researchers should fill in the “Thalidomide Medication Guidance” (see Annex 11) before distributing thalidomide at each course of treatment, and provide the participants with risk education on safety information of thalidomide, relevant requirements of pregnancy risk test and matters needing attention. Both doctors and patients should sign.

3) Participants must read the “Thalidomide Information Sheet” (see Annex 12) before geting

thalidomide for each new course of treatment;

4) Only one course of thalidomide is allowed to distribute for each course of treatment;

5) Researchers must carefully check the amount of thalidomide's distribution, usage, and recycling according to the participant's daily diary card. They should also find and trace the problems in time and illuminate the solutions of the incidents to avoid unknown flow of the drug;

6) It is recommended to terminate the pregnancy and the clinical trial if the pregnancy test of a fertile woman or the sexual partner of a male participant is found to be positive during the trial. Report it as a serious adverse event and the treatment comes to the end;

7) Researchers should inform fertile female participants of suppressing sensual passion completely or continuously taking two reliable contraceptive measures within 28 days after the last administration of thalidomide and avoiding breastfeeding. At the same time, male participants still need to wear a condom for contraception within 28 days after the last administration of thalidomide and avoid sperm donation;

8) Researchers should inform all participants of avoiding blood donation within 28 days after the last administration of thalidomide;

9) It is required that the fertile woman should take a pregnancy test on the 28th day after stopping taking thalidomide to ensure safety (the pregnancy test can also be performed at the nearby medical institution for patients who are inconvenient to come to the testing center and the positive testing results should be reported to researchers and recorded as a SAE).

Annex 11 Thalidomide Medication Guidance

Female: ☐

If you are a female participant, choose 1

☐ Fertile women: refer to sexually mature ones, including 1) women who have't performed a hysterectomy or bilateral oophorectomy before or 2) women who haven't experienced physiological amenorrhoea for at least 24 consecutive months, namely who have menstruation at any time within the previous 24 months (amenorrhea due to cancer treatment can not exclude the possibility of fertility)

☐ Infertile women:

Male:

Researchers must ensure the following items before distributing thalidomide:

I. For fertile female participants

1. The result of pregnancy test is negative before the first treatment

2. Fertile women have been informed about the following related instructions:

1) It is forbidden to be pregnant during the period of taking thalidomide, because taking thalidomide during pregnancy can cause birth defects or stillbirths;

2) Suppress sensual passion completely or take an high-efficient measure and an auxiliary measure (barrier) simultaneously for contraception at least within 28 days before treatment, during the treatment phase, during the interruption period of administration and within 28 days after stopping taking thalidomide;

a. High-efficient measures, for example, intrauterine device (IUD), hormones (such as oral, injectable or implanted contraceptives), tubal ligation or vasectomy of the sexual partner;

b. Effective auxiliary measures, for example, condoms, diaphragm of the uterus or cervical cap.

3) Testing frequency

a. Before the randomization at the screening period, at least one pregnancy test should be performed on the premise of taking reliable contraceptive measures or suppressing sensual passion completely for no less than 28 days for the fertile women. If the contraceptive measures are

judged to be unreliable, retest should be carried out with an interval of 10-14 days.

b. Pregnancy test should be performed within 1-3 days before the first treatment. And there is no need to retest if participants have the results within 1-3 days of screening period;

c. Before each course of treatment during the trial;

d. Take a test on the 28th day after taking the last dose of thalidomide (the pregnancy test can be performed at the nearby medical institution for patients who are inconvenient to come to the testing center and the positive testing results should be reported to researchers and recorded as a SAE).

4) Stop taking thalidomide once pregnant or the pregnancy test is positive and report it to researchers immediately;

5) Do not share the drug with others;

6) All unused drugs are supposed to be returned to researchers at the end of each course of treatment;

7) Blood donation is forbidden during the period of taking thalidomide and within 28 days after stopping taking thalidomide;

8) Breastfeeding is forbidden during the period of taking thalidomide and within 28 days after stopping taking thalidomide;

9) Do not chew thalidomide tablets;

10) Participants and researchers can increase the testing frequency according to the actual situation.

3. Participants have been provided with the “Thalidomide Information Sheet”.

II. For infertile female participants

1. Infertile women have been informed about the following related instructions:

1) It is forbidden to be pregnant during the period of taking thalidomide, because taking thalidomide during pregnancy can cause birth defects or stillbirths;

2) Do not share the drug with others;

3) All unused drugs are supposed to be returned to researchers at the end of each course of treatment;

4) Blood donation is forbidden during the period of taking thalidomide and within 28 days after stopping taking thalidomide;

5) Do not chew thalidomide tablets.

2. Participants have been provided with the “Thalidomide Information Sheet”.

III. For male participants

1. Male participants have been informed about the following instructions:

1) It is forbidden to be pregnant for the sexual partner during the period of taking thalidomide, because taking thalidomide during pregnancy can cause birth defects or stillbirths;

2) Wear condoms when having sexual intercourse with a fertile woman (including the participant who has got a vasectomy) or suppress sensual passion completely during the period of taking thalidomide and within 28 days after stopping taking thalidomide

3) Notify researchers immediately if the sexual partner of a male participant taking thalidomide is pregnant or the pregnancy test is positive;

4) Do not share the drug with others;

5) All unused drugs are supposed to be returned to researchers at the end of each course of treatment;

6) Blood donation and sperm/semen donation are forbidden during the period of taking thalidomide and within 28 days after stopping taking thalidomide;

7) Do not chew thalidomide tablets.

2. Participants have been provided with “Thalidomide Information Sheet”.

Researcher’s signature :

Date:

Participant’s declaration:

The information I provide to the researchers is true and I have received the thalidomide medication guidance. I am aware of the risks associated with taking thalidomide and the necessary prevention measures. I commit to complying with the relevant requirements.

Participant’s signature:

Date:

Annex 12 Thalidomide Information Sheet

The thalidomide information sheet provides important information about the use of thalidomide, and participants are supposed to read it carefully before participating in the trial and every time they get thalidomide. It should be noted that the sheet can not replace the informed consent for the clinical trial. Questions about physical condition or treatment should be communicated with researchers.

Pay attention to the following issues when using thalidomide:

I. Thalidomide can cause birth defects (deformed foetus), including extremity disability, deformity of legs and arms, or fetal death

1. For fertile female participants:

1) Participants who are already pregnant or prepare for pregnancy can not participate in this trial;

2) Pregnancy test must be performed at the following times:

a. Before the randomization at the screening period, at least one pregnancy test should be performed on the premise of taking effective contraceptive measures or suppressing sensual passion completely for no less than 28 days. If the contraceptive measures are not reliable, retest should be carried out with an interval of 10-14 days;

b. Pregnancy test should be performed within 1-3 days before the first treatment. And there is no need to retest if participants have the results within 1-3 days of screening period;

c. Before each course of treatment during the trial;

d. Take a test on the 28th day after taking the last dose of thalidomide (the pregnancy test can also be performed at the nearby medical institution for patients who are inconvenient to come to the testing center and the positive testing results should be reported to researchers and recorded as a SAE).

3) Participants must suppress sensual passion completely or take two different types of reliable contraceptive measures simultaneously at the following times:

a. Within 28 days before taking thalidomide;

b. During the period of taking thalidomide;

c. During the interruption period of taking thalidomide;

d. Within 28 days after stopping taking thalidomide.

4) Consult researchers about contraceptive measures;

5) Participants who are suspected to be pregnant or the pregnancy test is positive during the trial must stop taking thalidomide immediately. Notify researchers in time and report to Changzhou Pharmaceutical Factory;

6) Thalidomide is forbidden to share with others and should be kept out of reach of children;

7) All unused drugs are supposed to be returned to researchers at the end of each course of treatment;

8) Breastfeeding is forbidden during the period of taking thalidomide and within 28 days after stopping taking thalidomide;

9) Blood donation is forbidden during the period of taking thalidomide and within 28 days after stopping taking thalidomide;

10) The amount of medicine got each time should not exceed 28 days;

11) Do not chew thalidomide tablets.

2. For infertile female participants:

1) Thalidomide is forbidden to share with others and should be kept out of reach of children;

2) All unused drugs are supposed to be returned to researchers at the end of each course of treatment;

3) Blood donation is forbidden during the period of taking thalidomide and within 28 days after stopping taking thalidomide;

4) The amount of medicine got each time should not exceed 28 days;

5) Do not chew thalidomide tablets.

3. For male participants, thalidomide can exist in semen, so:

1) Wear a condom while having sexual intercourse with a pregnant or fertile woman or suppress sensual passion completely at the following times, including the participant who has got a vasectomy:

a. During the period of taking thalidomide;

b. Interruption period of taking thalidomide;

c. Within 28 days after stopping taking thalidomide.

2) Notify researchers in time and report to Changzhou Pharmaceutical Factory if the sexual partner is suspected to be pregnant or the pregnancy test is positive at any time during the trial;

3) Thalidomide is forbidden to share with others and should be kept out of reach of children;

4) All unused drugs are supposed to be returned to researchers at the end of each course of treatment;

5) Sperm or semen donation is forbidden during the period of taking thalidomide and within 28 days after stopping taking thalidomide;

6) Blood donation is forbidden during the period of taking thalidomide and within 28 days after stopping taking thalidomide;

7) The amount of medicine got each time should not exceed 28 days;

8) Do not chew thalidomide tablets.

II. Given that thalidomide can cause decreases in the number of leukocytes and platelets and lead to infection and bleeding, blood transfusion and drug treatment may be required;

III. Thalidomide can cause thrombosis in veins and lungs, so notify researchers immediately or take an emergency treatment once shortness of breath, chest pain, or limb swelling happens.

Additional information is provided in the informed consent form and consult researchers further if there are any doubts or other questions.

Annex 13 Emergency Response Plan for Pregnancy

If a fertile female participant or the sexual partner of a male participant has pregnancy reaction or suspected pregnancy reaction (including a positive pregnancy test, regardless of age or disease status) during the medication period or within 28 days after the last medication, it must be processed and reported in accordance with the requirements of this plan.

1. When pregnancy, suspected pregnancy or positive pregnancy test is found, researchers must report the SAE to the sponsor, work unit of the study leader and CFDA in writing immediately (see the table for the specific list and contact information);

2. Participants must be instructed to stop treatment immediately and return all the unused drugs to researchers. Male participants can continue to receive treatment unless they guarantee to suppress sensual passion completely;

3. It is recommended that the pregnant participant or sexual partner of the male participant terminate the pregnancy;

4. The pregnant women must be followed up to determine the outcome until the pregnancy is terminated, including spontaneous or voluntary termination of pregnancy, details of childbirth, whether birth defects or maternal and/or neonatal comorbidities exist. And the pregnancy results will serve as a follow-up supplement to the initial SAE report:

1) If the pregnancy results meet the SAE classification criteria, such as spontaneous abortion or artificial abortion (any congenital malformations of aborted fetuses detected must be recorded), stillbirths, neonatal deaths or congenital malformations (including congenital malformations of aborted fetuses), researchers must report SAEs according to the procedure within 24 hours to the sponsor, work unit of the study leader and CFDA in writing immediately;

2) If there are “normal” infants alive, researchers must report to the sponsor, work unit of the study leader and CFDA in writing within 24 hours of learning about the event;

3) All neonatal deaths occurring within 28 days of birth should be recorded as SAEs, regardless of whether there exists a causal relationship between the death and the test drug;

4) Any infants death after 28 days of birth should be reported to the sponsor, work unit of the study leader and CFDA in writing within 24 hours of learning about the event, if researchers believe that the death is related to the intrauterine exposure to the test drug.

5. If it is found that the female participant or the sexual partner of a male participant is not

pregnant, the researcher and the sponsor jointly determine whether the participant should continue to participate in the trial.
